# Coupling dead cell recognition to Fcγ receptors augments anticancer immunity

**DOI:** 10.1038/s43018-026-01168-5

**Published:** 2026-05-20

**Authors:** Tomás Castro-Dopico, Cécile Piot, Michael D. Buck, Lucía Gandullo-Sánchez, Kok Haw Jonathan Lim, Gerone A. Gonzales, Nikita Rosendahl, Oliver Schulz, William Stainier, Brian Vash, Stephanie Maiocco, Conor M. Henry, Bruno Frederico, Sonia Lee, Andy Bo Zhang, Neil C. Rogers, Jayanta Bordoloi, Chloë Roustan, Svend Kjaer, Mohit Trikha, Hans Stauss, Ahmet Hazini, Kristen J. Radford, Zewen Kelvin Tuong, Johnathan Canton, Raj Mehta, Caetano Reis e Sousa

**Affiliations:** 1 Immunobiology Laboratory, The Francis Crick Institute, London, UK; 2 Department of Immunology and Inflammation, Imperial College London, London, UK; 3 Faculty of Veterinary Medicine, University of Calgary, Calgary, Alberta Canada; 4 Mater Research Institute, The University of Queensland, Translational Research Institute, Brisbane, Queensland Australia; 5 Apple Tree Partners, Cambridge, MA USA; 6 In vivo Imaging, The Francis Crick Institute, London, UK; 7 Structural Biology, The Francis Crick Institute, London, UK; 8 Drug Design Corp., San Francisco, CA USA; 9 Institute of Immunology & Transplantation, University College London, London, UK; 10 Department of Oncology, University of Oxford, Oxford, UK; 11 Ian Frazer Centre for Children’s Immunotherapy Research, Child Health Research Centre, Faculty of Health, Medicine and Behavioural Sciences, The University of Queensland, Brisbane, Queensland Australia; 12 Adendra Therapeutics Ltd, London, UK; 13 Present Address: Division of Cancer Sciences, University of Manchester & Advanced Immunotherapy and Cell Therapy Team, The Christie NHS Foundation Trust, Manchester, UK; 14 Present Address: Roche Products Limited, Welwyn Garden City, UK; 15 Present Address: The Discovery Centre, Cambridge Biomedical Campus, Cambridge, UK

**Keywords:** Tumour immunology, Cancer immunotherapy, Antigen processing and presentation, Cancer

## Abstract

Type 1 conventional dendritic cells (cDC1s) are key antigen-presenting cells (APCs) for cross-priming of CD8^+^ T cells against cancer. They can capture and cross-present dead cell antigens via DNGR-1 (CLEC9A), a receptor for F-actin exposed on cell corpses. However, cDC1s are scarce in human and murine tumors, and this restricts anticancer immunity. We show that abundant tumor-associated APCs, including cDC2s and monocyte-derived cells, can be redirected to internalize necrotic debris and cross-present associated antigens by a variety of reagents that bridge F-actin to Fcγ receptors (FcγRs), including an Fc–DNGR-1 fusion protein and an anti-F-actin antibody. In vivo, Fc–DNGR-1 accumulates in necrotic tumor areas and highlights their proximity to intratumoral FcγR^+^ APCs. In mouse cancer models, F-actin–FcγR bridging enhances tumor control and synergizes with cytotoxic chemotherapy or radiotherapy. Thus, nonspecialized APCs can be harnessed for cross-presentation of necrotic tumor antigens, and F-actin–FcγR bridging constitutes a strategy to potentiate anticancer immunity.

## Abstract (web summary)

Castro-Dopico et al. report the design of reagents to bridge F-actin and Fcγ receptors, endowing a range of antigen-presenting cells with the ability to cross-present antigens from dead tumor cells and boosting antitumor immunity in preclinical models.

## Main

Type 1 conventional dendritic cells (cDC1s) are essential for induction of cytotoxic CD8^+^ T lymphocytes (CTLs) and for the efficacy of immune checkpoint blockade in a variety of murine tumors^1–7^. In humans, tumor cDC1 abundance is correlated with CTL infiltration and favorable outcomes in cancer patients^8,9^. A key attribute of cDC1s is their ability to acquire and cross-present tumor cell-associated antigens on MHC class I (MHC-I) molecules for priming of cancer-specific CTLs^2,4,10,11^. A notable source of tumor antigens is debris from cancer cells that die spontaneously or in response to therapy^12^. Compared to other antigen-presenting cells (APCs), including cDC2s^13,14^, cDC1s excel at internalizing these dead cell remnants and express high levels of DNGR-1 (also known as CLEC9A), a C-type lectin receptor (CLR) that binds to F-actin exposed on the debris^15^. Triggering of DNGR-1 by F-actin within phagosomes causes signaling via SYK to promote local membrane destabilization, which can result in subsequent phagosomal rupture^16^. In this manner, dead-cell-derived antigens can be released into the cytosol (phagosome-to-cytosol translocation (P2C)) to enter the endogenous MHC-I presentation pathway. Notably, enforced expression of DNGR-1 in heterologous cells that express SYK can endow these cells with the ability to cross-present dead-cell-associated antigens^16^. This indicates that part of the specialized cross-presenting ability of cDC1s results from signaling by cDC1-specific receptors that link necrotic cell detection to the SYK pathway to induce P2C^15^.

The scarcity of cDC1s in tumors limits anticancer immunity^8,17,18^, and approaches to boost cDC1 numbers are being pursued for therapeutic purposes^11,19,20^. Coaxing other and more numerous intratumoral APCs to cross-present dead cancer-cell-associated antigens could represent an alternative strategy. Activatory Fcγ receptors (FcγRs) can be broadly expressed by APCs and signal via SYK and have been reported to activate myeloid cells, as well as promoting cross-presentation (XP) of IgG-bound cargo^21–23^. Indeed, adoptive transfer of GM-CSF-elicited bone marrow APCs loaded with IgG-opsonized ovalbumin (OVA) ex vivo protects against challenge with OVA-expressing tumors in mice^24^. As such, we hypothesized that coupling dead cell recognition to FcγR signaling would endow a range of non-cDC1 APCs with the ability to sample and cross-present antigens from dead tumor cells, to become activated and remodel the tumor microenvironment (TME) and, overall, to boost anticancer immunity.

## Results

### FcγR^+^ APCs are abundant within tumors and cross-present IgG-bound proteins

**Fig. 1:**
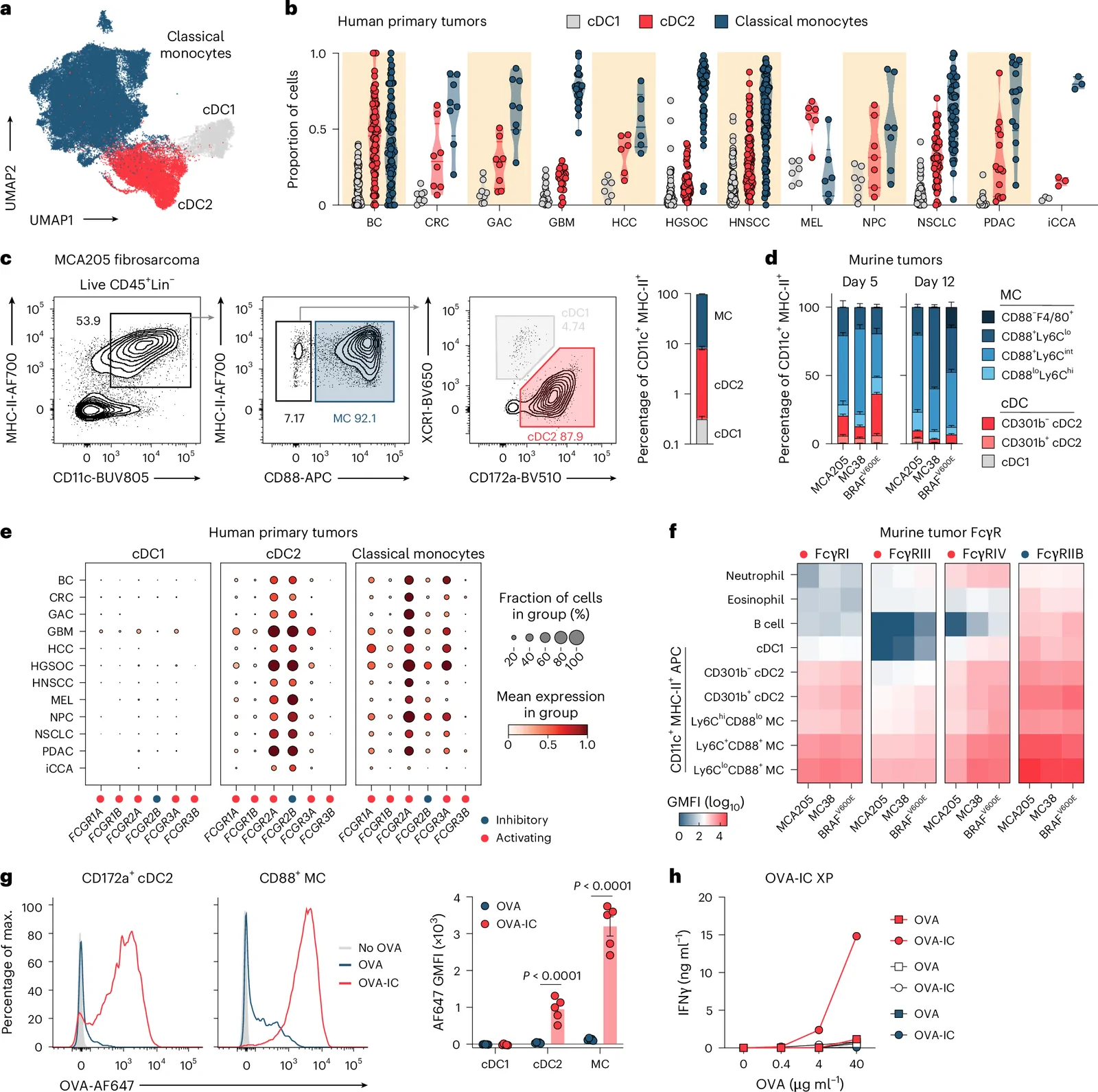
FcγR^+^ APCs are abundant within tumors and cross-present IgG-bound proteins. **a**,**b**, Human cDC and classical monocyte UMAP (**a**) and frequency across primary tumors (**b**), derived from a scRNA-seq pan-cancer myeloid APC atlas^26^. Proportions are relative to total cDC1s, cDC2s and classical monocytes. Breast cancer (BC, *n* = 74); colorectal carcinoma (CRC, *n* = 8); gastric adenocarcinoma (GAC, *n* = 8); glioblastoma (GBM; *n* = 23); hepatocellular carcinoma (HCC, *n* = 6); high-grade serous ovarian carcinoma (HGSOC, *n* = 69); head and neck squamous cell carcinoma (HNSCC, *n* = 98); melanoma (MEL, *n* = 6); nasopharyngeal carcinoma (NPC, *n* = 7); nonsmall-cell lung cancer (NSCLC, *n* = 51); pancreatic ductal adenocarcinoma (PDAC, *n* = 14); intrahepatic cholangiocarcinoma (iCCA, *n* = 3). Median values with quartiles are shown in violin plots. **c**, Flow cytometric analysis of mouse intratumoral APC frequency 10 days after implantation with 0.5 × 10^6^ MCA205 tumor cells (*n* = 6 mice). The mean ± s.e.m. is plotted. **d**, Quantification of APC subset frequency across time (day 5 and day 12 postimplantation) and tumor model (MCA205, MC38, and BRAF^V600E^) in C57BL/6 mice (*n* = 3 (MCA205 day 5), *n* = 4 (BRAF^V600E^ days 5 and 12) or *n* = 5 (MCA205 day 12 and MC38 days 5 and 12) mice per group). The mean ± s.e.m. is plotted. **e**, *FCGR* gene expression levels on human cDC and classical monocyte populations across tumor sites (data derived from scRNA-seq dataset in **a**). Gene expression values were capped at 1. **f**, Flow cytometric analysis of FcγR expression in mouse immune cell populations defined in Extended Data Fig. 1c and nonmyeloid APC populations. Mouse numbers are as in **d**. The mean GMFI is plotted. **g**, Flow cytometric analysis of ex vivo uptake of soluble OVA (blue) or OVA-IC (red) by APCs isolated from day 10 MCA205 tumors (*n* = 5 mice). The mean ± s.e.m. is plotted. **h**, ELISA for IFNγ release from OT-I CD8^+^ T cells cocultured with mouse splenic or intratumoral APCs pulsed with a titration of OVA or OVA-IC. The mean from technical duplicates is plotted. Data are representative of two (**d**, **g** and **h**) or three (**c** and **f**) biologically independent experiments. Data in **g** were analyzed using Bonferroni-corrected two-way ANOVA. Max., maximum.

**Extended Data Fig. 1:**
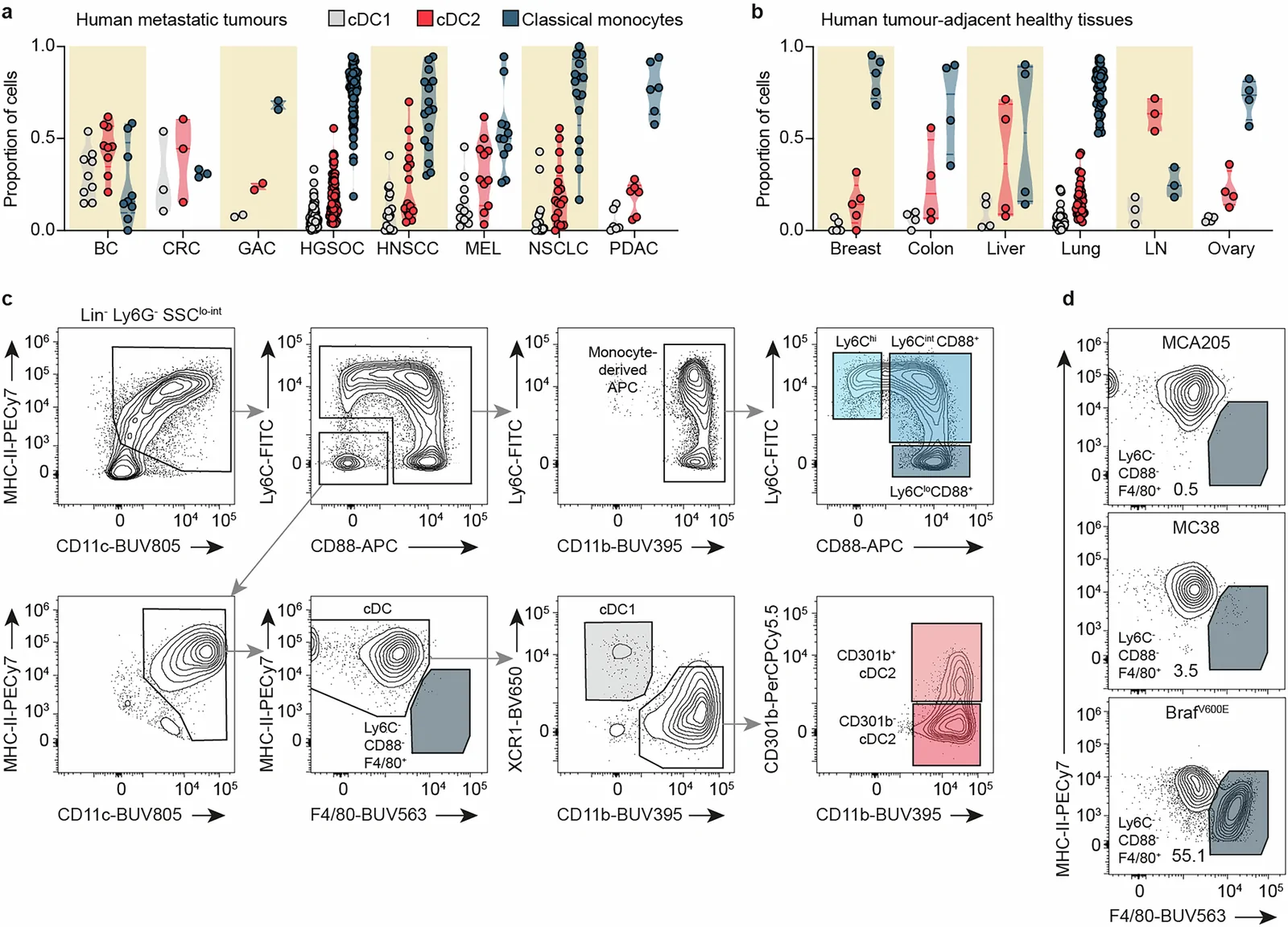
cDC2s and MCs are abundant in human and mouse tumours. (**a**, **b**) Human cDC and classical monocyte frequency across metastatic tumour sites (**a**) and healthy tissues (**b**), derived from a scRNAseq pan-cancer myeloid APC atlas^26^. Proportion relative to total cDC1s, cDC2s, and classical monocytes. Metastatic tissues: BC = breast cancer (*n* = 9); CRC = colorectal carcinoma (*n* = 3); GAC = gastric adenocarcinoma (*n* = 2); HGSOC = high-grade serous ovarian carcinoma (*n* = 73); HNSCC = head and neck squamous cell carcinoma (*n* = 16); MEL = melanoma (*n* = 11); NSCLC = non-small cell lung cancer (*n* = 17); PDAC = pancreatic ductal adenocarcinoma (*n* = 6). Healthy tissues: breast (*n* = 5); colon (*n* = 4); liver (*n* = 4); lung (*n* = 34); LN = lymph node (*n* = 3); ovary (*n* = 4). Median with quartiles on violin plots is plotted. (**c**) Flow cytometric gating strategy for identification of major APC subsets in tumours (day 12 MCA205 tumours shown). (**d**) Flow cytometry as in **c**, showing Ly6C^*−*^ CD88^*−*^ F4/80^+^ cells across tumour models. Plots gated on MHC-II^+^ CD11c^+^ Ly6C^*−*^ CD88^*−*^ cells. Data are representative of two (**d**) or three (**c**) biologically independent experiments.

cDC1s belong to a broader family of APCs that includes cDC2s, CCR7^+^/LAMP3^+^ dendritic cells (DCs) and monocyte-derived cells (MCs)^25^. We assessed their relative abundance within the TME in humans using a recently generated pan-cancer myeloid cell atlas that integrates 36 single-cell RNA sequencing (scRNA-seq) datasets, covering 498,023 cells, 589 samples and 12 primary tumor types^26^. Within this dataset, and in agreement with previous results^9,27^, we observed that cDC1s were outnumbered by cDC2s across multiple cancers (Fig. 1a,b). This numerical disadvantage was maintained in metastatic tumor tissues and tumor-adjacent healthy tissues (Extended Data Fig. 1a,b). In turn, cDC2s were outnumbered by classical monocytes across most sites. We also assessed APC frequency in a murine MCA205 transplantable cancer model. Again, both CD88^+^ MCs and CD172a^+^ cDC2s vastly outnumbered XCR1^+^ cDC1s among CD11c^+^MHC-II^+^ APCs (<1% cDC1s at day 10) (Fig. 1c).

Both cDC2s and MCs comprise various subsets and/or cell states. For more comprehensive analysis, we performed high-dimensional flow cytometry analysis of tumor-infiltrating APCs in different mouse tumor models (MCA205, MC38, BRAF^V600E^) and time points (day 5 and day 12), identifying 7 distinct CD11c^+^ MHC-II^+^ populations (Extended Data Fig. 1c,d). CD11b^+^ MCs were divided into three populations based on progressive acquisition of CD88 and loss of Ly6C, akin to the ‘monocyte waterfall’ reported in other tissues^28^. cDC2s were also subdivided based on expression of CD301b. Notably, although both types of cDC2 represented a substantial fraction of the APC pool in incipient tumors, they were progressively outnumbered by differentiated MCs (that is, CD88^+^ cells) over time (Fig. 1d). Irrespective, across all time points and tumors, cDC1s represented less than 1% of tumor myeloid APCs. Overall, we confirm that cDC1s are exceedingly rare relative to cDC2s and MCs in human and murine tumors and show that MCs progressively accumulate in growing tumors.

**Extended Data Fig. 2:**
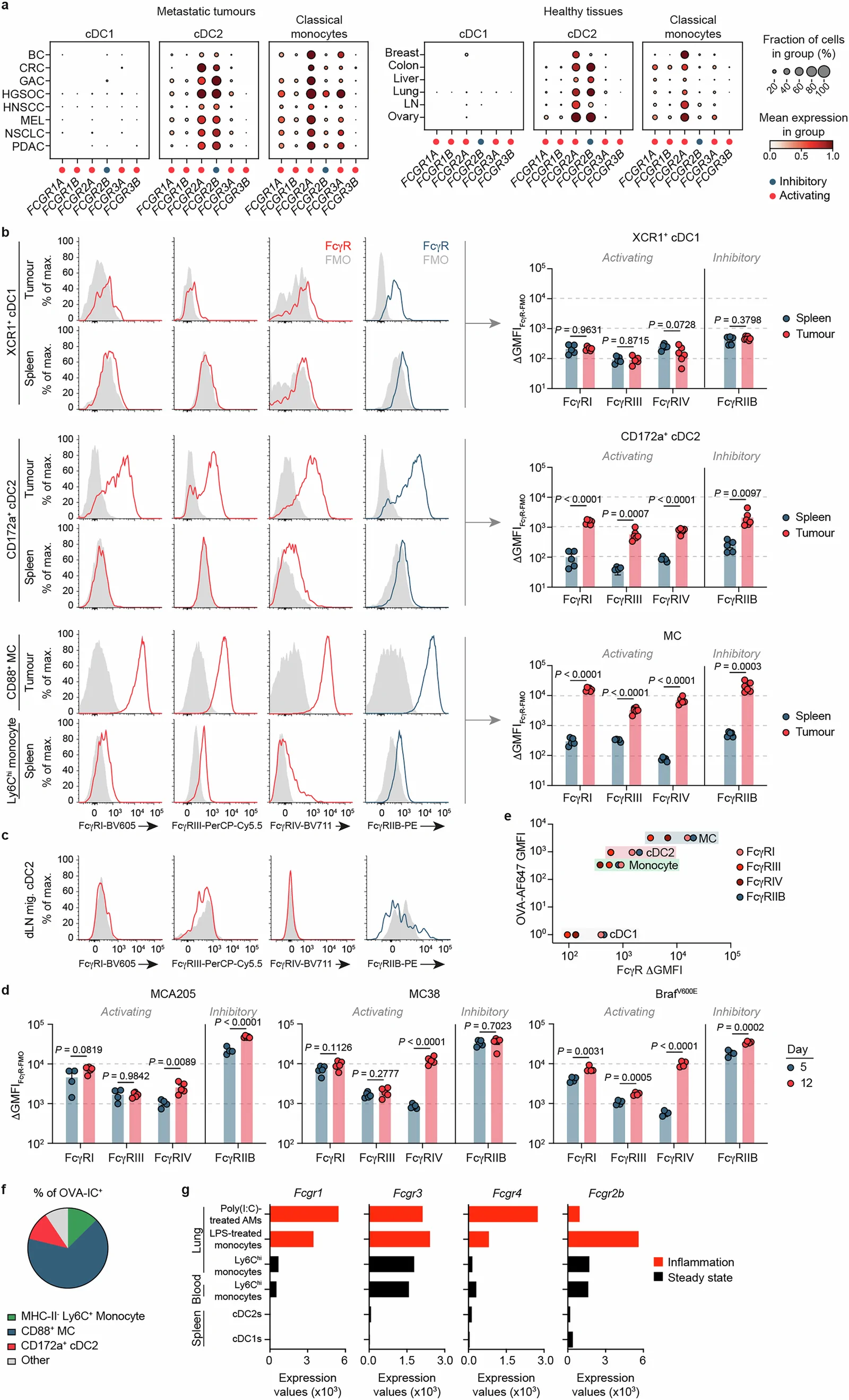
Abundant tumour APCs express FcγRs. (**a**) *FCGR* gene expression levels on human cDCs and classical monocytes populations across metastatic tumour sites and healthy tissues (data derived from^26^). Gene expression values capped at 1. Sample numbers as per Extended Data Fig. 1a and b. (**b**, **c**) Flow cytometric analysis of FcγR expression in mouse splenic and intra-tumoural APC populations (**b**) and tumour-draining lymph nodes (**c**) in MCA205-bearing mice (day 10) (*n* = 5 (spleen) or 6 (tumour) per group). Mean of ΔGMFI ± s.e.m. is plotted (GMFI(sample) - GMFI (FMO), using FMO controls acquired under identical settings to account for background). (**d**) Flow cytometric analysis of FcγR expression on intra-tumoural CD11c^+^ MHC-II^+^ Ly6C^lo^ CD88^+^ APCs across early (day 5) and late (day 12) timepoints and different tumour models (*n* = 4 (MCA205 day 5, Braf^V600E^ day 5 and 12) or 5 (MCA205 day 12, MC38 day 5 and 12) mice per group). Mean ± s.e.m. is plotted. (**e**) Correlation of OVA-IC internalisation levels *ex vivo* with FcγR expression profile in MCA205 tumour-infiltrating APCs. (**f**) Flow cytometric analysis of uptake of OVA-IC by all *ex vivo* immune cell subsets isolated from MCA205 tumours in **d**. (**g**) Analysis of *Fcgr* transcript levels in steady state and inflammatory myeloid APCs, including cDC1s, cDC2s, and monocyte-derived cells. Data were derived from the Immgen database. Data are representative of two (**d**-**f**) or three (**b**, **c**) biologically independent experiments. Data were analysed using a two-tailed Student’s *t* test (**b**, **d**).

Transcript profiling in humans revealed that both cDC2s and classical monocytes expressed FcγRs across healthy and tumor tissue sites (notably activatory FcγRIIA and inhibitory FcγRIIB in cDC2s), whereas cDC1s were generally devoid of FcγR expression despite intertissue heterogeneity (Fig. 1e and Extended Data Fig. 2a). In mice, under noninflammatory conditions, cDCs typically express only low levels of FcγRs (for example, FcγRIV and FcγRIIB on splenic cDC2s)^29^, but we observed high FcγR expression by CD88^+^ MCs and cDC2s within the TME, particularly for activatory FcγRI and FcγRIV and inhibitory FcγRIIB (Fig. 1f and Extended Data Fig. 2b). This was in contrast to spleen or tumor-draining lymph nodes of tumor-bearing mice, in which FcγR staining of cDC2 remained low in comparison to staining of cDC2 in the TME when identical acquisition parameters were used across all tissues (Extended Data Fig. 2b,c and Supplementary Table 1). Murine cDC1s expressed low levels of FcγR irrespective of tissue of origin, as in humans (Extended Data Fig. 2b). FcγR expression levels increased between incipient and established tumors, as exemplified for Ly6C^lo^ CD88^+^ MCs (Extended Data Fig. 2d and Supplementary Table 2). This was most notable for FcγRIV and to a lesser extent for FcγRI and FcγRIIB, whereas FcγRIII remained largely unchanged across time points. Notably, FcγRs expressed by cDC2s and MCs isolated from murine tumors were functional as they mediated uptake of OVA immune complexes (ICs) (Fig. 1g), with the extent of uptake mirroring surface FcγR expression levels (Extended Data Fig. 2e). Little uptake was observed by non-APC (CD11c^+/−^MHC-II^−^) (Extended Data Fig. 2f). OVA-IC was efficiently cross-presented to CD8^+^ T cells by CD11c^+^MHC-II^+^CD88^+^ MCs extracted from tumors but only poorly cross-presented by the equivalent APCs taken from spleen (Fig. 1h). To extend our findings, we analyzed *Fcgr* transcript levels in murine APCs across steady state and inflamed tissues in the Immgen database. Consistent with our observations, splenic cDC2s expressed only very low levels of *Fcgr4* and *Fcgr2b* in steady state, whereas monocytes expressed low-to-intermediate levels of all FcγRs (Extended Data Fig. 2g). By contrast, APCs isolated from inflamed tissues showed high FcγR expression levels, most notably activating FcγRI and FcγRIV and inhibitory FcγRIIB, consistent with our observations for CD88^+^ MCs in tumors. Therefore, FcγRs are highly expressed by non-cDC1 APCs in tumors and in inflamed tissues across species, allowing uptake of IgG ICs and inducible XP to CD8^+^ T cells in mice.

### Fc–CLRs couple necrotic cell binding to necrophagy and XP

**Fig. 2:**
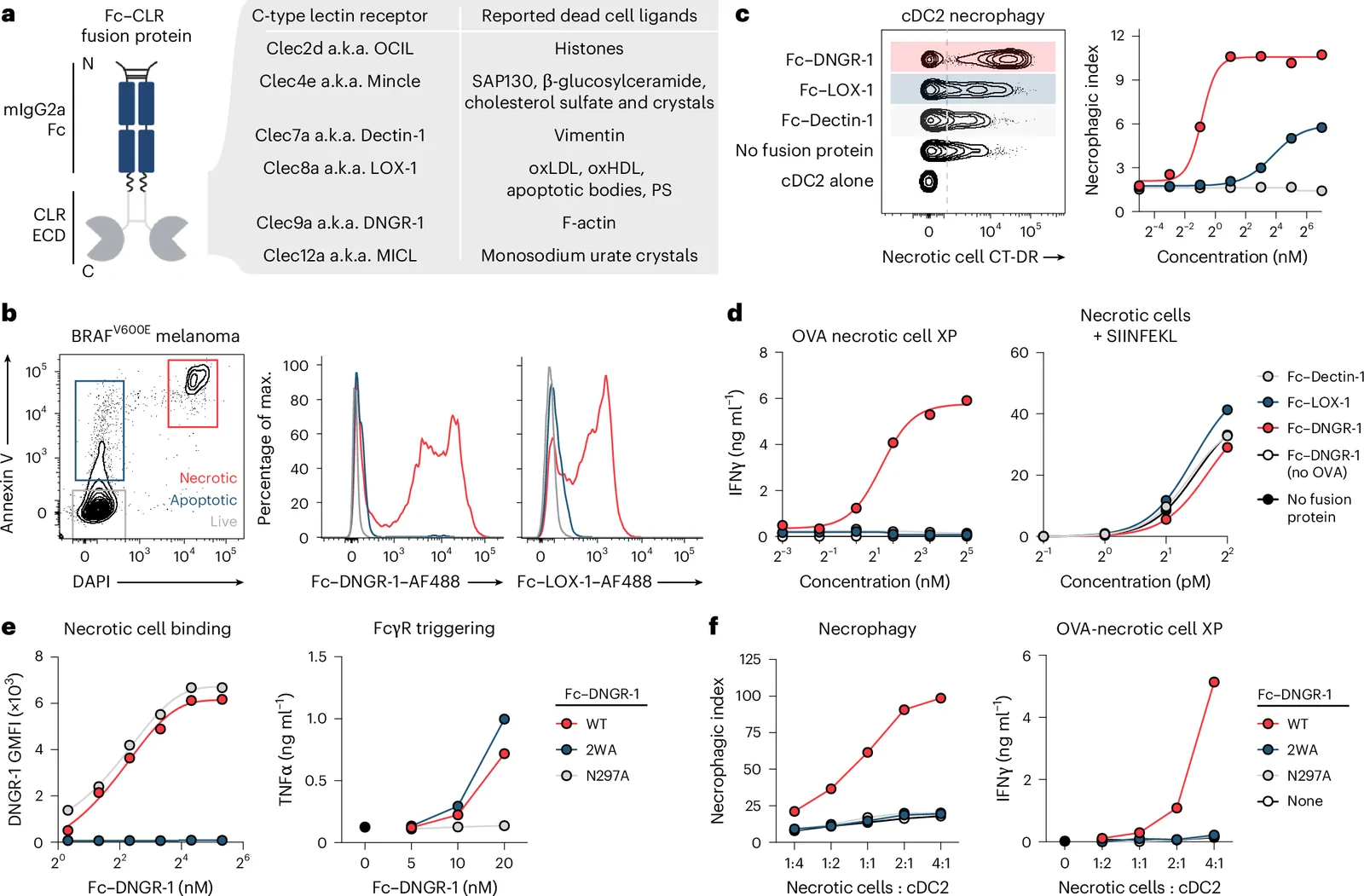
Fc–CLRs couple necrotic cell binding to necrophagy and XP. **a**, Schematic of Fc–CLR fusion proteins, showing both CLR nomenclature and common names, as well as reported dead cell ligands, adapted from ref. ^30^. a.k.a., also known as. **b**, Flow cytometric analysis of Fc–DNGR-1 and Fc–LOX-1 binding to live, apoptotic, or necrotic BRAF^V600E^ melanoma cells. **c**, Uptake of Cell Tracker-Deep Red (CT-DR)-labeled necrotic cell debris by Hoxb8 cDC2s with and without Fc–CLRs for 2 h, as assessed by flow cytometry. The mean from technical duplicates is plotted. **d**, ELISA for IFNγ release from OT-I CD8^+^ T cells cocultured with Hoxb8 cDC2s and OVA-necrotic cells with and without Fc–CLR fusion proteins (left). IFNγ production was compared to presentation of SIINFEKL peptide in the presence of Fc–CLRs and necrotic cells devoid of OVA (right). The mean from technical duplicates is plotted. **e**, Flow cytometric analysis of Fc–DNGR-1 variant binding to necrotic cells for 1 h (left) and ELISA for RAW264.7 cell cytokine production following FcγR triggering with plate-bound Fc–DNGR-1 variants (right). The mean from technical duplicates is plotted. **f**, Flow cytometric analysis of necrotic cell uptake by Hoxb8 cDC2s as in **c** (left) and ELISA for IFNγ release from OT-I CD8^+^ T cells cocultured with Hoxb8 cDC2s, OVA-necrotic cells and Fc–DNGR-1 variants as in **d** (right). The mean from technical duplicates is plotted. Data are representative of two (**b**) or three (**c**–**f**) biologically independent experiments. a.k.a., also known as; oxLDL, oxidized low-density lipoprotein; oxHDL, oxidized high-density lipoprotein; PS, phosphatidylserine. Schematic in **a** created in BioRender; Castro Dopico, T. https://biorender.com/pnx8l5h (2026).

**Extended Data Fig. 3:**
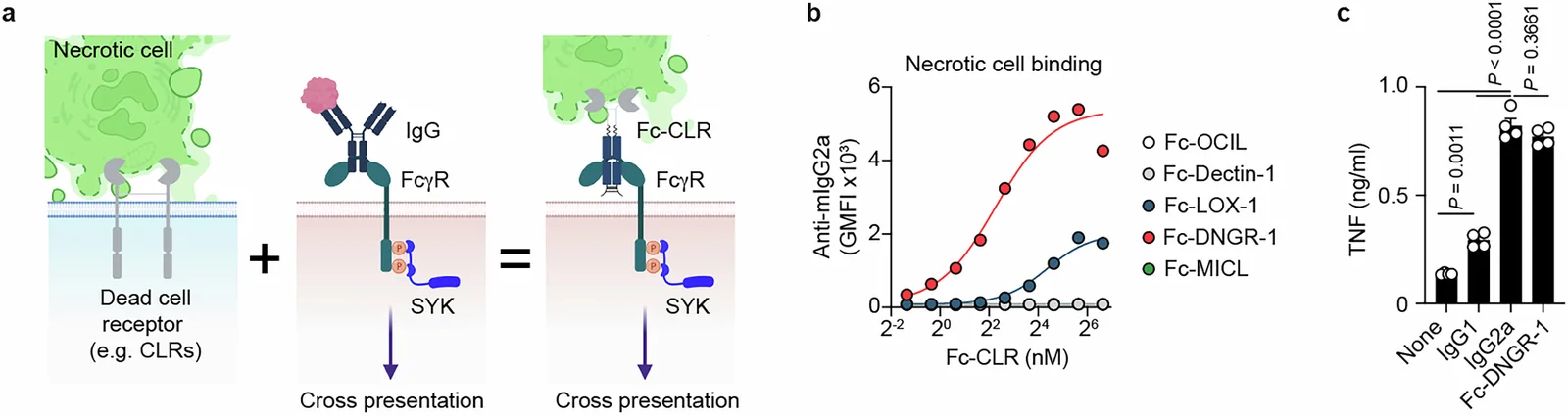
Fc-CLRs for coupling necrotic cell sensing to FcγRs. (**a**) Schematic of bridging necrotic cell sensing to FcγR signalling using Fc-CLRs. (**b**) Flow cytometric analysis of Fc-CLR binding to UV-irradiated necrotic Braf^V600E^ melanoma cells after 1 h, assessed by anti-mouse IgG2a detection. Single measurements are plotted. (**c**) Comparison of Fc-DNGR-1 to mouse IgG2a and IgG1 FcγR triggering in RAW264.7 cells. Mean ± s.e.m. from four technical measurements is plotted. Data are representative of three biologically independent experiments. Data were analysed using a one-way ANOVA. Schematic in **a** created in BioRender; Castro Dopico, T. https://biorender.com/h1w6v3l (2026).

Given these findings, we hypothesized that XP of tumor antigens could be induced in non-cDC1s by bridging recognition of necrotic cell debris to FcγRs (Extended Data Fig. 3a). Several members of the CLR family besides DNGR-1 have been reported to bind ligands exposed by dead cells^30^ (Fig. 2a). We designed six fusion proteins consisting of mouse IgG2a Fc (which has the highest affinity for activatory FcγRs among murine IgG subclasses) and the extracellular domain (ECD) of different CLRs, joined by a short flexible linker (Fig. 2a). As these CLRs are type II transmembrane proteins, mouse IgG2a Fc was fused to the CLR ECD via its carboxyl terminus, and the fusion proteins are termed Fc–CLRs.

Among the five of six Fc–CLRs that could be successfully expressed, only Fc–DNGR-1 and Fc–LOX-1 displayed appreciable binding to dead cells (Extended Data Fig. 3b), with Fc–DNGR-1 displaying the highest potency (Fc–DNGR-1 half-maximal effective concentration (EC_50_) = 4.82 nM versus Fc–LOX-1 EC_50_ = 20.57 nM) and efficacy (Fc–DNGR-1 maximum geometric mean fluorescence intensity (GMFI) = 5,392 versus Fc–LOX-1 maximum GMFI = 2,017). Notably, binding was specific to necrotic cells that had lost membrane integrity and was not observed with either live or apoptotic tumor cells (Fig. 2b). Despite the inverted Fc topology in Fc–CLRs compared to that of an antibody, Fc–DNGR-1 remained capable of cross-linking FcγR as effectively as mouse IgG2a (Extended Data Fig. 3c).

**Extended Data Fig. 4:**
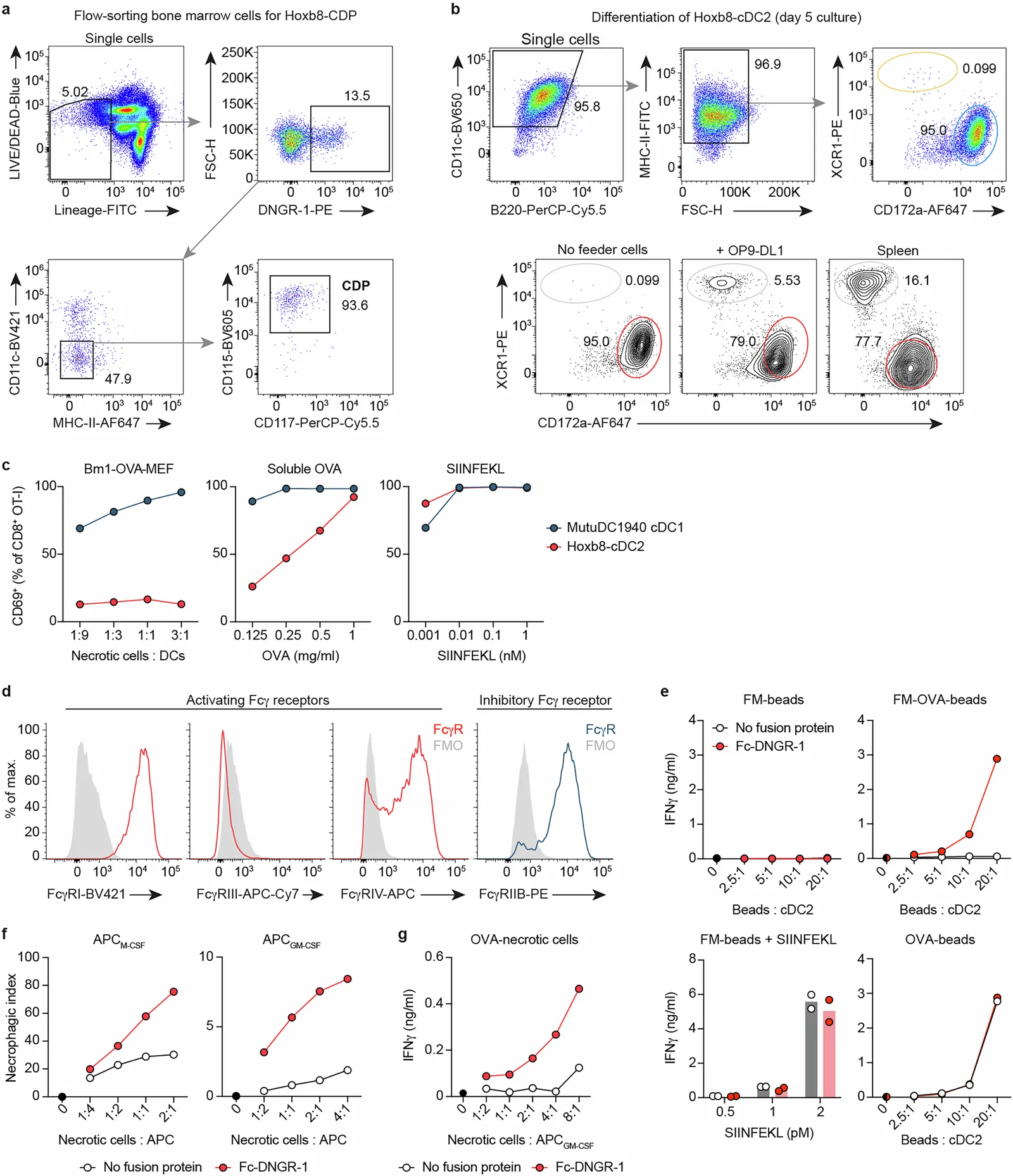
Fc-DNGR-1 promotes XP in Hoxb8-cDC2s. (**a**) Flow-sorting strategy for bone marrow CDP for generation of Hoxb8-immortalised progenitor cell line. (**b**) Flow cytometric analysis of Hoxb8-CDP differentiation profile in the presence of FLT3L ± OP9-DL1, compared to cDC populations in the spleen. (**c**) Flow cytometric analysis of CD69 upregulation by naïve OT-I CD8^+^ T cells co-cultured with Hoxb8-cDC2s or MutuDC1940 cDC1s and necrotic Bm1-OVA-MEF, soluble OVA, or OVA SIINFEKL peptide. Single measurements are plotted. (**d**) Flow cytometric analysis of FcγR expression by differentiated Hoxb8-cDC2s. Red = activating FcγR, blue = inhibitory FcγR. (**e**) ELISA for IFN-γ release from OT-I CD8^+^ T cells co-cultured with Hoxb8-cDC2 ± Fc-DNGR-1 and FM-, FM-OVA-, or OVA-beads. IFN-γ production was compared to presentation of SIINFEKL peptide in the presence FM-beads lacking OVA and Fc-DNGR-1. Mean from technical duplicates is plotted. (**f**) Flow cytometric analysis of necrotic cell uptake in primary bone marrow M-CSF- (APC_M-CSF_) and GM-CSF-derived (APC_GM-CSF_) cultures in the presence of Fc-DNGR-1 for 2 h. Mean from technical triplicates is plotted. (**g**) ELISA for IFN-γ release from OT-I CD8^+^ T cells co-cultured with APC_GM-CSF_ and OVA-pulsed necrotic cells ± Fc-DNGR-1. Mean from technical duplicates is plotted. Data are representative of three biologically independent experiments.

We next considered whether Fc–CLRs could induce necrotic cell uptake (here termed ‘necrophagy’) by cDC2s, which naturally lack necrophagic ability^13,14^. We generated an immortalized common DC progenitor (CDP) cell line using the Hoxb8 system^31^ (Extended Data Fig. 4a). Upon withdrawal of β-estradiol, Hoxb8 CDPs differentiated into cDC2s in the presence of FLT3 ligand (FLT3L) (Extended Data Fig. 4b). These cells were functionally comparable to bona fide cDC2s, poorly cross-presenting necrotic cell-associated antigens and soluble OVA compared to a cDC1 cell line (MutuDC1940) despite being able to present preprocessed OVA SIINFEKL peptide (Extended Data Fig. 4c). Notably, Hoxb8 cDC2s exhibited an FcγR expression profile akin to that of tumor cDC2s rather than spleen cDC2s, making them useful for study of FcγR-mediated XP (Extended Data Fig. 4d). As expected, Hoxb8 cDC2s exhibited low basal uptake of necrotic debris (Fig. 2c). However, necrophagy was markedly enhanced by Fc–DNGR-1 and to a lesser extent by Fc–LOX-1, but not by Fc–Dectin-1 (potency: Fc–DNGR-1 EC_50_ = 0.54 nM versus Fc–LOX-1 EC_50_ = 13.85 nM; efficacy: Fc–DNGR-1 maximum necrophagic index = 105.8 versus Fc–LOX-1 maximum necrophagic index = 59.3). Notably, Hoxb8 cDC2s did not effectively cross-present necrotic cell-associated OVA, but addition of Fc–DNGR-1 potently induced this activity, as measured by IFNγ release from OVA-specific effector CD8^+^ OT-I cells (Fig. 2d). No activity was observed with either Fc–LOX-1 or Fc–Dectin-1 or in the absence of OVA. Fc–CLRs did not alter the ability of Hoxb8 cDC2s incubated with necrotic cells and preprocessed SIINFEKL peptide to trigger CTL, ruling out indirect effects due to cell activation by FcγR triggering (Fig. 2d). Similarly, Fc–DNGR-1 enhanced XP of OVA-conjugated latex beads coated with DNGR-1 ligand (F-actin/myosin-II; FM) but did not improve presentation of exogenous SIINFEKL or XP of OVA beads lacking FM (Extended Data Fig. 4e). Augmentation of necrophagy and XP was also observed using primary macrophage/DC-like cells grown from mouse bone marrow in the presence of M-CSF or GM-CSF, which are known to express FcγRs (Extended Data Fig. 4f,g).

**Extended Data Fig. 5:**
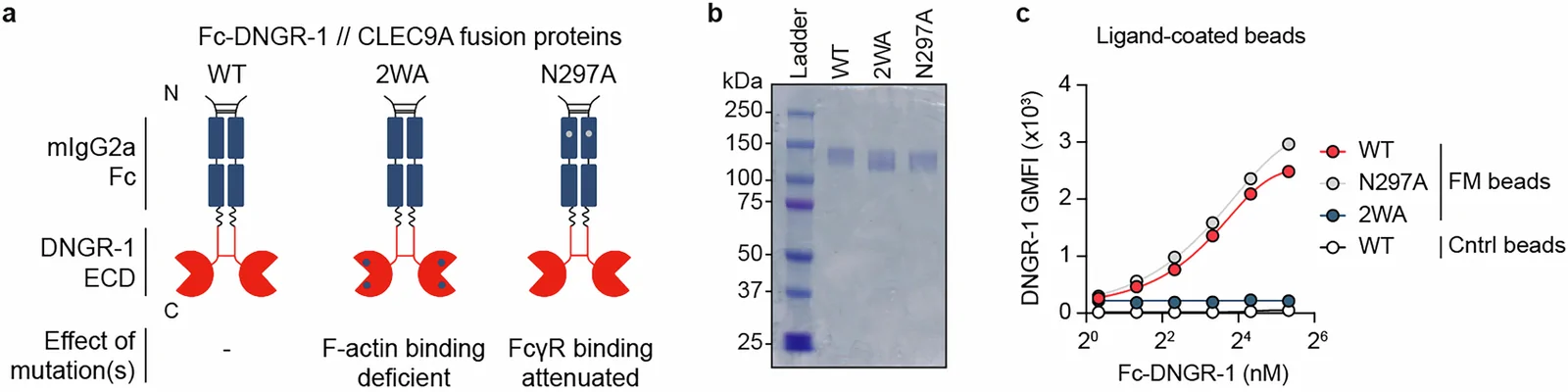
Validation of Fc-DNGR-1 mutant reagents. (**a**) Schematic of Fc-DNGR-1 fusion proteins. Mutations are represented by grey (N297A) or blue (2WA) circles. (**b**) Non-reducing SDS-PAGE of Fc-DNGR-1 fusion proteins. (**c**) Flow cytometric analysis of Fc-DNGR-1 fusion protein binding to 2 μm latex beads coated with F-actin/myosin-II complexes for 1 h. Mean from duplicate measurements is plotted. Data are representative of two (**b**) or three (**c**) biologically independent experiments. Schematic in **a** created in BioRender; Castro Dopico, T. https://biorender.com/ki7rob5 (2026).

Focusing on Fc–DNGR-1, we designed additional specificity controls: a mutant deficient in F-actin binding (2WA) and another with diminished binding to FcγRs (N297A) (Extended Data Fig. 5a). All Fc–DNGR-1 fusion proteins were expressed in mammalian cells with the expected molecular weight and minimal degradation (Extended Data Fig. 5b). Both wild-type (WT) and N297A Fc–DNGR-1 but not 2WA Fc–DNGR-1 were able to bind necrotic tumor cell debris (Fig. 2e) and FM beads (Extended Data Fig. 5c), confirming the F-actin specificity of the reagents. Plate-bound WT and 2WA Fc–DNGR-1 could cross-link FcγRs on RAW264.7 macrophages and trigger cytokine production, effects that were attenuated when N297A Fc–DNGR-1 was used (Fig. 2e). Notably, enhanced necrophagy and XP of dead cell-associated OVA by Hoxb8 cDC2s was abolished with either mutant form of Fc–DNGR-1 (Fig. 2f), confirming that Fc–DNGR-1 function requires bridging of F-actin recognition and FcγR triggering.

### Fc–DNGR-1 promotes necrophagy and XP by cDCs via FcγRI

**Fig. 3:**
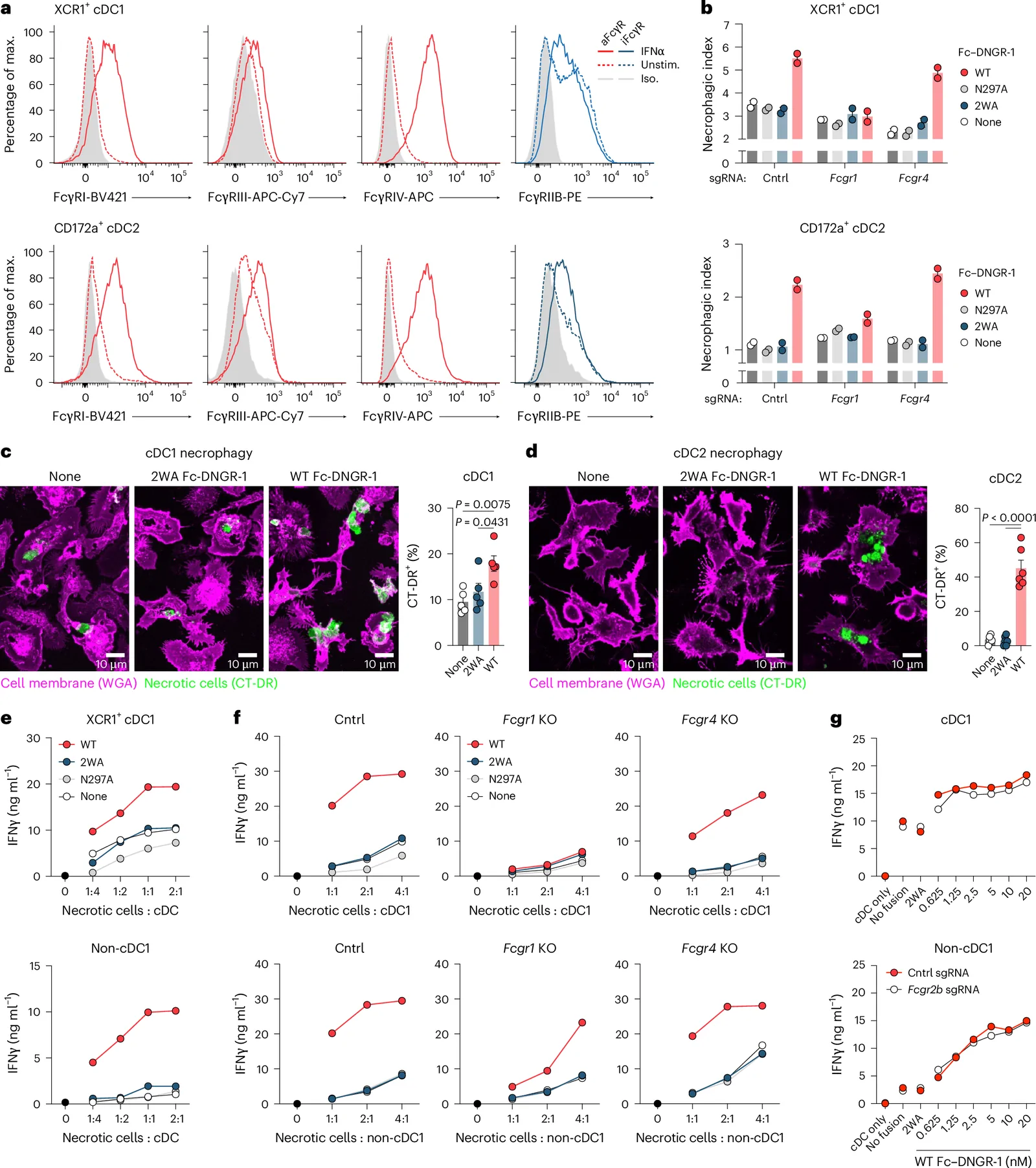
Fc–DNGR-1 promotes necrophagy and XP by cDCs via FcγRI. **a**, Flow cytometric analysis of FcγR expression by primary FLT3L cDC subsets treated for 24 h with IFNα. **b**, Uptake of CT-DR-labeled necrotic cells by primary CRISPR-edited FLT3L cDCs (control versus *Fcgr1* versus *Fcgr4*) with and without Fc–DNGR-1. Means from technical duplicates are shown. **c**,**d**, Confocal microscopy of purified primary FLT3L cDC1s (*n* = 5 fields of view) (**c**) and cDC2s (*n* = 6 fields of view) (**d**) cultured for 2 h as in **b**. Mean ± s.e.m. values are plotted. **e**, ELISA for IFNγ release from OT-I CD8^+^ T cells cocultured with primary FLT3L cDC1s or non-cDC1s and OVA-necrotic cells with and without Fc–DNGR-1 fusion proteins. Means from technical duplicates are plotted. **f**, ELISA for IFNγ release from OT-I CD8^+^ T cells cocultured with primary CRISPR-edited FLT3L cDC1s or non-cDC1s and OVA-necrotic cells with and without Fc–DNGR-1 fusion proteins. Means from technical duplicates is plotted. **g**, ELISA for IFNγ release from OT-I CD8^+^ T cells cocultured with cDC1s and non-cDC1s from primary CRISPR-edited FLT3L cDCs (control versus *Fcgr2b*) and OVA-soaked necrotic cells with and without Fc–DNGR-1. 2WA condition = 10 nM. Means from technical duplicates are plotted. Data are representative of two (**b**, **f** and **g**) or three (**a** and **c**–**e**) biologically independent experiments. Data in **c** and **d** were analyzed using one-way ANOVA. aFcγR, activating FcγR; Cntrl., control; iFcγR, inhibitory FcγR; iso., isotype; KO, knockout; unstim., unstimulated; WGA, wheat germ agglutinin.

**Extended Data Fig. 6:**
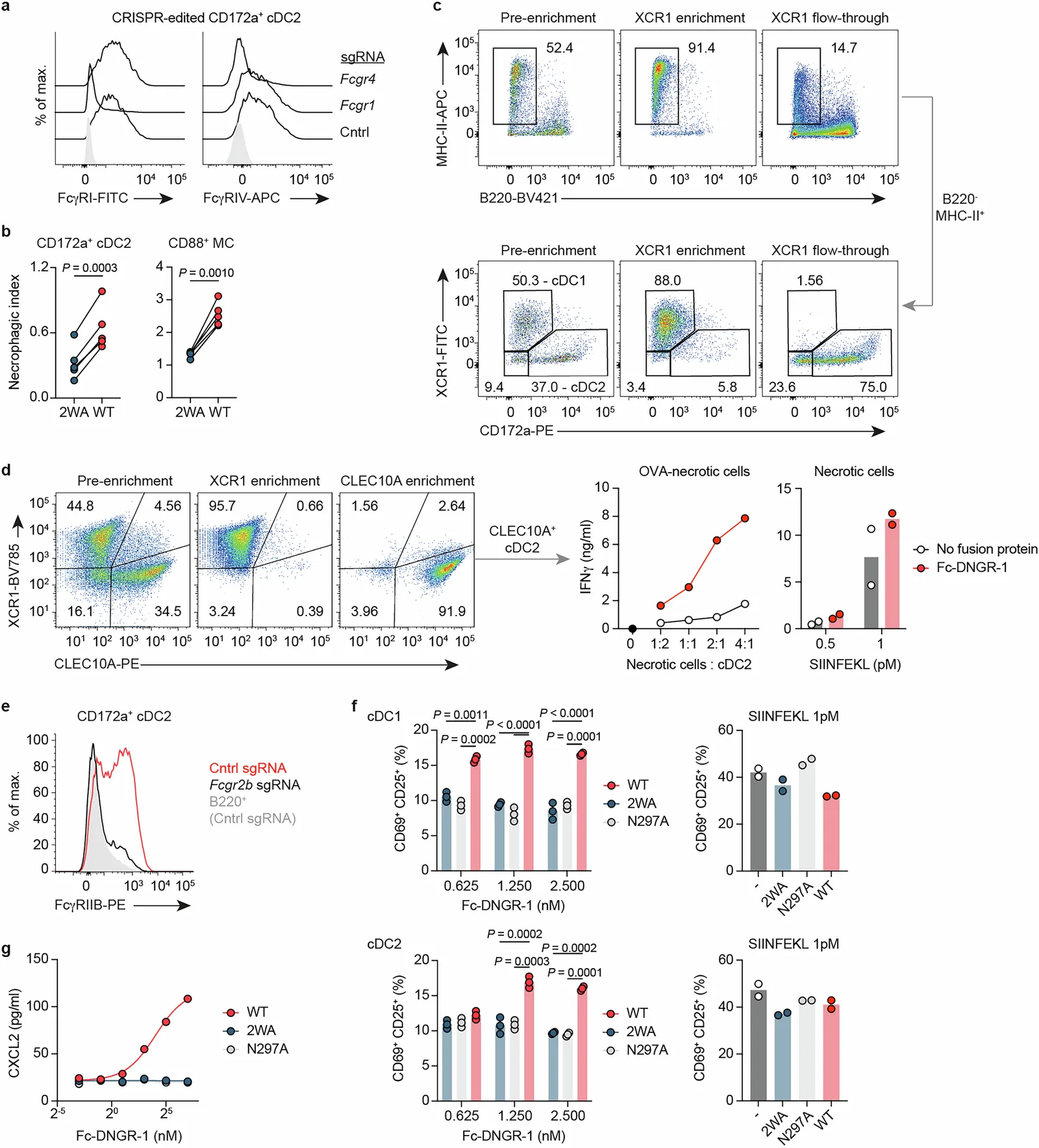
Fc-DNGR-1 promotes XP of necrotic cell-associated antigens by primary cDCs. (**a**) Flow cytometric analysis of efficacy of CRISPR-mediated KO of *Fcgr* genes in primary FLT3L cDCs. (**b**) Flow cytometric analysis of uptake of CT-DR-labelled necrotic cells by MCA205 tumour-derived APCs *ex vivo* (day 10 post implantation of 0.5 ×10^6^ cells) in the presence of WT or 2WA Fc-DNGR-1 (n = 5 mice per group). Cells isolated from single tumours were divided into 2 wells (2WA vs WT) and analysed via a paired analysis. (**c**) Flow cytometric analysis of enrichment process and purity of XCR1^+^ cDC1s and XCR1^*−*^ non-cDC1s from primary FLT3L-cDC cultures. (**d**) Alternative enrichment process for XCR1^+^ cDC1s and CLEC10A^+^ cDC2s from cultures in **c** (left), and ELISA for IFN-γ release from OT-I CD8^+^ T cells co-cultured with CLEC10A^+^ cDC2s and OVA-pulsed necrotic cells ± Fc-DNGR-1 (right). Non-OVA necrotic cells ± Fc-DNGR-1 and SIINFEKL were used to assess indirect effects of Fc-DNGR-1. Mean from technical duplicates is plotted. (**e**) Flow cytometric assessment of efficacy of *Fcgr2b* KO in CRISPR-edited primary cDC cultures (cDC2s shown). (**f**) Flow cytometric analysis of CD25 and CD69 upregulation by naïve OT-I CD8^+^ T cells co-cultured with primary flow-sorted FLT3L-cDC1s and cDC2s, OVA-necrotic cells, and Fc-DNGR-1. Mean ± s.e.m. from technical triplicates (left) or mean from technical duplicates (right) is plotted. (**g**) ELISA measurement of CXCL2 in supernatants from Hoxb8-cDC2s cultured with necrotic tumour cells (4:1) and Fc-DNGR-1 overnight. Mean from technical duplicates is plotted. Data are representative of two (**a**, **b**, **d**-**f**) or three (**c**, **g**) biologically independent experiments. Data were analysed using a two-tailed paired *t* test (**b**) or Tukey-corrected two-way ANOVA (**f**).

Next, we tested the effects of Fc–DNGR-1 on primary cDCs grown from bone marrow using FLT3L (FLT3L cDCs). Like splenic cDCs, FLT3L cDCs expressed only low levels of FcγRs, which could be upregulated to the extent seen on tumor cDCs or Hoxb8 cDC2s by exposing the cells to IFNα (Fig. 3a). As expected, cDC1s from IFNα-primed cultures efficiently internalized necrotic cell debris compared to cDC2s, which displayed only basal necrophagic activity (Fig. 3b). However, necrophagy was markedly augmented in both cDC1s and cDC2s by treatment with WT Fc–DNGR-1, but not mutant 2WA or N297A Fc–DNGR-1. Although several studies have reported a role for FcγRs in the uptake and XP of OVA-ICs^21,22,32–40^, the contribution of specific FcγRs to these processes has remained largely unknown. CRISPR–Cas9-mediated deletion of *Fcgr* genes demonstrated that FcγRI was important for Fc–DNGR-1 enhancement of necrophagy in both cDC1s and cDC2s (Fig. 3b and Extended Data Fig. 6a). Using confocal microscopy, we observed that WT Fc–DNGR-1 but not 2WA Fc–DNGR-1 promoted the internalization of several necrotic cell corpses per primary cDC2, as well as boosting the scavenging of small dead cell debris by cDC1s (Fig. 3c,d and Supplementary Videos 1 and 2), although the latter was more difficult to detect by microscopy than by flow cytometry (compare Fig. 3b,d). Finally, we confirmed that WT but not 2WA Fc–DNGR-1 could markedly potentiate necrophagy by CD172a^+^ cDC2s and CD88^+^ MCs directly isolated from tumors (Extended Data Fig. 6b).

Next, we compared XP of dead-cell-associated antigen by XCR1^+^ cDC1s versus non-cDC1s (primarily cDC2s and plasmacytoid cells) purified from primary FLT3L cDC cultures (Extended Data Fig. 6c). As expected, cDC1s effectively cross-presented OVA-coated necrotic cells, and this activity was further enhanced by WT but not 2WA or N297A Fc–DNGR-1 (Fig. 3e). By contrast, non-cDC1s did not effectively cross-present necrotic cell-associated OVA, but this activity was markedly promoted by WT Fc–DNGR-1, to the point that it became equivalent to that of cDC1s (Fig. 3e). Similar results were obtained with pure CLEC10A^+^ cDC2s isolated from the non-cDC1 fraction (Extended Data Fig. 6d). Finally, in agreement with the necrophagy data, Fc–DNGR-1-mediated XP was markedly attenuated in both cDC subsets by CRISPR–Cas9-mediated knockout of *Fcgr1* but not *Fcgr4*, although residual XP in FcγRI knockout suggests a role for additional FcγRs, especially in non-cDC1s (Fig. 3f). FcγRIIB ablation had little impact (Fig. 3g and Extended Data Fig. 6e), consistent with the relatively low levels of FcγRIIB on FLT3L cDCs and preferential engagement of activating FcγRs by mouse IgG2a Fc^41^.

In addition to XP to effector CD8^+^ T cells, Fc–DNGR-1 enhanced cross-priming of naive CD8^+^ T cells by both FLT3L cDC1s and non-cDC1s in response to OVA-coated dead cells but did not affect priming in response to SIINFEKL in the presence of necrotic cells lacking OVA (Extended Data Fig. 6f). Finally, we explored the ability of Fc–DNGR-1 to promote FcγR-driven myeloid cell activation and observed a boost in cytokine production from HOXB8 cDC2s treated with necrotic cells and WT Fc–DNGR-1 but not mutant reagents (Extended Data Fig. 6g). In summary, these findings demonstrate that necrophagy and XP of dead cell-associated antigens, as well as activation, can be potently induced in a variety of APCs as well as cDC1s by bridging F-actin to FcγRs.

### FcγR signaling triggers phagosomal damage

**Fig. 4:**
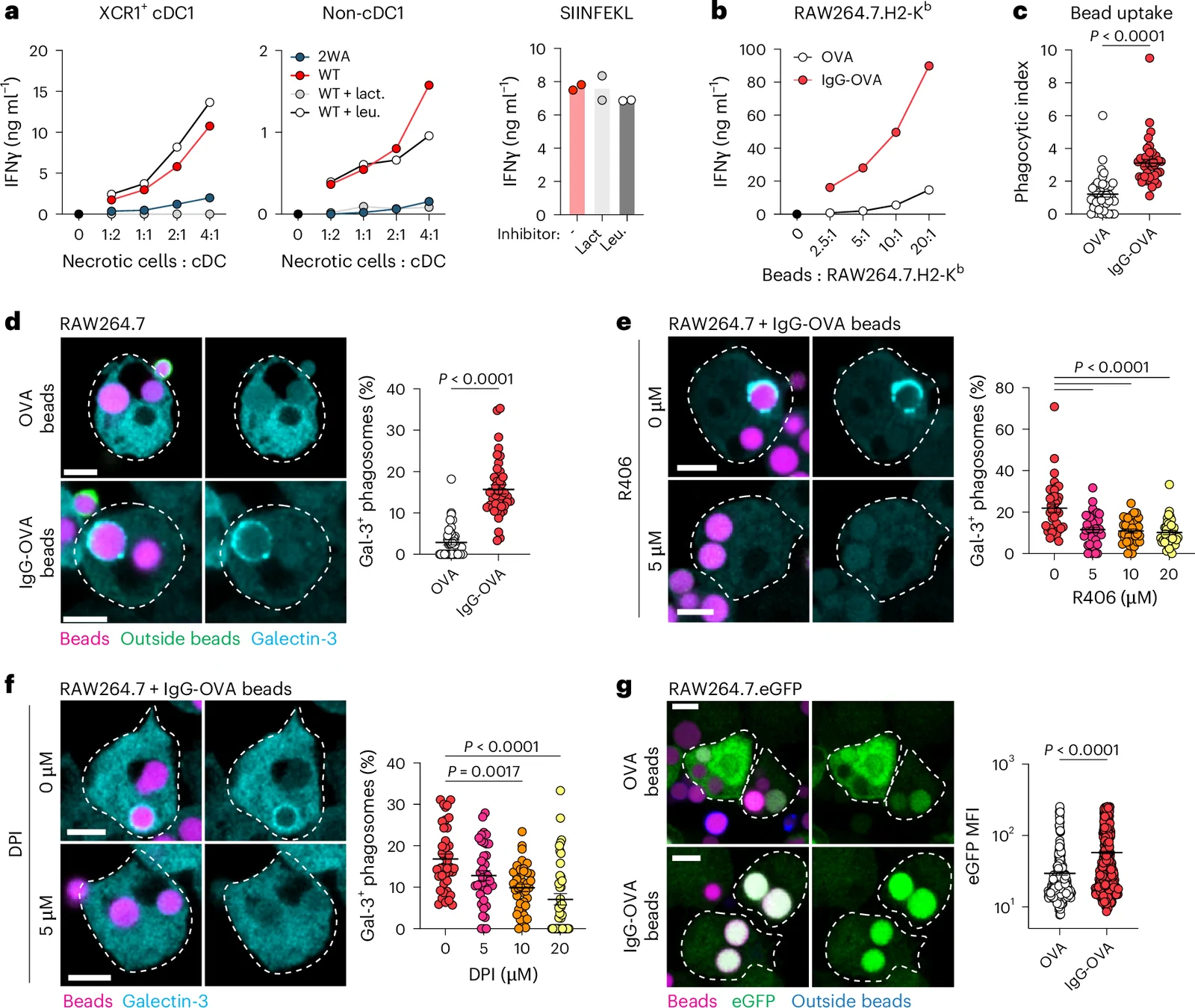
FcγR signaling triggers phagosomal damage. **a**, ELISA for IFNγ release from OT-I CD8^+^ T cells cocultured with primary FLT3L cDC subsets, OVA-necrotic cells, and WT or 2WA Fc–DNGR-1 in the presence of proteasome inhibitor lactacystin (gray) or protease inhibitor leupeptin (white). Presentation of preprocessed OVA SIINFEKL peptide was assessed in the presence of inhibitors. The mean from technical duplicates is plotted. **b**, ELISA for IFNγ release from OT-I CD8^+^ T cells cocultured with RAW264.7.H2-K^b^ cells and IgG-OVA or control OVA beads. The mean from technical duplicates is plotted. **c**, Confocal microscopy analysis of uptake of OVA and IgG-OVA beads by RAW264.7 cells after 3 h. The mean ± s.e.m. from 40 measurements is plotted. **d**, Confocal microscopy of galectin-3 accumulation on IgG-OVA versus control OVA bead phagosomes in RAW264.7 cells after 3 h. The mean ± s.e.m. from 40 measurements is plotted. Scale bars: 5 μm. **e**,**f**, Galectin-3^+^ accumulation as in **d** in the presence of SYK inhibitor R406 (**e**) or NADPH oxidase inhibitor DPI (**f**). The mean ± s.e.m. from 40 measurements is plotted. Scale bars: 5 μm. **g**, Confocal microscopy analysis of eGFP influx into IgG-OVA and OVA bead phagosomes in RAW264.7.eGFP cells after 3 h. The mean ± s.e.m. from 40 measurements is plotted. Scale bars: 5 μm. Data are representative of two (**a** and **d**–**g**) or three (**b** and **c**) biologically independent experiments. Data were analyzed using two-tailed Kolmogorov–Smirnov test (**c**, **d** and **g**) or one-way ANOVA (**e** and **f**). DPI, diphenyleneiodonium; Lact., lactacystin; Leu., leupeptin.

Both P2C and endosomal processing have been implicated in FcγR-mediated XP of antigen ICs^22,23^. Consistent with a role for P2C, Fc–DNGR-1-mediated necrotic cell XP by cDCs was inhibited by proteasome inhibitor lactacystin but not by endosomal protease inhibitor leupeptin (Fig. 4a). Neither inhibitor affected the ability of cDCs to present exogenous SIINFEKL peptide. To more directly implicate P2C, we assessed whether FcγR signaling could, like DNGR-1, promote phagosomal damage. For this, we utilized RAW264.7 macrophages, a genetically tractable FcγR-expressing cell line that has been used previously to investigate the role of P2C in XP^16,42^. RAW264.7 macrophages were fed with opsonized or unopsonized silica beads for easy identification of phagosomes. First, we confirmed that RAW264.7.H2-K^b^ cells could cross-present IgG-coated OVA beads more efficiently than control OVA beads (Fig. 4b). Next, we monitored recruitment of galectin-3 to bead phagosomes as a marker of phagosomal membrane damage. After normalization for increased uptake (Fig. 4c), a larger proportion of IgG bead than control bead phagosomes exhibited galectin-3 accumulation (Fig. 4d). This galectin-3 recruitment was diminished by either SYK or NADPH oxidase inhibitors (Fig. 4e,f). As an alternative approach, we assessed phagosomal accumulation of fluorescence in RAW264.7 cells expressing cytosolic eGFP. In line with increased galectin-3 staining, IgG-OVA bead phagosomes exhibited enhanced eGFP signals compared to OVA bead phagosomes (Fig. 4g). Collectively, these findings suggest that FcγR signaling via SYK and NADPH oxidase can lead to phagosomal membrane damage and allow P2C, indicating a cellular mechanism for FcγR-mediated XP akin to that described for DNGR-1.

### FcγR^+^ APCs localize in proximity to necrotic cells in tumors

**Fig. 5:**
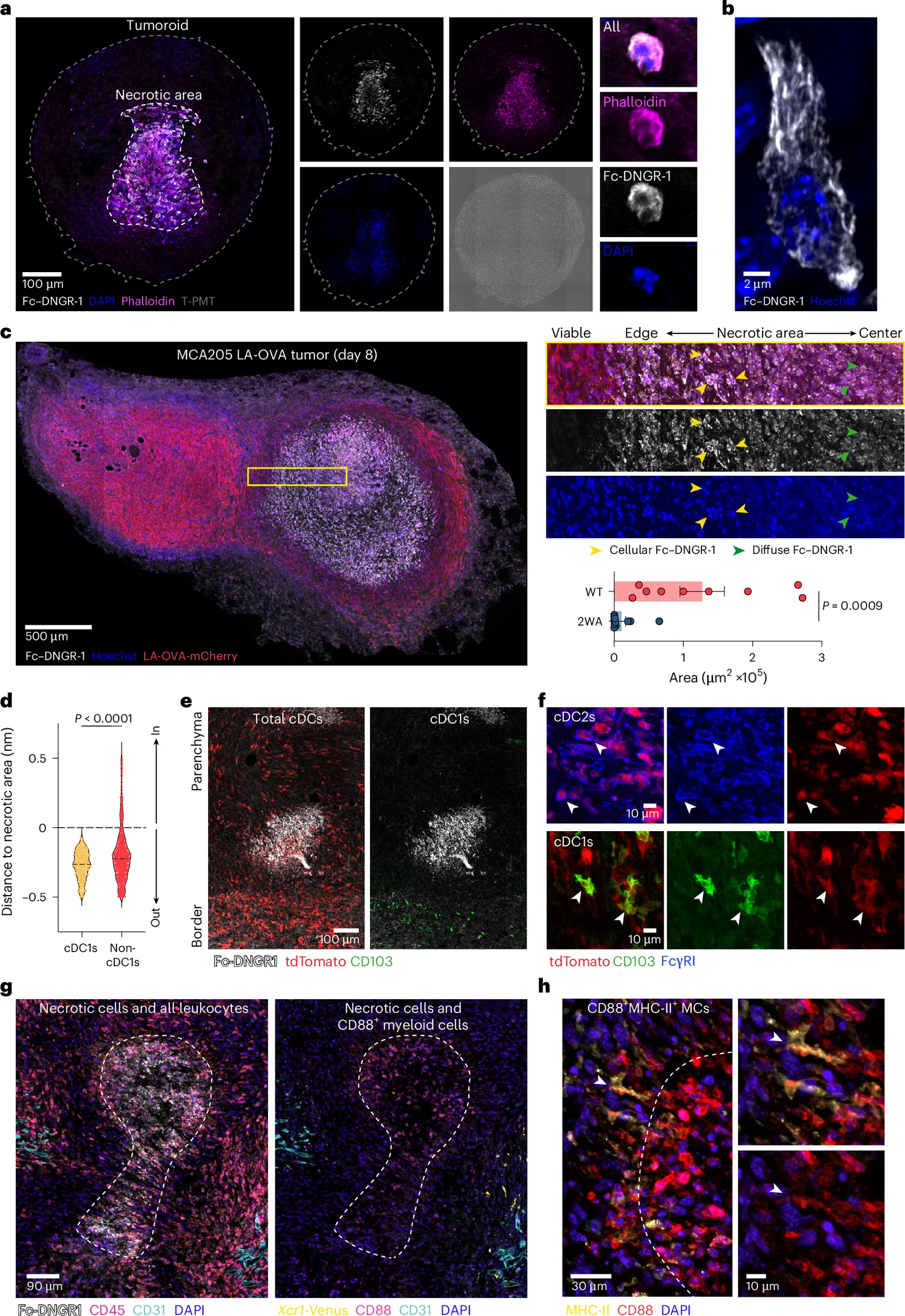
FcγR^+^ APCs localize in proximity to necrotic cells in tumors. **a**, Confocal microscopy of BRAF^V600E^ melanoma tumoroids cultured in vitro with intracellular binding reagents. **b**, Example filamentous staining pattern of Fc–DNGR-1 on necrotic cells within tumoroids, prepared as in **a**. **c**, Confocal microscopy of murine MCA205-LifeAct (LA)-OVA-mCherry tumors (day 8 postimplantation into C57BL/6 mice) after injection with WT or 2WA Fc–DNGR-1. The image shown was obtained following a 200-μg dose injected s.c. Quantification was performed on *n* = 9 independent tumors after 50 μg injected s.c. Data were pooled from three experiments, and the mean ± s.e.m. is plotted. **d**, Confocal microscopy distribution analysis of MHC-II^+^ APC populations (CD103^+^ cDC1s versus CD103^−^ non-cDC1s) in MCA205-LA-OVA-mCherry tumors relative to necrotic area defined by Fc–DNGR-1. Each dot represents a single cell (*n* = 262 cDC1s and *n* = 765 non-cDC1s) within a single tumor, and only APCs <500 μm from the necrotic core were analyzed. Median values are plotted. **e**, Confocal microscopy of MCA205-LA-OVA-mCherry tumors treated as in **c** (200 μg s.c. Fc–DNGR-1) from *Clec9a*^*Cre*^*Rosa*^*LSLtdTomato*^ mice to identify cDC1s (tdTomato^+^ CD103^+^) and cDC2s (tdTomato^+^ CD103^−^). **f**, Assessment of CD103 and FcγRI on lineage-traced populations from **e**. **g**,**h**, High-dimensional analysis of MCA205-LA-OVA-mCherry tumors from *Xcr1*^*Venus*^ mice treated as in **e** to determine spatial localization of CD88^+^ myeloid cells (**g**) and CD88^+^ MHC-II^+^ MCs (**h**). Data are representative of two (**d**–**h**), three (**a** and **b**) or four (**c**) biologically independent experiments. Data in **c** and **d** were analyzed using two-tailed unpaired Student’s *t*-test.

**Extended Data Fig. 7:**
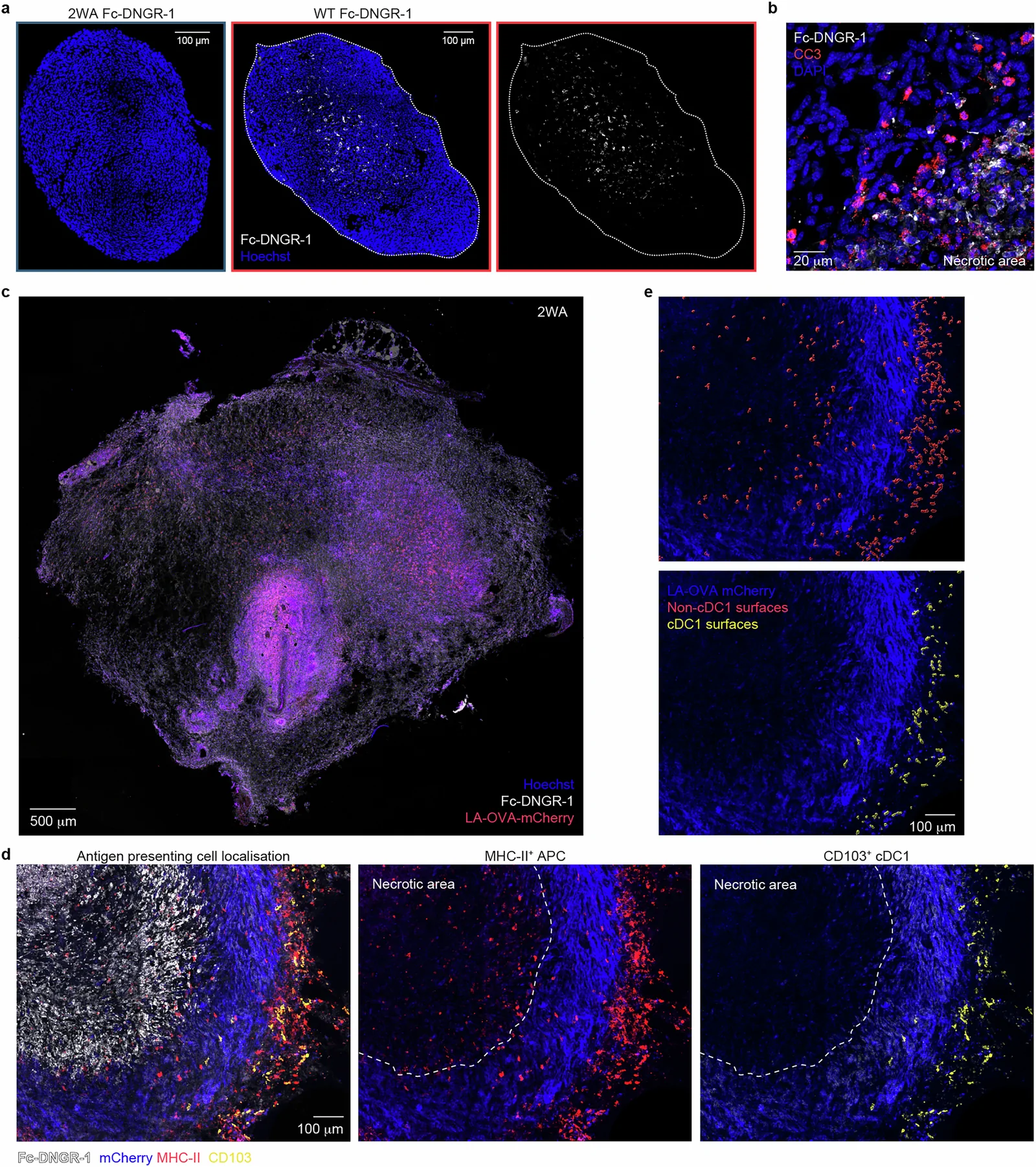
FcγR^+^ APCs localise in proximity to necrotic cells in tumours. (**a**) Confocal microscopy of 20 μm sections from Braf^V600E^ melanoma tumouroids cultured with WT or 2WA Fc-DNGR-1 overnight. (**b**) Co-localisation of Fc-DNGR-1 and cleaved caspase-3 (CC3) staining on tumouroids from **a**. (**c**) Confocal microscopy of MCA205 LA-OVA-mCherry tumours (day 8 post-implantation of 2.5 ×10^6^ cells) 24 h after injection with 2WA Fc-DNGR-1 (200 μg s.c.). (**d**) Confocal microscopy analysis of MHC-II^+^ APC populations (CD103^+^ cDC1s vs CD103^*−*^ non-cDC1s) in tumours relative to necrotic area defined by Fc-DNGR-1. (**e**) Imaris-calculated surfaces of non-cDC1s (MHC-II^+^ CD103^*−*^) and cDC1s (CD103^+^ MHC-II^+^) in tumours from **c** used to determine proximity to necrotic core, as determined by Fc-DNGR-1 staining. Data are representative of three (**a**, **b**, **d**, **e**) or four (**c**) biologically independent experiments.

To assess the spatiotemporal dynamics of necrotic cell sensing by immune cells in tumors, we used Fc–DNGR-1 as an imaging tool. In vitro incubation of tumor spheroids (tumoroids) with WT Fc–DNGR-1 but not 2WA Fc–DNGR-1 highlighted cells in the tumoroid core (Extended Data Fig. 7a), an area with probable restricted nutrient exchange and notable cleaved caspase-3 staining (Extended Data Fig. 7b). Fc–DNGR-1 staining reflected general loss of tumor cell membrane integrity, as necrotic cells could also be visualized by culturing tumoroids with other reagents that bind F-actin (such as phalloidin) or DNA (such as DAPI) (Fig. 5a). Consistent with recognition of the actin cytoskeleton, several necrotic cells exhibited discrete filament-like Fc–DNGR-1 staining patterns (Fig. 5b).

To identify areas of tumor necrosis in vivo, we administered Fc–DNGR-1 to mice bearing an MCA205-derived tumor modified to express a fluorescent marker, mCherry^43^. To minimize disruption of tumor architecture and preserve tissue for microscopy, Fc–DNGR-1 was injected peritumorally rather than intratumorally (i.t.). Consistent with the tumoroid data, WT Fc–DNGR-1 effectively labeled necrotic cells within the tumor center (Fig. 5c). No staining was observed with 2WA Fc–DNGR-1, confirming specificity (Fig. 5c and Extended Data Fig. 7c). Bright Fc–DNGR-1^+^ cells were observed at the border of the necrotic zone, with more diffuse Fc–DNGR-1 staining within the center of the necrotic area, perhaps reflecting the disintegration of cells that underwent necrosis early. We stained tumor sections for MHC-II and CD103 to identify CD103^+^ cDC1s and CD103^−^ non-cDC1 APCs (for example, cDC2s, MCs) (Extended Data Fig. 7d,e). Remarkably, cDC1s were located toward the tumor periphery away from Fc–DNGR-1^+^ necrotic areas. By contrast, MHC-II^+^ CD103^−^ APCs were located throughout the tumor, including at the boundary of and within the necrotic area (Fig. 5d and Extended Data Fig. 7d,e).

To more accurately assess the localization of cDC2s and MCs, we utilized two approaches. First, we repeated the experiments in *Clec9a*^*Cre*^*Rosa*^*LSLtdTomato*^ mice in which the cDC lineage is traced with tdTomato^44^. By costaining with CD103, we confirmed that CD103^+^ tdTomato^+^ cDC1s were located distally to Fc–DNGR-1^+^ necrotic areas, whereas abundant CD103^−^ tdTomato^+^ cDC2s expressing FcγRI were located throughout the parenchyma and into the necrotic area (Fig. 5e,f). Second, we performed high-dimensional microscopy through iterative staining of MCA205-LA-OVA-mCherry tumors implanted in *Xcr1*^*Venus*^ mice, in which cDC1s express Venus fluorescent protein^45^. XCR1^+^ cDC1s were localized close to vasculature away from Fc–DNGR-1-labeled necrotic areas, which were located distally to CD31^+^ vessels (Fig. 5g). Strikingly, necrotic areas were heavily infiltrated by CD45^+^CD88^+^ cells (including many expressing MHC-II), which were much rarer in the surrounding parenchyma (Fig. 5g,h). These data suggest that FcγR^+^ cDC2s and MCs infiltrate areas of tumor necrosis to a greater extent than cDC1s and are thus optimally positioned to acquire necrotic cell antigens in vivo in the presence of agents that bridge F-actin to FcγRs.

### Fc–DNGR-1 promotes FcγR-dependent tumor control

**Fig. 6:**
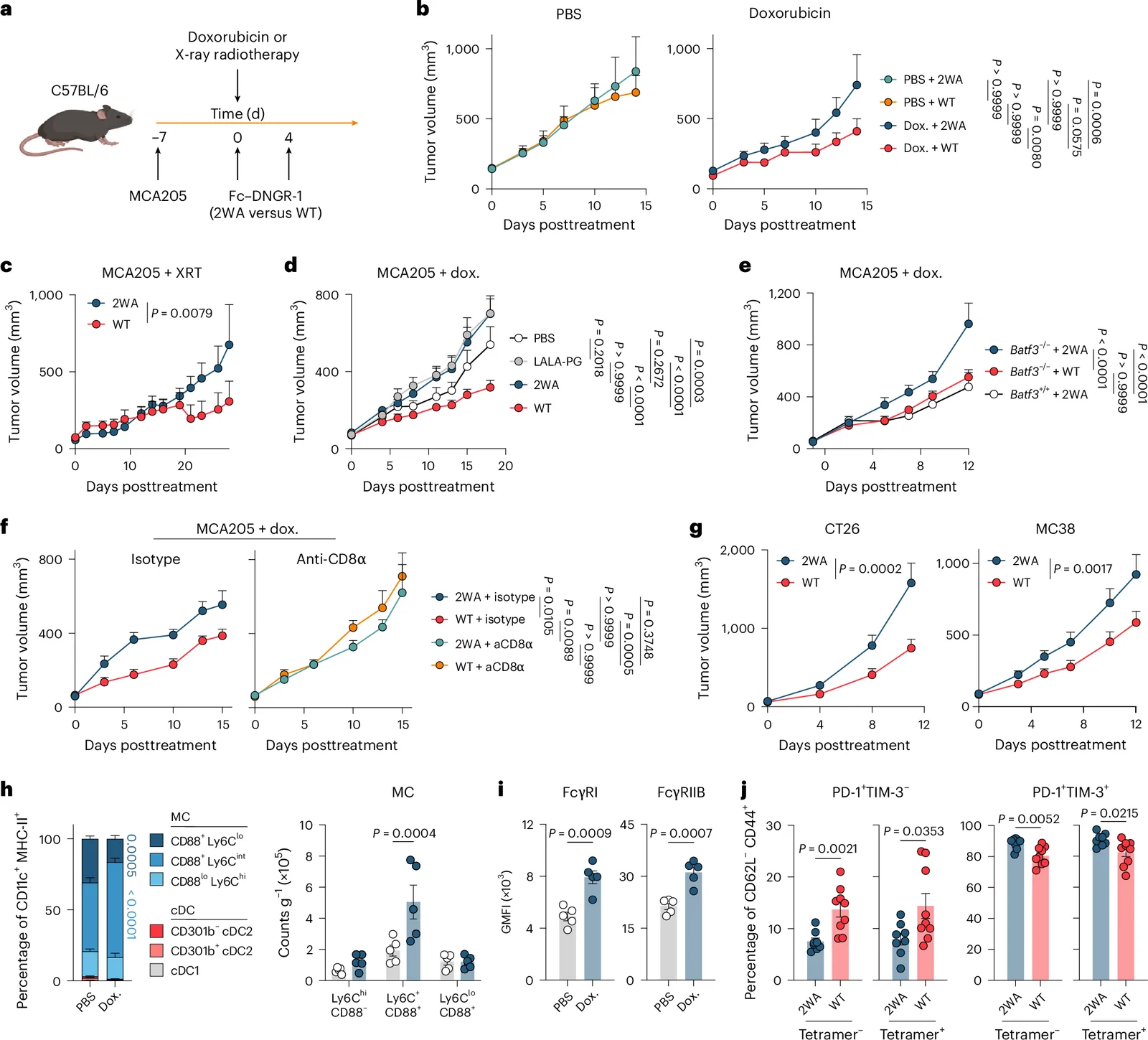
Fc–DNGR-1 promotes FcγR-dependent tumor control. **a**, Schematic of MCA205 experiments in C57BL/6 mice with cell-death-inducing therapies and Fc–DNGR-1. **b**,**c**, Tumor growth profiles from C57BL/6 mice implanted with 0.5 × 10^6^ MCA205 tumor cells and treated with WT or 2WA Fc–DNGR-1 (i.t. 50 μg, days 7 and 11 postimplantation) in combination with PBS or doxorubicin (i.t. 2.5 mg kg^−1^, day 7) (**b**) or 10 Gy localized X-ray radiotherapy (day 7) (**c**). Mice per group for **b**: 2WA + PBS, *n* = 9; WT + PBS, *n* = 9; 2WA + doxorubicin, *n* = 10; WT + doxorubicin, *n* = 10. Mice per group for **c**: 2WA + XRT, *n* = 12; WT + XRT, *n* = 11. The mean ± s.e.m. is plotted. **d**, Tumor growth profiles for MCA205-bearing mice treated with doxorubicin as in **b** and additional treatment with PBS or LALA-PG Fc–DNGR-1 (200 μg i.t.). Mice per group: PBS, *n* = 9; LALA-PG, *n* = 9; 2WA, *n* = 8; WT, *n* = 9. The mean ± s.e.m. is plotted. **e**, Tumor growth profiles for MCA205-bearing *Batf3*^*−/−*^ and *Batf3*^*+/+*^ mice treated as in **d** (*n* = 9 mice per group). The mean ± s.e.m. is plotted. **f**, Tumor growth profiles for MCA205-bearing mice treated as in **d** and further treated with isotype or anti-CD8α IgG (300 μg i.p.). Mice per group: 2WA + isotype, *n* = 8; WT + isotype, *n* = 7; 2WA + anti-CD8α IgG, *n* = 8; WT + anti-CD8α IgG, *n* = 10. The mean ± s.e.m. is plotted. **g**, Tumor growth profiles for BALB/c (left) and C57BL/6 (right) mice implanted with 0.5 × 10^6^ CT26 or MC38 tumor cells, respectively, and treated with WT or 2WA Fc–DNGR-1 (i.t. 200 μg, days 7 and 11 postimplantation). Mice per group: CT26 2WA, *n* = 6; CT26 WT; *n* = 7; MC38 2WA, *n* = 10; MC38 WT, *n* = 8. The mean ± s.e.m. is plotted. **h**, Flow cytometric assessment of tumor-infiltrating APC frequency (left) and absolute numbers (right) from MCA205-bearing mice 3 days posttreatment with PBS or doxorubicin (*n* = 5 per group). The mean ± s.e.m. is plotted. **i**, Flow cytometric analysis of FcγR expression in tumor CD88^+^Ly6C^int^ MCs from mice in **h**. The mean ± s.e.m. is plotted. **j**, Flow cytometric analysis of antigen-specific CD8^+^ T cell responses in MCA205-LA-OVA-bearing mice treated as in **d** at 7 days posttreatment initiation. Mice per group: 2WA, *n* = 8; WT, *n* = 9. The mean ± s.e.m. is plotted. Data are representative of two (**c**–**f** and **h**–**j**) or three (**b** and **g**) biologically independent experiments. Data were analyzed using Bonferroni-corrected two-way ANOVA (**b**–**h**), or two-tailed Student’s *t*-test (**i** and **j**). Dox., doxorubicin; XRT, X-ray radiotherapy. Illustration in **a** created in BioRender; Castro Dopico, T. https://biorender.com/a1ndihk (2026).

**Extended Data Fig. 8:**
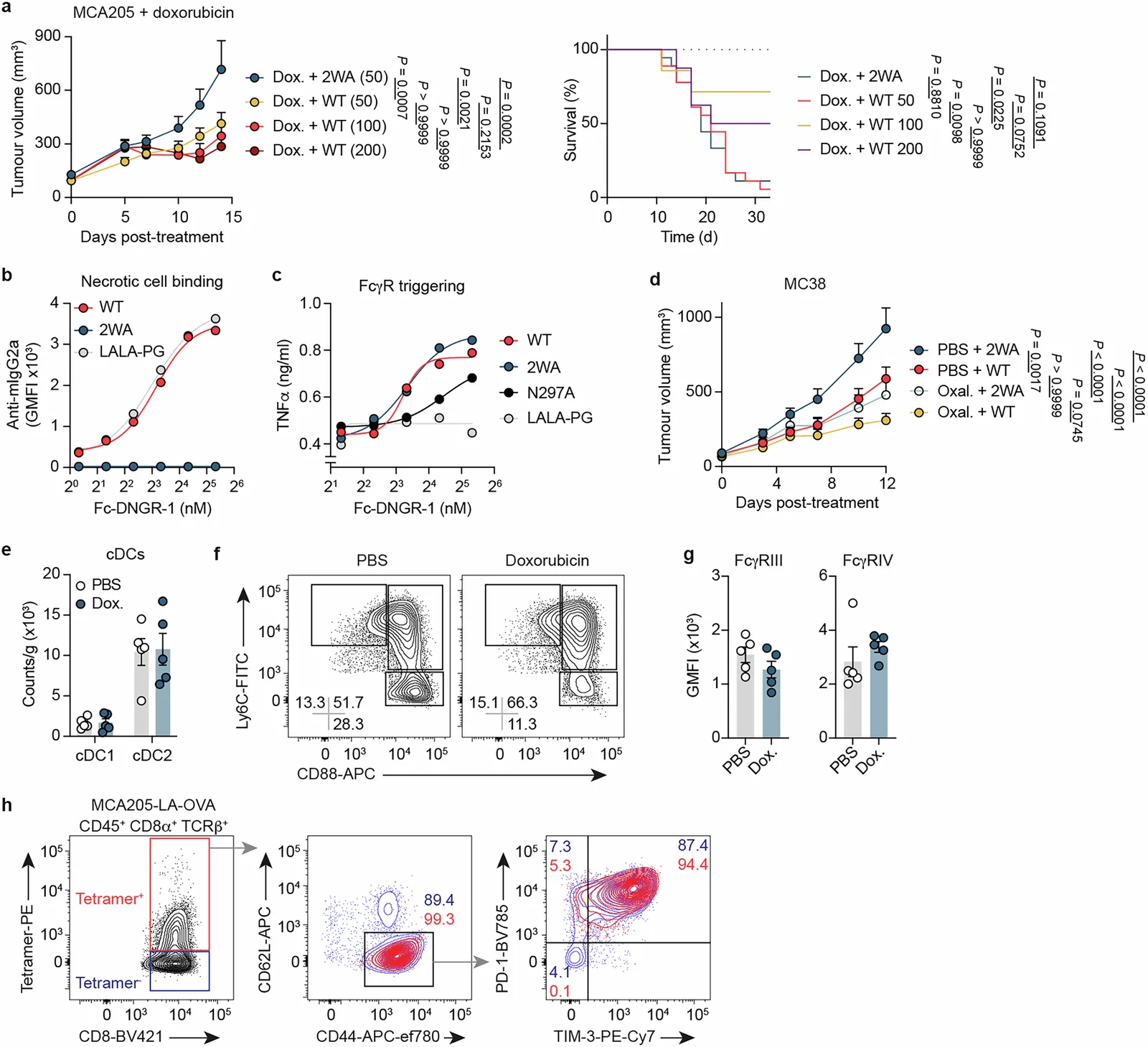
Fc-DNGR-1 promotes FcγR-dependent tumour control. (**a**) Tumour growth curves and overall survival for dose escalation of Fc-DNGR-1 (i.t. 50, 100, 200 μg) in MCA205 tumours treated with doxorubicin (i.t. 2.5 mg/kg, day 7). Data pooled from two independent experiments, including 50 μg 2WA (n = 10) and WT (n = 10) groups from Fig. 6b. Final mice per group: 50 μg 2WA (n = 18), 50 μg WT (n = 18), 100 μg WT (n = 7), 200 μg WT (n = 8). Mean ± s.e.m. is plotted. (**b**) Flow cytometric analysis of Fc-DNGR-1 variant binding to necrotic cells for 1 h. Mean from technical duplicates is plotted. (**c**) ELISA for RAW264.7 cell cytokine production following FcγR triggering with plate-bound Fc-DNGR-1 variants. Mean from technical duplicates is plotted. (**d**) Tumour growth profiles in C57BL/6 mice implanted with MC38 tumours and treated with 2WA or WT Fc-DNGR-1 (i.t. 200 μg, day 7, 11 and 14) and vehicle or oxaliplatin (i.t. 1.25 mg/kg, day 7 and 14). Mice per group: 2WA + vehicle (n = 10), WT + vehicle (n = 8), 2WA + oxaliplatin (n = 10), WT + oxaliplatin (n = 10). Mean ± s.e.m. is plotted. Vehicle groups are reproduced from Fig. 6g. (**e**-**g**) Flow cytometric assessment of cDC numbers (**e**), MC frequency (**f**), and FcγR expression in CD88^+^ Ly6C^int^ MCs (**g**) from MCA205-bearing mice 3 days post-treatment with PBS or doxorubicin (n = 5 mice per group). Mean ± s.e.m. is plotted. (**h**) Flow cytometric identification of intra-tumoural tetramer^+^ cell populations and functional phenotypes in mice bearing MCA205-LA-OVA-mCherry tumours treated with doxorubicin. Data are representative of two (**e**-**h**) or three (**a**-**d**) biologically independent experiments. Data were analysed using a Bonferroni-corrected two-way ANOVA (**a**, **d**), or log-rank (Mantel-Cox) test (**a**).

To assess whether enhanced necrotic cell sampling by tumor APCs could increase antitumor immunity in vivo, we administered Fc–DNGR-1 to mice bearing MCA205 fibrosarcoma cells, alongside therapeutic regimens known to induce cancer cell death. We used 2WA Fc–DNGR-1 as a control reagent and treated mice therapeutically after tumor establishment (Fig. 6a). Notably, we observed that WT but not 2WA Fc–DNGR-1 attenuated growth of the tumors when administered with anthracycline doxorubicin (Fig. 6b) or following X-ray radiotherapy (Fig. 6c). Furthermore, dose escalation of Fc–DNGR-1 resulted in progressively enhanced tumor control and overall survival (Extended Data Fig. 8a).

To confirm the importance of Fc-dependent effector functions in vivo, we used a LALA-PG variant of Fc–DNGR-1, with a set of mutations that has been reported to more completely abolish Fc effector functions than N297A^46–48^. We confirmed that LALA-PG Fc–DNGR-1 remained capable of necrotic cell binding (Extended Data Fig. 8b) but exhibited negligible ability to trigger FcγR-dependent cytokine production in RAW264.7 macrophages when plate-coated (Extended Data Fig. 8c). This was in contrast to N297A Fc–DNGR-1, which showed residual FcγR triggering activity at higher concentrations (Extended Data Fig. 8c). In vivo, phosphate-buffered saline (PBS)-, 2WA- or LALA-PG Fc–DNGR-1-treated groups all displayed a lack of tumor control in the doxorubicin-MCA205 model compared to mice treated with WT Fc–DNGR-1 (Fig. 6d). Consistent with a role for cDC1s in tumor control, we observed enhanced tumor growth in *Batf3*^*−/−*^ mice compared to WT C57BL/6 mice when treated with null reagent 2WA (Fig. 6e). However, the ability of WT Fc–DNGR-1 to promote tumor control was retained in *Batf3*^*−/−*^ mice, showing that Fc–DNGR-1 can act via FcγRs expressed by non-cDC1s in this model. The importance of CD8^+^ T cells was confirmed by loss of therapeutic activity in mice treated with depleting anti-CD8α antibodies but not an isotype control (Fig. 6f). Finally, WT Fc–DNGR-1 displayed therapeutic activity in transplantable tumor models other than MCA205, being able to restrain CT26 or MC38 tumor growth as a single agent (Fig. 6g) or in combination with chemotherapy (Extended Data Fig. 8d). The single agent activity suggests that for some tumors, cell death occurring spontaneously in the absence of cytotoxic therapies may be sufficient for Fc–DNGR-1-driven antitumor immunity.

We investigated changes in the TME elicited by Fc–DNGR-1 treatment in combination with doxorubicin. Doxorubicin treatment did not affect the number of tumor cDCs (Extended Data Fig. 8e), but it increased the frequency and absolute numbers of CD88^+^Ly6C^int^ MHC-II^+^ MCs^49^ (Fig. 6h and Extended Data Fig. 8f). These MCs expressed higher levels of FcγRI and FcγRIIB following chemotherapy (Fig. 6i and Extended Data Fig. 8g). As such, synergy between Fc–DNGR-1 and doxorubicin chemotherapy might involve infiltration of antigen-presenting MCs with elevated FcγR expression, as well as increased cell death. Next, we assessed the impact of treatment on the CD8^+^ T cell response, focusing on MCA205-LA-OVA-mCherry tumors treated with doxorubicin. Using SIINFEKL-H2-K^b^ tetramer staining 7 days after treatment initiation to identify OVA antigen-specific cells, we observed a high frequency of PD-1^+^TIM-3^+^ CD8^+^ effector T cells within both tetramer-positive and tetramer-negative compartments, consistent with a high level of responsiveness against OVA, as well as, presumably, endogenous tumor antigens (Extended Data Fig. 8h). Notably, WT Fc–DNGR-1 enhanced the frequency of PD-1^+^TIM-3^*−*^ CD8^+^ T cells, consistent with a progenitor-like phenotype, relative to PD-1^+^ and TIM-3^+^ cells that are associated with T cell exhaustion (Fig. 6j).

### Anti-F-actin antibodies promote necrophagy by human APCs and cell activation

**Extended Data Fig. 9:**
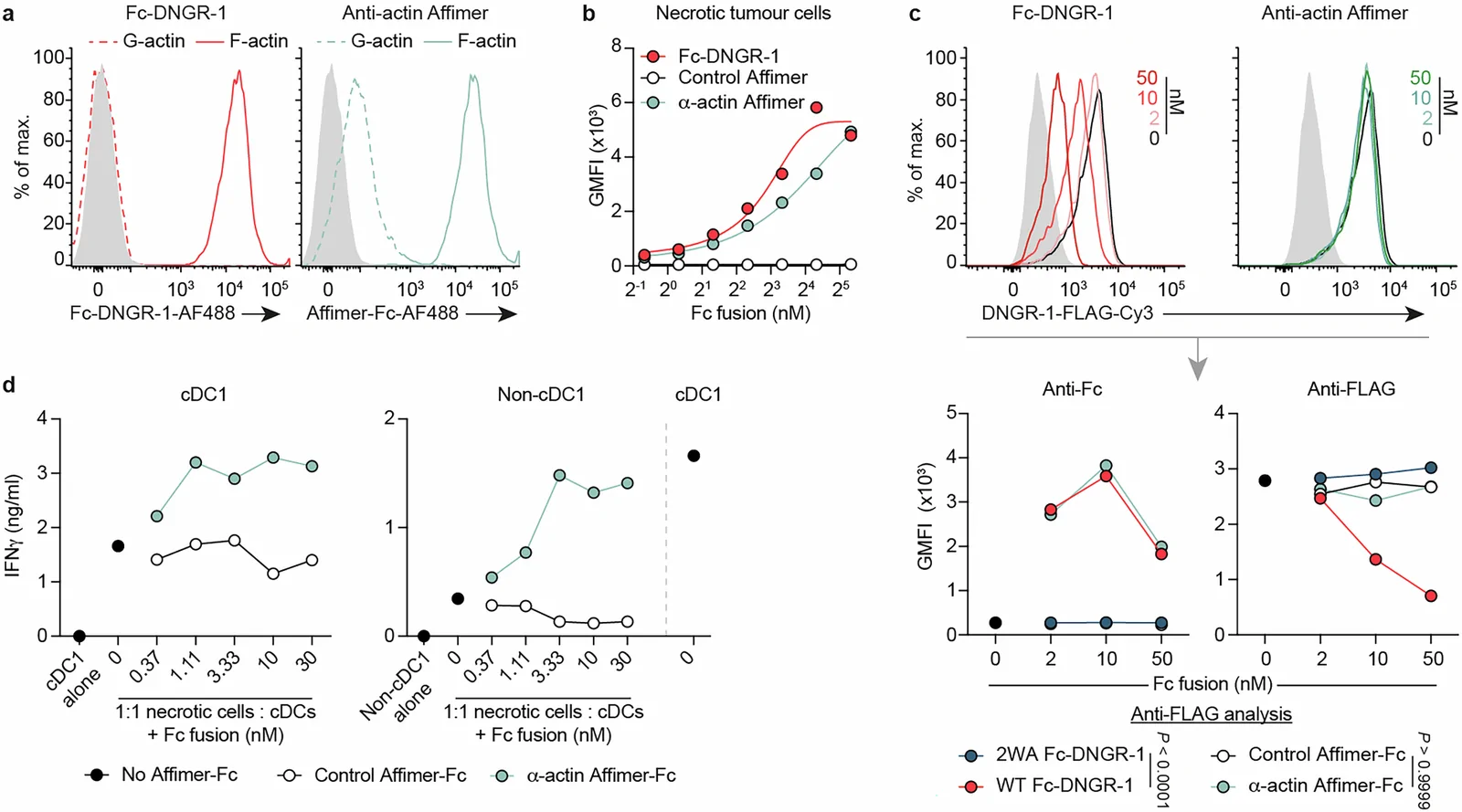
Affimer-Fc as orthogonal F-actin-FcγR bridging molecules. (**a**, **b**) Flow cytometric analysis of Fc-DNGR-1 (red) and anti-actin Affimer-Fc (teal) binding to G-actin (dashed) or F-actin (solid line) coated latex beads (**a**) and UV-irradiated necrotic tumour cells (**b**). Single measurements are plotted. (**c**) Flow cytometric pulse-chase experiment of necrotic tumour cells incubated with Fc-DNGR-1 or Affimer-Fc for 1 h followed by DNGR-1-FLAG for 30 min. Binding of the primary fusion protein (bottom left) and the DNGR-1-FLAG (bottom right) was assessed. Mean from technical duplicates is plotted. (**d**) ELISA for IFN-γ release from OT-I CD8^+^ T cells co-cultured with primary FLT3L-cDCs and OVA-soaked necrotic cells ± Affimer-Fc. Mean from technical duplicates is plotted. Data are representative of two (**a**, **c**, **d**) or three (**b**) biologically independent experiments.

Fc–DNGR-1 represents a proof-of-concept approach for coupling F-actin recognition to FcγRs. In an orthogonal approach, we generated Fc fusions using anti-actin Affimer proteins, which are affinity reagents based on plant-derived phytocystatins^50^. We confirmed that anti-actin Affimer-Fc exhibited preferential binding to F-actin versus G-actin beads (Extended Data Fig. 9a), although this was less absolute than for DNGR-1, and that it stained necrotic tumor cells similarly to Fc–DNGR-1 (Extended Data Fig. 9b). Competition experiments demonstrated that Affimer-Fc bound to a distinct F-actin epitope with little overlap with that bound by the DNGR-1 ECD (Extended Data Fig. 9c). Notably, like Fc–DNGR-1, anti-actin Affimer-Fc, but not control Affimer-Fc, boosted necrotic cell XP by both cDC1s and non-cDC1s (Extended Data Fig. 9d), again normalizing the ability of both cell types to activate CTLs against dead-cell-derived antigens.

**Fig. 7:**
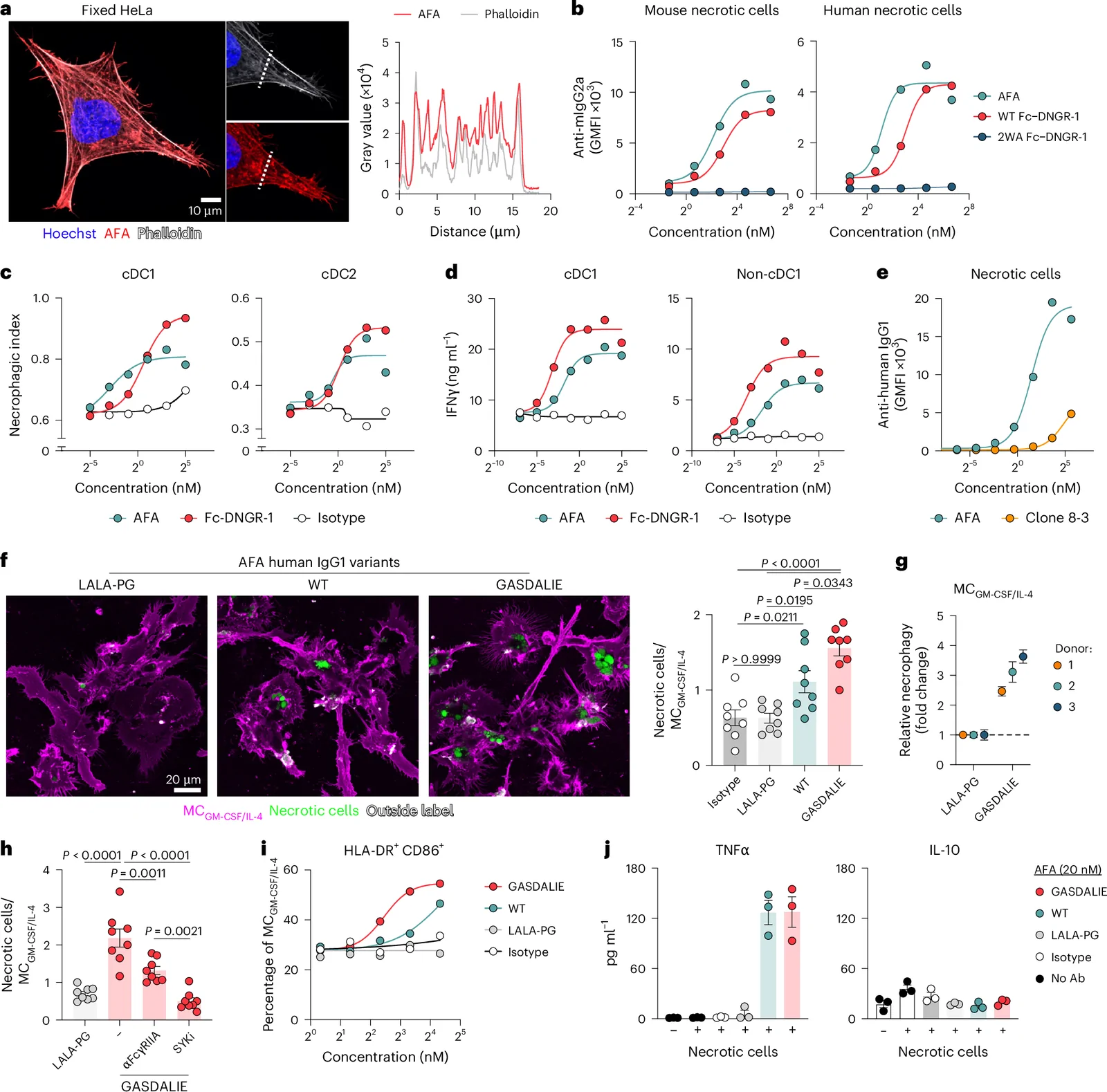
Anti-F-actin antibodies promote necrophagy by human APCs and cell activation. **a**, Confocal microscopy and colocalization analysis of live fixed HeLa cells stained with AFA mouse IgG2a and phalloidin. **b**, Flow cytometric analysis of AFA binding to UV-irradiated mouse and human necrotic tumor cell lines. The mean from technical duplicates is plotted. **c**, Flow cytometric analysis of necrotic cell uptake by FLT3L cDC1s and cDC2s with and without AFA or Fc–DNGR-1 for 4 h. The mean from technical duplicates is plotted. **d**, ELISA for IFNγ release from OT-I CD8^+^ T cells cocultured with FLT3L cDCs, OVA-necrotic cells, and AFA or Fc–DNGR-1. The mean from technical duplicates is plotted. **e**, Necrotic cell binding as in **b** comparing AFA hIgG1 to clone 8-3 hIgG1. Single measurements are plotted. **f**, Confocal microscopy analysis of MC_GM-CSF/IL-4_ cells cultured with necrotic cells and isotype control or AFA hIgG1 variants for 3 h. The mean ± s.e.m. from eight fields of view is plotted. **g**, Summary of LALA-PG- versus GASDALIE-mediated uptake across donors, as in **f**. **h**, Confocal microscopy of MC_GM-CSF/IL-4_ cells as in **f** in the presence of anti-FcγRIIA blocking antibody or SYK inhibitor. The mean ± s.e.m. from eight fields of view is plotted. **i**, Flow cytometric analysis of HLA-DR and CD86 upregulation on MC_GM-CSF/IL-4_ cells treated with necrotic cells (4:1) and increasing concentrations of isotype or AFA hIgG1 variants. The mean from technical duplicates is plotted. **j**, Flow cytometric multiplex cytokine analysis for MC_GM-CSF/IL-4_ cells treated with 20 nM AFAs and necrotic cells as in **i**. The mean ± s.e.m. from technical triplicates is plotted. Data are representative of two (**a**, **c**, **e** and **h**) or three (**b**, **d**, **f**, **i** and **j**) biologically independent experiments. Data in **f** and **h** were analyzed using one-way ANOVA. Ab., antibody.

**Extended Data Fig. 10:**
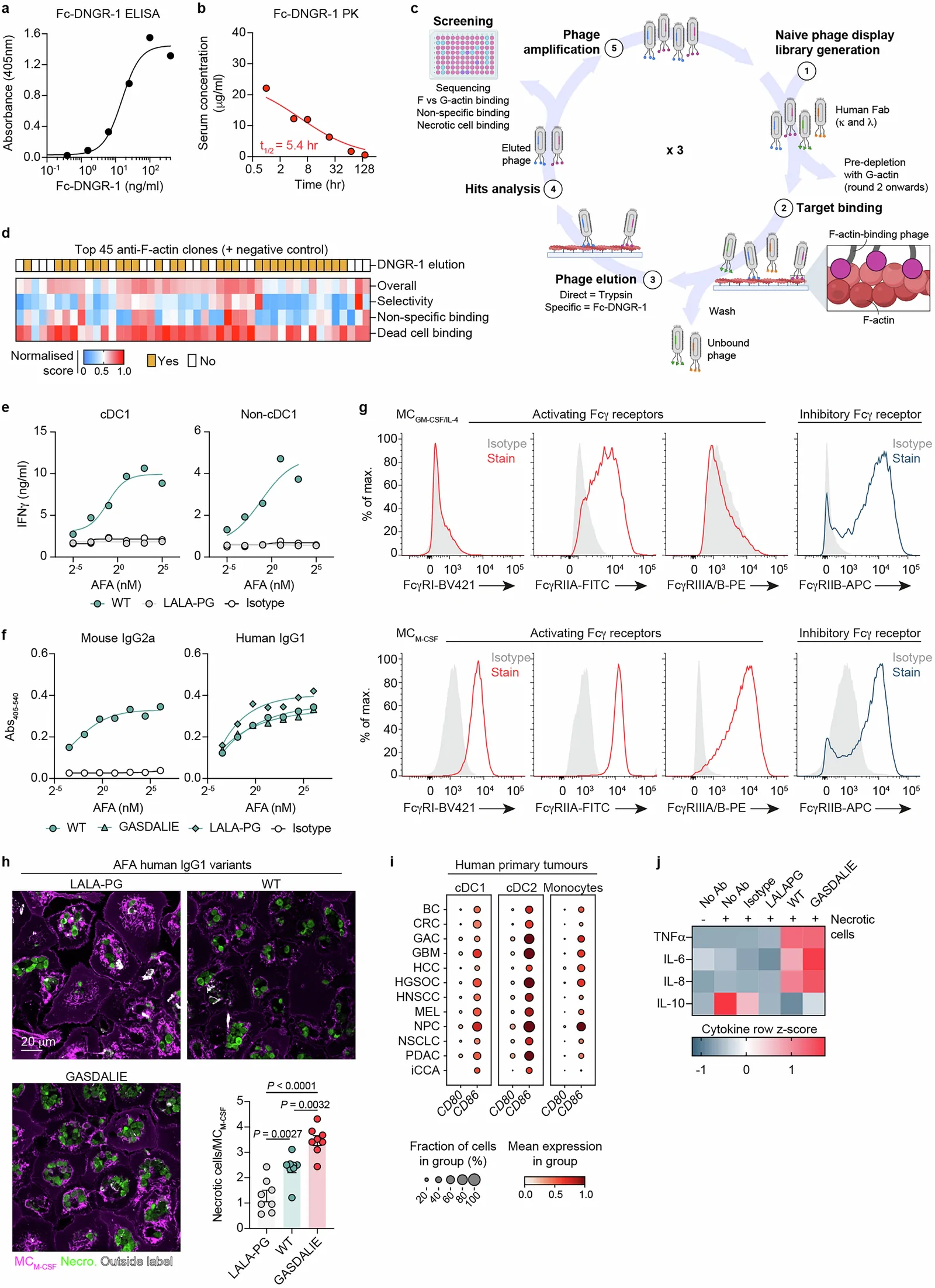
Anti-F-actin IgG enhances necrotic cell sensing by human APCs. (**a**) Standard curve of anti-DNGR-1 ELISA for PK analysis. Single measurements are plotted. (**b**) ELISA of serum Fc-DNGR-1 levels following systemic administration (n = 2 mice per timepoint). Mean is plotted. (**c**) Schematic of phage display pipeline for selection of F-actin-specific antibody clones. (**d**) Heatmap of selection criteria for top-performing anti-F-actin clones. Orange = clone eluted with Fc-DNGR-1; white = clone eluted with trypsin. (**e**) ELISA for IFN-γ release from OT-I CD8^+^ T cells co-cultured with FLT3L-cDCs, OVA-necrotic cells and WT or LALA-PG AFA mIgG2a. Mean from technical duplicates is plotted. (**f**) ELISA analysis of AFA mIgG2a and hIgG1 variants for binding to F-actin-coated ELISA plates. Single measurements are plotted. (**g**) Flow cytometric analysis of FcγR expression on human MC_GM-CSF/IL-4_ and MC_M-CSF_. (**h**) Confocal microscopy analysis of MC_M-CSF_ cells cultured with necrotic cells and AFA hIgG1 variants for 3 h. Mean ± s.e.m. from 8 fields of view is plotted. (**i**) Co-stimulatory molecule expression on human primary tumour cDC1s, cDC2s, and classical monocytes. Data derived from scRNAseq dataset in^26^. Gene expression values capped at 1. Sample numbers as per Extended Data Fig. 1a and b. (**j**) Flow cytometric multiplex cytokine analysis of MC_GM-CSF/IL-4_ treated with necrotic cells (4:1) and AFA hIgG1 variants. Mean from technical triplicates is plotted. Data are representative of two (**a**, **b**, **e**, **f**) or three (**g**-**j**) biologically independent experiments. Data were analysed using a one-way ANOVA (**h**). Schematic in **c** created in BioRender; Castro Dopico, T. https://biorender.com/jyc1ixc (2026).

Fc fusion proteins often exhibit a shorter half-life than antibodies in vivo^51^. We developed a serum-compatible enzyme-linked immunosorbent assay (ELISA) to track Fc–DNGR-1 in circulation (Extended Data Fig. 10a) and found a half-life of ~5 h following systemic administration (Extended Data Fig. 10b). To provide greater clinical applicability, we generated new anti-F-actin monoclonal antibodies via phage display screening of a naive human Fab library (Extended Data Fig. 10c). After successive filtering stages, we identified 45 clones with optimal characteristics, including F-actin specificity, human and mouse necrotic cell staining, and minimal off-target binding (Extended Data Fig. 10d). One selected anti-F-actin clone (termed AFA) was synthesized as a chimeric full-length mouse IgG2a antibody. AFA colocalized with phalloidin-stained F-actin filaments in fixed cells (Fig. 7a), bound to human and mouse necrotic cells (Fig. 7b), and promoted uptake of necrotic debris and XP of associated antigens by both murine cDC1s and non-cDC1s, much like Fc–DNGR-1 (Fig. 7c,d). Furthermore, XP of dead-cell-associated antigens was abolished using a LALA-PG-mutant AFA (Extended Data Fig. 10e).

We formulated AFA as a human IgG1 (hIgG1) for investigation with human myeloid cells, as that isotype binds optimally to human FcγRs. We also generated LALA-PG and GASDALIE mutant versions that abolished or enhanced FcγR binding, respectively, but retained comparable binding to F-actin (Extended Data Fig. 10f). Notably, an anti-F-actin antibody was recently identified from an expanded plasma cell clone in a pancreatic adenocarcinoma patient (clone 8-3)^52^. However, when expressed as hIgG1 and compared head-to-head with AFA, 8-3 bound only weakly to necrotic cells and was not investigated further (Fig. 7e).

Human cDC2s and MCs, like those in mice, lack DNGR-1 expression^53^ and are poorly necrophagic^54^. We assessed AFA hIgG1 function with two different human peripheral blood CD14^+^ monocyte-derived populations. First, we generated DC-like MCs (MC_GM-CSF/IL-4_), which expressed FcγRIIA and FcγRIIB, akin to cDC2s (Extended Data Fig. 10g). These cells displayed low necrophagic ability under basal conditions or in the presence of LALA-PG AFA (Fig. 7f). However, across human donors, addition of WT or GASDALIE AFA promoted necrophagy (Fig. 7f,g). Consistent with FcγR expression patterns, AFA-induced uptake of necrotic debris could be inhibited using a blocking antibody to FcγRIIA or a SYK inhibitor (Fig. 7h). Similar observations were made with human monocyte-derived macrophages (MC_M-CSF_). These cells expressed high levels of all FcγRs (Extended Data Fig. 10g) and displayed elevated basal necrophagic activity compared to MC_GM-CSF/IL-4_. Nevertheless, WT and GASDALIE AFA again substantially enhanced necrophagy, which was particularly noticeable with respect to the number of necrotic corpses internalized per MC_M-CSF_ (Extended Data Fig. 10h).

Finally, we assessed the functional consequences of enhanced necrophagy by human MCs. Human cDC1s, cDC2s and monocytes exhibited similar levels of costimulatory molecules across cancer types, suggesting that AFA-induced necrophagy by diverse APCs may support T cell responses in humans (Extended Data Fig. 10i). We assessed whether AFA could further enhance cellular activation in MC_GM-CSF/IL-4_ in the presence of necrotic cells. Little upregulation of HLA-DR and CD86 (Fig. 7i) or production of inflammatory cytokines (Fig. 7j and Extended Data Fig. 10j) was observed with an isotype control or the LALA-PG AFA. By contrast, addition of WT or GASDALIE AFA enhanced both costimulatory molecule and cytokine expression, most notably inducing TNF-α but not IL-10 production. Collectively, these data demonstrate that AFAs can act across species to promote dead cell uptake and enhance functions of non-cDC1 APCs.

## Discussion

cDC1s can couple detection of necrotic tumor cells to priming and restimulation of tumor antigen-specific CD8^+^ T cells to sustain immunity to cancer^15^^,^^55^. By contrast, cDC2s, MCs and macrophages have less prominent roles in XP of cancer antigens, even though, as shown here, they are far more abundant than cDC1s within tumors and are positioned closer to areas of tumor necrosis. We show that linking F-actin recognition to FcγRs is an effective means of allowing non-cDC1 APCs to become as effective as cDC1s at taking up necrotic cell debris and cross-presenting dead-cell-associated antigens. Furthermore, we identify phagosomal damage as a cellular mechanism driving antigen translocation downstream of FcγR triggering for access to the cytosolic MHC-I pathway. Although we found a dominant role for FcγRI in this process in vitro with mouse APCs, future work will be necessary to determine the precise role of individual FcγRs in vivo, as well as how FcγRs couple to phagosomal rupture across diverse myeloid populations such as cDC1s, cDC2s and MCs. How AFAs couple necrotic cell sensing to XP in human APCs is also an area of ongoing investigation. This will require establishing an assay with sufficient sensitivity to detect XP of dead cell-associated antigen, which has eluded us thus far. Nevertheless, our data in mice support the concept that XP is not a constitutive property of a single APC (that is, cDC1s), but one that can be induced by specific receptor signals and could therefore be exploited therapeutically for antitumor immunity. Although we focused on XP of necrotic-cell-associated antigens in this study, it will be important to determine how other mechanisms collectively contribute to tumor control. Indeed, unlike DNGR-1, FcγR signaling triggers myeloid cell activation and effective presentation on MHC-II to CD4^+^ T cells^22,56^, which may further support the activity of cDC2s in an effective antitumor immune response. Furthermore, how the activation state of distinct tumor APC populations affects these FcγR functions warrants further investigation.

The approach of linking F-actin recognition to FcγRs is agnostic to de novo or overexpressed tumor-specific (neo)antigens, instead targeting a cellular process, that is, cell death. The latter is common in tumors and may occur spontaneously or in response to treatment, including chemotherapy, radiation and antibody–drug conjugates^12^. We show that Fc–DNGR-1 promotes therapeutic tumor control and synergizes with chemotherapy and radiotherapy in preclinical models. Critically, broadening XP of necrotic cell antigens may both enhance and, through epitope spreading, diversify an ongoing anticancer CTL response, as well as induce CTLs in immune-cold tumors devoid of cDC1s. Consistent with the latter, we demonstrate that in vivo efficacy of Fc–DNGR-1 is preserved in mice deficient in cDC1s. We also show that in mouse tumors, cDC1s are located more distally to necrotic areas than cDC2s and MCs, highlighting the therapeutic value of targeting necrotic cell debris to FcγR^+^ non-cDC1s. The development of new anti-F-actin monoclonal antibodies may provide greater versatility and clinical applicability going forward. Future studies will be essential to assess how these agents perform in immune-cold and autochthonous/orthotopic tumor models, and how observations in mice translate to human tumors and AFA efficacy with clinically relevant regimens, including immune checkpoint blockade.

Our approach is based on a natural system for coupling damage-associated molecular pattern (DAMP) detection to XP of dead-cell-associated antigens, revealing how immune mechanisms regulating antigen presentation can be exploited therapeutically. Strikingly, clonally expanded anti-F-actin IgG-producing plasma cells have recently been reported in human pancreatic ductal adenocarcinoma, suggesting that similar mechanisms may naturally contribute to tumor control in cancer patients^43^. In addition to DNGR-1, other DAMP receptors and/or their intracellular targets could be harnessed to enhance dead cell immunogenicity. In fact, it is possible that this happens naturally in some autoimmune diseases, in which DAMP-specific autoantibodies are common^57^. Therapeutic drugs akin to Fc–DNGR-1 could be designed to enhance or suppress immunity to dead cells for use beyond cancer, including viral infection, vaccination or autoimmunity.

## Methods

### Human

Blood was obtained from anonymous healthy adult volunteers with informed consent according to approved protocols of the ethics board of the Francis Crick Institute and the Human Tissue Act (study reference CREC_2024_006). Sex, gender, race and ethnicity were not considered in the study design. All samples were destroyed following analysis.

### Mice

WT, *Batf3*^*−/−*^^3^, *Clec9a*^*Cre*^*Rosa*^*LSLtdTomato*^^44^, *Xcr1*^*Venus*^ (gift from T. Kaisho, Wakayama Medical University, Japan)^45^ and OT-I/*Rag1*^*−*/*−*^ mice on a C57BL/6 background and WT BALB/c mice were bred and maintained at The Francis Crick Institute under specific-pathogen-free conditions, a 12 h light/dark cycle (7 a.m. to 7 p.m.), and controlled room temperature (RT; 20–24 °C) and humidity (55% ± 10%). Sex-matched female and male mice were used at 6–10 weeks of age for in vivo tumor experiments. No statistical methods were used to predetermine sample sizes, but these sizes were similar to those reported in previous publications^8,43^. All animal experiments were performed in accordance with national and institutional guidelines for animal care and were approved by the Francis Crick Institute Biological Resources Facility Strategic Oversight Committee (incorporating the Animal Welfare and Ethical Review Body) and by the Home Office, United Kingdom (PP8593771).

### Primary cells and lines

RPMI 1640 supplemented with 2 mM glutamine, 100 units ml^−1^ penicillin, 100 μg ml^−1^ streptomycin, nonessential amino acids, 10 mM HEPES, 50 μM 2-mercaptoethanol (all from Gibco) and 10% heat-inactivated fetal calf serum (FCS) (R10^+^ medium) was used for all cell culture unless otherwise stated. The MutuDC1940 cDC1 line^58^ was a gift from H. Acha-Orbea (University of Lausanne, Switzerland) and cultured in IMDM containing 10% heat-inactivated FCS, 100 units ml^−1^ penicillin, 100 μg ml^−1^ streptomycin and 50 μM 2-mercaptoethanol. BRAF^V600E^ 5555 melanoma was a gift from G. Kassiotis (The Francis Crick Institute, UK). MCA205 fibrosarcoma, CT26 colorectal cancer, HeLa, and RAW264.7 parental and sublines were obtained from The Francis Crick Institute Cell Services Science Technology Platform. MCA205-LA-OVA was generated previously^43^.

For murine FLT3L cDCs, bone marrow was extracted from hind legs of mice and subjected to red blood cell lysis (Thermo Fisher Scientific). Cells were cultured for 9 days in R10^+^ medium containing 150 ng ml^−1^ recombinant mouse FLT3L (R&D systems). On day 8, FTL3L cDC cultures were additionally primed with 200 ng ml^−1^ IFNα (R&D Systems). cDC1s and non-cDC1s were separated using biotinylated anti-mouse XCR1 IgG (BioLegend, clone ZET) and Anti-Biotin MicroBeads and LS Columns (both Miltenyi) according to the manufacturer’s instructions. Alternatively, cDC2s were enriched on day 9 using PE-conjugated anti-mouse CLEC10A IgG (clone LOM-14, BioLegend) and Anti-PE MicroBeads (Miltenyi), followed by overnight priming with 200 ng ml^−1^ IFNα. In some experiments, FLT3L cDC1s and cDC2s were surface-stained and flow-sorted using a FACSAria (BD Biosciences). GM-CSF- and M-CSF-derived cells were generated by bone marrow cell culture with 20 ng ml^−1^ GM-CSF for 7 days^59^ or M-CSF-containing L929 conditioned medium, respectively.

For preactivated effector OT-I CD8^+^ T cells, spleen and lymph node single-cell suspensions from OT-I/*Rag1*^*−*/*−*^ mice were subjected to red blood cell lysis before culture in R10^+^ medium supplemented with 100 U ml^−1^ IL-2 (Peprotech) and 0.1 nM SIINFEKL (generated at The Francis Crick Institute, UK) for 3 days. On days 3 and 4, cells were split 1:2, and the culture medium was completely replaced with R10^+^ medium containing 100 U ml^−1^ IL-2. OT-I cultures were used on day 5.

For human MCs, peripheral blood mononuclear cells were isolated using Histopaque-1077 (Thermo Fisher Scientific) per the manufacturer’s instructions, and CD14^+^ monocytes were enriched using biotinylated anti-human CD14 IgG (clone HCD14, BioLegend) as outlined for FLT3L-DCs. For MC_GM-CSF/IL-4_ and MC_M-CSF_, monocytes were cultured with either 100 ng ml^−1^ recombinant human GM-CSF and 40 ng ml^−1^ IL-4 or 50 ng ml^−1^ M-CSF (all Peprotech) in R10^+^ medium for 6 days, respectively, with half the medium refreshed on day 3.

### Hoxb8 CDP generation and culture

CDPs were immortalized using the ER-Hoxb8 system^31^. Bone marrow CDPs (Lin^−^CD117^−^CD115^+^FLT3^+^DNGR-1^+^) were isolated by fluorescence-activated cell sorting (FACS) and cocultured with congenic bone marrow cells in R10^+^ medium with 20 ng ml^−1^ IL-3, IL-6 and SCF (Peprotech). Cells were transduced the following day with retrovirus containing MSCV-Neo-HA-ER-Hoxb8 (obtained from D. Sykes, Harvard, Cambridge, USA) and selectively expanded with 1 mg ml^−1^ G418 in R10^+^ medium supplemented with 75 ng ml^−1^ FLT3L (from CHO-FLT3L-producing cells) and 0.5 µM E2 (β-estradiol, Sigma-Aldrich). Immortalized CDPs were recovered by FACS. Passaged Hoxb8 CDPs were washed twice with Dulbecco’s PBS (DPBS) and cultured in R10^+^ medium with 75 ng ml^−1^ recombinant mouse FLT3L (R&D systems) alone (for cDC2s) or with OP9-DL1 cells (for cDC1s and cDC2s)^60^ over 5–7 days.

### Myeloid APC transcriptomic analyses

For human tumor APC analysis, we used a recently generated myeloid APC atlas based exclusively on public datasets (listed in Supplementary Table 3) as outlined previously^26^. Code and data availability are outlined below. cDC1s, cDC2s and classical monocytes were extracted from the scvi batch-corrected object. Standard Scanpy (v.1.10.2) pipelines were used to generate a neighborhood graph (scanpy.pp.neighbors() function, n_neighbors=10, n_pcs=30) and UMAP visualization (scanpy.tl.umap() function, min_dist=0.3) in Python (v.3.12.3). To generate dot plots of FcγRs and costimulatory molecules across tissues, we used the scanpy.pl.dotplot() function, with dot_min=0.0, dot_max=1.0, vmin=0, vmax=1. Cell counts and associated metadata were extracted from the atlas and used to calculate frequencies for the cell types of interest. *Fcgr* expression was assessed on distinct steady state and inflammatory APCs using the Immgen database.

### Murine tissue analysis and flow cytometry

Excised murine tumors and spleens were diced and digested with collagenase IV (200 U ml^−1^, Worthington) and DNase I (100 μg ml^−1^, Sigma-Aldrich) for 30 min at 37 °C. Tissue was filtered through 70-μm strainers (Falcon), washed with FACS buffer (DPBS with 1% FCS and 2 mM EDTA) and pelleted by centrifugation at 400*g* for 4 min. For tumors, single-cell suspensions were resuspended in 40% Percoll and centrifuged at 600*g* for 20 min with minimal acceleration and deceleration.

Single-cell suspensions were stained with LIVE/DEAD Fixable Blue Dead Cell Dye (Thermo Fisher Scientific) per the manufacturer’s instructions and stained for 30 min on ice with a combination of fluorescently conjugated anti-mouse antibodies (listed in Supplementary Table 4). For human FcγR analysis, MCs were harvested on ice or with 1× TrypLE (Thermo Fisher Scientific), washed twice in DPBS, and surface-stained with the antibodies listed in Supplementary Table 4. Cells were acquired live or fixed (Nordic-MUbio). Within each experiment, identical acquisition parameters were used across tissues, allowing comparison of GMFI values. For live cells, DAPI (2 ng ml^−1^) was used for dead cell discrimination in certain instances. Cell quantification was performed using 123count eBeads (Fisher Scientific). Samples were acquired on an LSRFortessa or FACSymphony (BD Biosciences), and data were analyzed using FlowJo v.10.

### Fc fusion proteins

Fc–DNGR-1 fusion proteins were generated at The Francis Crick Institute or contracted commercially (ImmunoPrecise, The Netherlands). Briefly, WT and 2WA mouse DNGR-1 ECD DNA sequences were amplified from existing pFB neo plasmids using Infusion-designed primers (Sigma-Aldrich). An amplified pFUSEN-mG2aFc plasmid (InvivoGen) was linearized using NheI and EcoRV restriction enzymes, gel purified, and subjected to Infusion reaction (Takara) with amplified 2WA or WT mouse DNGR-1 ECD DNA sequences according to the manufacturer’s instructions. Plasmids were transformed into Stellar competent cells and selected overnight on zeocin agar plates. Single colonies were cloned, sequenced and used for downstream expression. Mouse IgG2a N297A Fc mutagenesis was performed with sequence-verified Fc–DNGR-1 and a QuikChange Lightning Site-directed Mutagenesis Kit (Agilent Technologies) according to the manufacturer’s instructions, using primers designed to span the mutation site (Sigma). Plasmids were transformed into XL10 Gold bacteria (Agilent Technologies) and grown overnight on LB agar plates. Single colonies were grown in zeocin-containing LB broth, and plasmid DNA was extracted using a QIAprep Spin Miniprep Kit (QIAGEN) and sequenced.

Amplified sequence-verified plasmids were transiently transfected into Expi293F cells, and supernatants were harvested over several days. Fc–DNGR-1 fusion proteins were purified using protein A beads (Generon; M1300-5) following the protocol for the Pierce Gentle Ag/Ab Binding and Elution Buffer Kit (Thermo Fisher Scientific, 21030). Proteins were then dialyzed in endotoxin-free 25 mM Tris pH 7.2, 150 mM NaCl (BupH Tris Buffered Saline Packs, Thermo Fisher Scientific). Samples were tested for endotoxin (Pierce Chromogenic Endotoxin Quant Kit-60 reactions, Thermo Fisher Scientific) and confirmed to be <0.05 EU ml^−1^. Nonreducing SDS–PAGE was performed on purified proteins by heating to 95 °C for 5 min in Laemmli buffer and running on 7.5% mini-PROTEAN TGX precast gels (Bio-Rad) with Precision Plus Dual Color Standards. Gels were developed with Coomassie Brilliant Blue R-250 Staining Solution (Bio-Rad) and analyzed on an ImageQuant 800 (Amersham) imaging system. Large-scale production of fusion proteins was contracted commercially (ImmunoPrecise, The Netherlands; and FairJourney Biologics, Portugal).

### Anti-F-actin human phage display

Anti-F-actin IgG monoclonal antibody selection was performed using phage display with a naive human Fab library and was contracted commercially (FairJourney Biologics, Portugal). After three rounds of positive selection for F-actin binding and negative selection for G-actin binding, a total of 736 clones were identified. For each round of screening, F-actin-bound phages were eluted either using standard elution buffer containing trypsin (1 mg ml^−1^) or via competition with Fc–DNGR-1 (at 1000 nM). ELISAs were performed to determine binding to F-actin and G-actin. Following Fab sequencing and analysis, including VH and VL gene diversity and sequence liabilities, 78 unique clones were taken forward for further evaluation. Necrotic cell binding analysis led to identification of 68 clones with ≥3-fold dead cell selectivity; these were reformatted to murine IgG2a and filtered for: ≥5-fold F-actin over G-actin binding, ≥5-fold F-actin over neutravidin binding, ≥10% necrotic cell binding, and ≥3-fold necrotic cell GMFI over background GMFI. Forty-five clones met these thresholds. Production of endotoxin-free anti-F-actin mouse IgG2a and hIgG1 monoclonal antibodies was contracted commercially (ImmunoPrecise, The Netherlands; and Biointron, China).

### OVA-IC generation

Soluble albumin from chicken egg white (OVA, 1 mg ml^−1^ in DPBS, Thermo Fisher Scientific) was incubated 1:1, 10:1, or 100:1 (v/v) with rabbit anti-chicken egg albumin whole antiserum (Sigma-Aldrich) at 37 °C for 30 min. In some experiments, OVA was labeled with an Alexa Fluor 647 (AF647) labeling kit (Thermo Fisher Scientific), per the manufacturer’s instructions.

### FcγR cross-linking

Ninety-six-well high-affinity Nunc MaxiSorp plates (Thermo Fisher Scientific) were coated with Fc–DNGR-1 or mouse IgG1/IgG2a (BioLegend) in DPBS overnight at RT. Plates were blocked with DPBS containing 10% FCS for 1 h. RAW264.7 cells were plated at 1 × 10^5^ cells per well in R10^+^ medium for 24 h. Mouse TNF and CXCL2 were measured using R&D Systems DuoSet kits per the manufacturer’s instructions.

### Preparation of FM beads

As previously described^16,61^, lyophilized nonbiotinylated G-actin (Cytoskeleton) was reconstituted in sterile water (10 mg ml^−1^), diluted in G-buffer (1 mg ml^−1^) and mixed at a 1:1 molar ratio with freshly reconstituted biotinylated G-actin (Cytoskeleton) in F-actin buffer. After 1 h at RT, myosin-II (Cytoskeleton) was added to F-actin at a 1:1 molar ratio and incubated for a further 1 h at RT. For coating of microbeads, 2 μg ml^−1^ biotin–OVA (generated with a DSB-X biotinylation kit and protocol; Thermo Fisher Scientific) was added to streptavidin-coated 2-μm microbeads (Polysciences) for 1 h at 4 °C. Beads were washed with 1% bovine serum albumin (BSA) in DPBS for 3 min at 10,000*g* then incubated with biotinylated FM for 1 h at 4 °C.

### IgG-OVA bead preparation

Fifty milligrams of 3-μm carboxylated silica beads (Kisker) were washed three times with DPBS, resuspended, and rotated in 40 mg ml^−1^ cyanamide in DPBS for 15 min at RT. The beads were washed three times with 1 ml of 0.1 M sodium borate buffer pH 8.0, resuspended, and rotated in 1 ml of 4 mg ml^−1^ OVA in 0.1 M sodium borate buffer pH 8.0 for 2 h at RT. The beads were then washed another three times with 0.1 M sodium borate buffer pH 8.0 and resuspended and rotated in 1 μg ml^−1^ AF555 succinimidyl ester for 15 min in 0.1 M sodium borate buffer pH 8.0 in the dark. Then, they were serially washed with 1 ml of DPBS, three times with 1 ml of 0.2 M glycine in DPBS, and three times in DPBS before storage at 4 °C. To make IgG-OVA beads, 1:500 polyclonal rabbit anti-OVA IgG (Sigma-Aldrich SAB4301164) was added to 1 ml of OVA beads in DPBS, followed by rotation at RT for 30 min. IgG-OVA beads were then washed three times with 1 ml of PBS and used on the same day. In some instances, streptavidin-coated silica beads (Kisker) were coated with biotin–OVA (as above) before incubation with polyclonal rabbit anti-OVA IgG.

### Necrotic cell preparation

Tumor cell lines (BRAF^V600E^, MCA205 or HeLa) were irradiated with 240 mJ cm^−2^ ultraviolet C (UVC) in DPBS and cultured overnight in serum-free RPMI 1640 medium.

### Binding of Fc fusion proteins to dead cells and FM beads

Necrotic tumor cells or FM beads were incubated with Fc fusion proteins at RT for 1 h in DPBS. Samples were washed twice and incubated with anti-mouse IgG or DNGR-1 antibodies (Supplementary Table 4) at 4 °C for 30 min. Samples were washed and resuspended in DPBS before acquisition and analysis as above. Some samples were washed and incubated with Annexin binding buffer and Annexin V AF647 conjugate (Invitrogen) for 10 min at RT, followed by incubation with DAPI before acquisition.

### Flow cytometric phagocytosis assay

Tumor cells were labeled with Cell Tracker-Deep Red (CT-DR) dye (Thermo Fisher Scientific; 1:1,000 dilution) in DPBS containing Ca/Mg for 30 min at 37 °C before UV irradiation, incubation with phagocytes at the indicated ratios and time points, and surface-staining for flow cytometry with a combination of antibodies (listed in Supplementary Table 4). DAPI or LIVE/DEAD Fixable Blue Dead Cell Dye (Thermo Fisher Scientific) was used to exclude noninternalized necrotic cell material. Samples were acquired and analyzed as above. Necrophagic index was calculated as: (%CT-DR^+^ × CT-DR GMFI of CT-DR^+^)/10,000.

### FcγR-mediated myeloid cell activation

In certain experiments, cytokine production in necrotic cell–phagocyte cocultures was assessed after 24 h using ELISA DuoSet kits (R&D Systems) or LegendPlex (BioLegend) according to the manufacturer’s instructions. Human MCs were further washed three times in DPBS and surface-stained for flow cytometry for 30 min on ice with the anti-human antibodies listed in Supplementary Table 4. LIVE/DEAD Fixable Blue Dead Cell Dye (Thermo Fisher Scientific) was used to exclude dead cells, and samples were analyzed by flow cytometry as above.

### XP assay

APCs (5 × 10^4^ per well) were plated in U-bottomed 96-well plates. Necrotic cells were soaked for 1 h at 37 °C in 10 mg ml^−1^ OVA (Sigma-Aldrich) in RPMI 1640 medium before being washed three times in DPBS. OVA-necrotic cells, FM-OVA beads or SIINFEKL peptides were incubated with APCs for 4 h in the presence of Fc–DNGR-1, Affimer-Fc or AFA variants (10 nM unless specified). Then, 2:1 preactivated OT-Is were added to APC cultures for 24 h, and T cell-derived IFNγ release was measured by in-house ELISA. Briefly, 96-well high-affinity Nunc MaxiSorp plates (Thermo Fisher Scientific) were coated overnight with rat anti-mouse IFNγ IgG (clone R4-6A2, BD Biosciences, 8 μg ml^−1^ in 0.1 M sodium bicarbonate buffer) before being washed in 0.05% Tween-20 in DPBS. Plates were blocked for 1 h with 3% FCS (blocking buffer), washed, and incubated with T cell culture supernatants and recombinant IFNγ (Peprotech) standard curve samples for 2 h. After washing, plates were incubated with biotin rat anti-mouse IFNγ IgG (clone XMG1.2, BD Biosciences, 1 μg ml^−1^ in blocking buffer) for 2 h, then with ExtrAvidin-Alkaline Phosphatase (Sigma-Aldrich, 1:5,000 in blocking buffer) for 30 min, before being developed with SIGMAFAST *p*-nitrophenyl substrate solution (Sigma-Aldrich) per the manufacturer’s instructions. Absorbances at 405 nm (IFNγ signal) and 540 nm (background) were measured after 20–30 min on a Spark plate reader (Tecan). For inhibitor studies, APC-T cell cocultures were also treated with lactacystin (10 μM, Sigma-Aldrich) or leupeptin (250 μM, Sigma-Aldrich) for the duration of the experiment.

CD69 and CD25 upregulation on naive OT-I T cells was assessed by flow cytometry. Naive CD8^+^ T cells from spleen and lymph nodes of OT-I/*Rag1*^*−/−*^ mice were negatively selected using an EasySep mouse CD8^+^ T cell kit (STEMCELL Technologies) according to the manufacturer’s instructions. After 16 h of incubation with APCs, T cells were then stained with anti-mouse antibodies (listed in Supplementary Table 4) and acquired and analyzed as detailed above.

### DNGR-1 cross-blocking assay

Necrotic cells were incubated with Fc fusion proteins at RT for 1 h before addition of 0.5 μg ml^−1^ DNGR-1–FLAG for 30 min and staining with anti-FLAG and IgG antibodies (Supplementary Table 4) at 4 °C for 30 min for flow cytometric analysis as above.

### CRISPR–Cas9-mediated gene editing

As outlined in ref. ^62^, 25 μg recombinant Cas9 nuclease V3 and 40 μM target single-guide RNA (sgRNA) (IDT) were complexed in the presence of 1.6 μM IDT Alt-R Cas9 Electroporation Enhancer per reaction for 25 min at RT. Two sgRNA guides were used per gene. Then, 2 × 10^7^ bone marrow cells were mixed with each ribonucleoprotein complex in primary nucleofection solution (P3) in a 1.5-ml Eppendorf and transferred to a Nucleocuvette. The cell–ribonucleoprotein mixture was electroporated with the CM-137 program in a 4D Nucleofector (Lonza) before transfer to prewarmed medium (R10^+^ without antibiotics). Cells were cultured for 9 days in the presence of 150 ng ml^−1^ FLT3L, and gene deletion was assessed by flow cytometry. The sgRNA sequences are detailed in Supplementary Table 5.

### APC microscopy

For murine cDC imaging, glass coverslips (18 mm) were coated with 0.1% poly-L-lysine (Sigma-Aldrich) for 30 min at RT, washed three times with DPBS, and incubated with 2.5% glutaraldehyde (Sigma-Aldrich) for 15 min at RT. After being washed a further three times with DPBS, coverslips were incubated with 1 μg ml^−1^ anti-MHC-II (Supplementary Table 4) in DPBS for 30 min at RT. Coverslips were washed in DPBS and incubated overnight in 0.2 M glycine in DPBS at 4 °C, before being washed another three times with fresh DPBS; then, 2.5–5.0 × 10^5^ APCs were allowed to attach for 1 h at 37 °C before challenge with CT-DR-labeled necrotic cells with or without Fc–DNGR-1 for 3–4 h. Cells were fixed in 4% paraformaldehyde (PFA), washed three times with DPBS, and stained with rhodamine-conjugated wheat germ agglutinin (1:10,000) in blocking buffer for 30 min at RT. Washed coverslips were mounted onto glass slides using Prolong Diamond Antifade Mountant (Fisher Scientific). Samples were imaged on a Zeiss LSM880 inverted confocal microscope. Image processing and analysis was performed using Fiji/ImageJ or Imaris v.9.1.2.

For human MC imaging, coverslips were prepared as above using anti-human HLA-DR IgG (Supplementary Table 4). CT-DR or CT-green CMFDA-labeled necrotic cells with or without AFAs or isotype control (anti-HEL) were added to plated MCs for 2–3 h at 37 °C. Cells were incubated with LIVE/DEAD Fixable Violet Dye (Thermo Fisher Scientific) in DPBS for 20 min, washed three times in DPBS, fixed in 4% PFA, washed another three times in DPBS and stained with rhodamine-conjugated wheat germ agglutinin (1:10,000) in blocking buffer for 30 min at RT. Washed coverslips were mounted onto glass slides using Prolong Diamond Antifade Mountant (Fisher Scientific) and analyzed as above.

### Tumor microscopy

For tumoroids, 5 × 10^3^ BRAF^V600E^ 5555 melanoma cells were seeded into Corning Matrigel Basement Membrane Matrix (VWR) in R10^+^ medium and cultured for 5 days with constant agitation. Tumoroids were dissociated from Matrigel using Corning Cell Recovery Solution (Fisher Scientific) and incubated with 10 μg ml^−1^ Fc–DNGR-1, AF555 phalloidin (1:400, Fisher Scientific) or 2 μg ml^−1^ DAPI for 24 h in R10^+^ medium. Tumoroids were washed in DPBS for 1 h, fixed in 4% PFA for 1 h, then incubated in 30% sucrose overnight. For tumors, 2.5 × 10^6^ MCA205-LA-OVA-mCherry tumor cells were injected into the shaved flanks of C57BL/6, *Clec9a*^*Cre*^*Rosa*^*LSLtdTomato*^ or *Xcr1*^*Venus*^ mice. After 7 days, Fc–DNGR-1 was injected i.t. (50 μg) or subcutaneously (s.c.; 200 μg) in DPBS, and tumors were harvested after 6–24 h. Tumors were fixed and processed as per tumoroid samples.

Tumoroids and tumors were embedded in O.C.T. (Tissue-Tek) for freezing, sectioned (10–30 μm) using a cryostat (Leica), mounted onto SuperFrost Plus glass slides (Thermo Fisher Scientific) and stored at −80 °C. Sections were thawed to RT, rehydrated in DPBS for 10 min, and blocked for 1 h in DPBS containing 0.3% Triton X and 2–5% normal goat serum or 3% BSA (blocking buffer). Sections were stained (all at 1:400 in blocking buffer, unless otherwise stated) with a combination of antibodies (listed in Supplementary Table 4), AF555-conjugated phalloidin (Fisher Scientific), DAPI and Hoechst (Thermo Fisher Scientific) for 1 h at RT. For certain tumor samples, sections were also incubated with anti-mouse CD103, rabbit polyclonal anti-RFP and/or rabbit anti-mouse CD64/FcγRI overnight at 4 °C before secondary staining with anti-goat and anti-rabbit IgG antibodies for 1 h at RT (as detailed in Supplementary Table 4). Slides were washed and mounted using Prolong Diamond Antifade Mountant (Fisher Scientific) and analyzed as above or using a Phenoimager Fusion (Akoya Biosciences).

Fiji (Image J) was used to generate figures. For some tumors, Imaris software was used to segment cells and quantify their location in tumors. Briefly, cDC1, non-cDC1 APC and necrotic cell surfaces were generated based on MHC-II and CD103 or IgG2a staining and by smoothing and thresholding the data after background subtraction. Distances of cDC1s and cDC2s to necrotic areas were generated using the CytoMAP platform^63^ with the ‘Calculate distance’ function on the MATLAB GUI. For quantification of necrotic surfaces using Fc–DNGR-1 staining in tumors, images were blurred using a Gaussian blur filter, and a threshold was applied for image binarization in Fiji. Resulting surfaces were analyzed in Fiji, and the total necrotic area was generated by summing the areas of individual surfaces.

For high-dimensional confocal microscopy using the MACSima Imaging System (Miltenyi), 10-μm sections were prepared as above and prestained with DAPI before iterative staining with FITC- or AF488-, PE-, and APC- or AF647-conjugated anti-mouse antibodies as listed in Supplementary Table 4. Image acquisition and processing was performed on the MACSima instrument. Visualization and figure generation was performed using the MACS iQ View software.

For analysis of actin cytoskeleton binding, HeLa cells were fixed, permeabilized and blocked as above. Cells were stained with Hoechst (2.5 μg ml^−1^), phalloidin-AF647 (1:400) and anti-F-actin IgG2a (32 nM) for 1 h in 500 μl 2% BSA in DPBS. Samples were washed three times, stained with anti-mouse IgG2a-AF488 for 1 h in 2% BSA in DPBS, washed, mounted and imaged as above.

### Live cell imaging

Primary cDC1s or non-cDC1s (1 × 10^5^) from *Clec9a*^*Cre*^*Rosa*^*LSLtdTomato*^ mice were cultured in μ-Slide 8 Well^high^ Glass Bottom coverslips (Ibidi) in R10^+^ medium supplemented with 30% conditioned medium from differentiation cultures. Then, 2:1 CT-DR-labeled necrotic cells and 1 μg ml^−1^ Fc–DNGR-1 were added to cDC cultures, and coverslips were placed into the imaging chamber of an Olympus CSU-W1 Spinning Disk confocal microscope. Cells were maintained at 37 °C and 5% CO_2_ for the duration of the acquisition. Images were acquired every 2 min for 3 h. Videos were analyzed in ImageJ or Imaris v.9.1.2.

### Phagosomal rupture

RAW264.7 lines were plated onto an eight-well Ibidi μ-dish and left to adhere overnight. Cells were then washed with serum-free RPMI 1640 medium and left untreated or treated with titrations of DPI (D2926, Sigma-Aldrich) or R406 (S2194, Selleckchem) for 30 min in serum-free RPMI 1640 medium. Cells were then challenged with OVA beads or IgG-OVA beads for 3 h. Cells were then fixed with 4% PFA in DPBS for 20 min and quenched with three washes with 50 mM ammonium chloride in DPBS at RT. Outside beads were labeled with α-rabbit-AF405 in DPBS for 30 min and subsequently washed three times with DPBS. Cells were then permeabilized with 0.4% Triton X-100 in DPBS for 15 min and washed three times with DPBS. Afterward, the cells were blocked with 2% low fat milk in DPBS and incubated with 1:500 anti-mouse galectin-3-AF647 (clone M3/38, BioLegend) in 2% low fat milk in DPBS for 1 h at RT. Cells were then washed three times with DPBS and left in DPBS for imaging. Images were acquired on a Leica SP8 confocal microscope.

### In vivo tumor experiments

Tumor cell lines were dissociated with 0.25% trypsin, washed three times in DPBS and counted. Cells were resuspended and diluted in endotoxin-free DPBS (0.2–0.5 × 10^6^ cells per 100 μl) and injected s.c. in the shaved right flank of recipient mice. Tumor growth was monitored every 2–3 days, and the longest tumor diameter and perpendicular width were measured using digital Vernier calipers (tumor volume = length × width^2^/2). On day 4–6, mice were assigned to treatment groups to equalize tumor volume before treatment initiation. Mice in which tumors failed to implant by this time were excluded from downstream analysis and immediately terminated. Data collection and analysis were not performed blind to the conditions of the experiments.

Fc–DNGR-1 was administered via intratumoral injection (50–200 μg in 50 μl DPBS) every 3 days starting on day 6 or 7 for 2–3 total injections, unless stated otherwise in figure legends. Some mice were also injected with 2.5 mg kg^−1^ doxorubicin (MCA205, Merck Life Science), 2.5 mg kg^−1^ oxaliplatin (CT26, Merck Life Science UK) or control buffer (DPBS (doxorubicin) or 5% glucose (oxaliplatin)) i.t. Some mice were subjected to laser or CT-guided 10-Gy single-dose X-ray irradiation delivered in an irradiator cabinet on day 6 or 7 after tumor cell injection (Xstrahl RS320 Research System or SARRP). Mice received reversible general anesthesia throughout the duration of the procedure using a either ‘sleep mix’ of 0.05 mg kg^−1^ fentanyl, 5 mg kg^−1^ midazolam and 0.5 mg kg^−1^ medetomidine, reversed with a ‘wake mix’ of 1.2 mg kg^−1^ naloxone, 0.5 mg kg^−1^ flumazenil and 2.5 mg kg^−1^ atipam (RS320 Research System) administered intraperitoneally (i.p.) and dosed according to weight per mouse; or isoflurane (SARRP). Some experiments were performed in *Batf3*^*−/−*^ mice, or in WT C57BL/6 mice treated i.p. with 300 μg isotype or anti-CD8α IgG (BioXCell). The maximal permitted tumor size of 15 mm average diameter was not exceeded in any mouse.

### Fc–DNGR-1 pharmacokinetics analysis

C57BL/6 mice were injected i.p. with 100 μg of Fc–DNGR-1, and blood was harvested between days 0 and 6 postinjection, and serum isolated using serum Z-Gel tubes (Sarstedt). Fc–DNGR-1 titer was calculated using a serum-compatible anti-DNGR-1 ELISA. Briefly, 96-well high-affinity Nunc MaxiSorp plates (Thermo Fisher Scientific) were coated overnight with rat anti-mouse DNGR-1 IgG (clone 42D2, 5 μg ml^−1^ in 0.1 M sodium bicarbonate buffer) before extensive washing with 0.05% Tween-20 in DPBS. Plates were blocked for 1 h with 3% FCS (blocking buffer), washed once more, and incubated with DPBS-diluted serum (1:100 and 1:1000) or Fc–DNGR-1 standard curve for 2 h. After washing, plates were incubated with biotin rat anti-mouse DNGR-1 IgG (clone 7H11, 1 μg ml^−1^) for 2 h before being developed with alkaline phosphatase, as above.

### Quantification and statistical analysis

All statistical analyses were performed using GraphPad Prism software version 8. Statistical significance between two groups was determined using paired or unpaired two-tailed Student’s *t*-test. Statistical analyses for two or more groups were done by one- or two-way analysis of variance (ANOVA) followed by Bonferroni (in vivo) or Tukey (in vitro) multiple-comparison post hoc correction. Data distribution was assumed to be normal, but this was not formally tested. Data are plotted as the mean (for *n* < 3), mean ± s.e.m. (for *n* ≥ 3) or median as indicated in the figure legends. In vitro experiments represent technical replicates unless otherwise stated in the figure legends. In vivo experiments represent biological replicates.

Further information on research design is available in the Nature Research Reporting Summary linked to this article.

### Reporting summary

Further information on research design is available in the Nature Portfolio Reporting Summary linked to this article.

## Supplementary information

Supplementary Information Supplementary Tables 1–5.

Reporting Summary

Peer Review File

Supplementary Video 1 Fc–DNGR-1 promotes necrophagy by primary cDC2s. Confocal microscopy of non-cDC1 (magenta) from *Clec9a*^*Cre*^*Rosa*^*LSLtdTomato*^ mice incubated with CT-DR-labeled necrotic tumor cells (green) for 3 h. Data are representative of three biologically independent experiments.

Supplementary Video 2 Fc–DNGR-1 promotes necrophagy by primary cDC1s. Confocal microscopy of XCR1-enriched cDC1 (magenta) from *Clec9a*^*Cre*^*Rosa*^*LSLtdTomato*^ mice incubated with CT-DR-labeled necrotic tumor cells (green) for 3 h. Data are representative of three biologically independent experiments.

## Source data

Statistical Source Data for all Figures Statistical source data for all figures and Extended Data figures.

Unprocessed Gel ED Fig. 5b Unprocessed gel for Extended Data Fig. 5b.

## Data Availability

The human pan-cancer scRNA-seq myeloid cell atlas was derived as previously outlined^26^ from public datasets listed in Supplementary Table 3 and available via GitHub (https://github.com/nikita-rosendahl/cancermyeloidatlas). Source data are provided with this paper. All data supporting the fundings of this study are available within the article, its supplementary information, and Source data and/or available upon request from the corresponding author.

## Code availability

Code associated with generation of the pan-cancer single-cell myeloid atlas is available via GitHub (https://github.com/nikita-rosendahl/cancermyeloidatlas).
