# Supplementary material for: Coupling dead cell recognition to Fcγ receptors augments anticancer immunity
Source: Nat Cancer. 2026 May 20;7(9):1350–67. doi: 10.1038/s43018-026-01168-5 (PMC13600936; doi:10.1038/s43018-026-01168-5)
Supplement: Supplementary file 1 [file 43018_2026_1168_MOESM1_ESM.pdf]

nature cancer

Supplementary information https://doi.org/10.1038/s43018-026-01168-5

Coupling dead cell recognition to Fcγ receptors augments anticancer immunity

In the format provided by the authors and unedited

		Spleen						Tumour						
		FMO	1	2	3	4	5	FMO	1	2	3	4	5	6
cDC1	FcγRI	502	635	662	698	784	791	192	455	395	381	374	419	409
	FcγRIII	624	689	733	711	704	747	32.1	121	161	112	135	88	118
	FcγRIV	104	345	279	424	353	375	133	342	422	257	278	171	225
	FcγRIIB	622	905	1118	908	1137	1159	16.6	587	573	456	462	413	483
cDC2	FcγRI	202	243	253	288	360	360	25.8	1400	1587	1298	1232	1751	1832
	FcγRIII	595	588	643	633	635	639	108	743	1116	611	552	447	606
	FcγRIV	15	101	83.9	123	102	99.9	52.6	876	910	783	867	963	567
	FcγRIIB	610	755	1009	761	923	858	114	2597	4557	1874	1348	1279	1600
Mono/MC	FcγRI	156	353	420	412	555	523	159	18057	15134	15768	13779	14104	18926
	FcγRIII	221	497	561	561	572	559	287	4051	4339	3552	3352	2395	3516
	FcγRIV	-41.5	37.9	29.4	35.1	47.5	19.9	67.8	7488	4967	6311	7870	9916	6105
	FcγRIIB	387	803	958	808	1006	950	321	26723	33364	20881	14066	13186	16614

Supplementary Table 1. FcγR expression by spleen and tumour APCs. Geometric mean fluorescence intensity (GMFI) values for fluorescence-minus-one (FMO) controls and anti-FcγR antibody stains. Each numbered column represents an individual mouse. Data used for ∆GMFI calculations in Extended Data Figure 2b.

		Day 5						Day 12					
		FMO	1	2	3	4	5	FMO	1	2	3	4	5
MCA205	FcγRI	111	3724	1544	6785	6741	-	606	7644	8989	8674	8275	5637
	FcγRIII	202	1662	1193	2312	2379	-	280	2239	2158	1963	1782	1652
	FcγRIV	114	1081	850	1376	1256	-	591	4222	2674	3723	2232	2846
	FcγRIIB	-142	17161	27781	21178	20710	-	368	46864	47114	49179	52587	46079
MC38	FcγRI	-	6875	8779	8572	4513	7094	-	9250	12383	6622	11581	9724
	FcγRIII	-	1590	2206	1979	1652	1777	-	2157	2787	1945	2609	1553
	FcγRIV	-	864	945	1128	899	1002	-	9605	11478	16413	13937	12430
	FcγRIIB	-	29444	38412	31838	25941	37894	-	43131	38155	18130	39681	36913
BrafV600E	FcγRI	-	3799	3482	4553	4701	-	-	7308	9864	7383	7290	-
	FcγRIII	-	1166	1132	1341	1415	-	-	2093	2204	1878	1932	-
	FcγRIV	-	664	696	785	593	-	-	11666	8885	12158	9312	-
	FcγRIIB	-	18696	17984	14150	21772	-	-	35570	33630	31195	37193	-

Supplementary Table 2. FcγR expression on tumour MCs across timepoints. GMFI values for FMO controls and anti-FcγR antibody stains on $CD11c^{+}$ MHC-II^{+} $Ly6C^{lo}$ $CD88^{+}$ MCs across early and late tumour models. Each numbered column represents an individual mouse. Data used for ∆GMFI calculations in Extended Data Figure 2d.

Accession numbers/Dataset ID
GSE184880
GSE213243
GSE217517
GSE180661
PRJCA005422
GSE200218
GSE215120
PRJNA907381
GSE161529
GSE176078
GSE195861
GSE199515
GSE225600
GSE162498
GSE131907
GSE154826
PMID32561858
GSE112271
GSE189903
GSE162025
GSE139324
GSE164690
GSE173468
GSE188737
GSE234933
GSE154778
GSE156405
GSE197177
GSE214295
GSE231535
GSE183916
GSE167297
GSE234129
GSE223063
GSE224090
GSE235676

Supplementary Table 3. List of datasets included in single cell atlas of myeloid APCs in human cancer. GSE accession numbers are from Gene Expression Omnibus (GEO). PRJ numbers are from Chinese National Genomics Data Center. PMID numbers are from PubMed ID.

Anti-mouse antibodies used for flow cytometry				
**Target**	**Clone**	**Supplier**	**Catalogue number**	**Dilution**
CD45.2	104	BD Biosciences	741516	1:200
MHC-II (I-A/I-E)	M5/114.15.2	Thermo Fisher Scientific	25-5321-82	1:400
CD11c	N418	Thermo Fisher Scientific	368-0114-82	1:200
CD11b	M1/70	BD Biosciences	565976	1:200
CD115	AFS98	Biolegend	135517	1:200
CD117	2B8	Biolegend	105824	1:200
CD3e	145-2C11	Thermo Fisher Scientific	48-0031-82	1:200
CD4	GK1.5	Biolegend	100451	1:200
CD8a	53-6.7	Biolegend	100753	1:200
CD25	PC61	Biolegend	102006	1:200
CD44	IM7	Thermo Fisher Scientific	47-0441-82	1:200
CD62L	MEL-14	Biolegend	104412	1:200
CD69	H1.2F3	BD Biosciences	553237	1:200
PD-1	29F.1A12	Biolegend	135225	1:200
TIM-3	RMT3-23	Biolegend	119716	1:200
CD19	1D3/CD19	Biolegend	152431	1:200
B220	RA3-6B2	Biolegend	103240	1:200
DNGR-1	1F6	Biolegend	Custom	1:200
CD88	20/70	Biolegend	135808	1:200
Ly6C	HK1.4	Biolegend	128005	1:200
CD172a	P84	Biolegend	144028	1:200
CD301b	URA-1	Biolegend	146809	1:200
XCR-1	ZET	Biolegend	148220	1:200
F4/80	T45-2342	BD Biosciences	749284	1:200
FcγRI/CD64	X54-5/7.1	Biolegend	139323	1:200
FcγRIIB/CD32b	AT130-2	Thermo Fisher Scientific	12-0321-80	1:200
FcγRIII/CD16	S17014E	Biolegend	158009	1:100
FcγRIV/CD16.2	9E9	Biolegend	149505	1:200
CLEC10A	LOM-14	Biolegend	145704	1:200
IgG	N/A	Thermo Fisher Scientific	A21236	1:400
IgG2a	N/A	Thermo Fisher Scientific	A21241	1:400
**Anti-human antibodies used for flow cytometry**				
**Target**	**Clone**	**Supplier**	**Catalogue number**	**Dilution**
HLA-DR	L243	Biolegend	307636	1:200
CD14	HCD14	Biolegend	325639	1:200
CD11c	Bu15	Biolegend	337215	1:200
CD86	IT2.2	Biolegend	305439	1:200

FcγRI/CD64	10.1	BD Biosciences	562872	1:100
FcγRIIA/CD32a	IV.3	STEMCELL Technologies	60012FI.1	1:50
FcγRIIB/CD32b	S18005H	Biolegend	398304	1:100
FcγRIIIA/B/CD16	3G8	BD Biosciences	556619	1:100
**Anti-mouse antibodies used for confocal microscopy**				
**Target**	**Clone**	**Supplier**	**Catalogue number**	**Dilution**
IgG2a	N/A	Thermo Fisher Scientific	A21241	1:400
MHC-II (I-A/I-E)	M5/114.15.2	Thermo Fisher Scientific	11-5321-82	1:100
CD11b	M1/70	Biolegend	101205	1:50
CD11c	N418	Biolegend	117314	1:50
CD31	MEC13.3	Biolegend	102515	1:50
CD45	30-F11	Biolegend	103124	1:50
CD88	20/70	Biolegend	135808	1:50
CD103	AF1990	R&D Systems	AF1990	1:50
CD172a	P84	Biolegend	144027	1:50
B220	RA3-6B2	BD Biosciences	103205	1:50
XCR-1	ZET	Biolegend	148203	1:50
FcγRI/CD64	027	Sino	50086-R027	1:1000
Galectin-3	M3/38	Biolegend	125408	1:400
**Other antibodies used for confocal microscopy/flow cytometry**				
**Target**	**Clone**	**Supplier**	**Catalogue number**	**Dilution**
Rabbit anti-RFP	N/A	Rockland Inc.	600-401-379	1:500
Donkey anti-goat IgG (H + L)	N/A	Thermo Fisher Scientific	A32814	1:400
Donkey anti-rabbit IgG	N/A	Thermo Fisher Scientific	A48258	1:400
Donkey anti-rabbit IgG (H+L)	N/A	Thermo Fisher Scientific	A21207	1:400
FLAG	M2	Sigma Aldrich	A9594	1:100

Supplementary Table 4. Commercial antibodies used for flow cytometry and confocal microscopy.

**Gene**	**Reference**	**Sequence**
Fcgr1	Mm.Cas9.FCGR1.1.AA	GATCTTGCTGCCCACGTAGA
	Mm.Cas9.FCGR1.1.AB	TGGCCTTGAGGTGTCACGGA
Fcgr2b	Mm.Cas9.FCGR2B.1.AA	ACGGTGACACTGACATGCGA
	Mm.Cas9.FCGR2B.1.AB	ACGTGTAGCTGGCTTGGACC
Fcgr4	Mm.Cas9.FCGR4.1.AA	AAATAGGTGACCTTCCGTAC
	Mm.Cas9.FCGR4.1.AB	TTGCACTGGGTCACTGATCG

Supplementary Table 5. sgRNA sequences used for CRISPR-Cas9 gene editing.
