# Supplementary material for: Coupling dead cell recognition to Fcγ receptors augments anticancer immunity
Source: Nat Cancer. 2026 May 20;7(9):1350–67. doi: 10.1038/s43018-026-01168-5 (PMC13600936; doi:10.1038/s43018-026-01168-5)
Supplement: Supplementary file 2 [file 43018_2026_1168_MOESM2_ESM.pdf]

nature portfolio

Corresponding author(s): Caetano Reis e Sousa, Tomás Castro-Dopico

Last updated by author(s): Mar 30, 2026

Reporting Summary

Nature Portfolio wishes to improve the reproducibility of the work that we publish. This form provides structure for consistency and transparency in reporting. For further information on Nature Portfolio policies, see our Editorial Policies and the Editorial Policy Checklist.

Statistics

For all statistical analyses, confirm that the following items are present in the figure legend, table legend, main text, or Methods section.

n/a	Confirmed	
☐	☒	The exact sample size (*n*) for each experimental group/condition, given as a discrete number and unit of measurement
☐	☒	A statement on whether measurements were taken from distinct samples or whether the same sample was measured repeatedly
☐	☒	The statistical test(s) used AND whether they are one- or two-sided<br>*Only common tests should be described solely by name; describe more complex techniques in the Methods section.*
☒	☐	A description of all covariates tested
☐	☒	A description of any assumptions or corrections, such as tests of normality and adjustment for multiple comparisons
☐	☒	A full description of the statistical parameters including central tendency (e.g. means) or other basic estimates (e.g. regression coefficient) AND variation (e.g. standard deviation) or associated estimates of uncertainty (e.g. confidence intervals)
☐	☒	For null hypothesis testing, the test statistic (e.g. *F*, *t*, *r*) with confidence intervals, effect sizes, degrees of freedom and *P* value noted<br>*Give P values as exact values whenever suitable.*
☒	☐	For Bayesian analysis, information on the choice of priors and Markov chain Monte Carlo settings
☒	☐	For hierarchical and complex designs, identification of the appropriate level for tests and full reporting of outcomes
☒	☐	Estimates of effect sizes (e.g. Cohen's *d*, Pearson's *r*), indicating how they were calculated

Our web collection on statistics for biologists contains articles on many of the points above.

Software and code

Policy information about availability of computer code

Data collection	Flow cytometry: BD FACS Diva software version 8.0.1. Microscopy: Zen software version 2.3 software. ELISA absorbance measurements: SoftMax Pro version 6.1. Gel acquisition: ImageQuant 800 control software version 2.0.0.
Data analysis	Confocal microscopy: FIJI software, Imaris software version 9.5.1, or MACS iQ View software. Flow cytometry: FlowJo version 10. Single cell RNA sequencing analysis: R. Data were processed, plotted, and analysed in GraphPad Prism software version 8-10.

For manuscripts utilizing custom algorithms or software that are central to the research but not yet described in published literature, software must be made available to editors and reviewers. We strongly encourage code deposition in a community repository (e.g. GitHub). See the Nature Portfolio guidelines for submitting code & software for further information.

Data

Policy information about availability of data

All manuscripts must include a data availability statement. This statement should provide the following information, where applicable:

- Accession codes, unique identifiers, or web links for publicly available datasets
- A description of any restrictions on data availability
- For clinical datasets or third party data, please ensure that the statement adheres to our policy

The human pan-cancer single cell RNA sequencing myeloid cell atlas was derived as previously outlined [26] from public datasets listed in Supplementary Table 3

and available at https://github.com/nikita-rosendahl/cancermyeloidatlas. Source Data are provided with this paper. All data supporting the fundings of this study are available within the article, its supplementary information, and Source Data and/or available upon request from the corresponding.

Research involving human participants, their data, or biological material

Policy information about studies with human participants or human data. See also policy information about sex, gender (identity/presentation), and sexual orientation and race, ethnicity and racism.

Reporting on sex and gender	Healthy adult volunteers were anonymised, including sex/gender and this was not deemed relevant for this study.
Reporting on race, ethnicity, or other socially relevant groupings	Healthy adult volunteers were anonymised, including race/ethnicity and this was not deemed relevant for this study.
Population characteristics	N/A
Recruitment	Blood was obtained from anonymous healthy adult volunteers who had given informed consent.
Ethics oversight	The study was performed according to approved protocols of the ethics board of the Francis Crick Institute and the Human Tissue Act (study reference CREC_2024_006).

Note that full information on the approval of the study protocol must also be provided in the manuscript.

Field-specific reporting

Please select the one below that is the best fit for your research. If you are not sure, read the appropriate sections before making your selection.

☒ Life sciences ☐ Behavioural & social sciences ☐ Ecological, evolutionary & environmental sciences

For a reference copy of the document with all sections, see nature.com/documents/nr-reporting-summary-flat.pdf

Life sciences study design

All studies must disclose on these points even when the disclosure is negative.

Sample size	No statistical methods were used to pre-determine sample sizes, but these were similar to those reported in previous publications. Tumour experiments were initiated with n = 8-10 mice per group.
Data exclusions	No data were excluded.
Replication	Data were replicated a minimum of 2 times to ensure reproducibility, unless otherwise stated.
Randomization	On day 4-6, mice were assigned to treatment groups to equalise tumour volume prior to treatment initiation.
Blinding	Data collection and analysis were not performed blind to the conditions of the experiments due to a limited researcher numbers.

Reporting for specific materials, systems and methods

We require information from authors about some types of materials, experimental systems and methods used in many studies. Here, indicate whether each material, system or method listed is relevant to your study. If you are not sure if a list item applies to your research, read the appropriate section before selecting a response.

Materials & experimental systems

n/a	Involved in the study	
☐	☒	Antibodies
☐	☒	Eukaryotic cell lines
☒	☐	Palaeontology and archaeology
☐	☒	Animals and other organisms
☒	☐	Clinical data
☒	☐	Dual use research of concern
☒	☐	Plants

Methods

n/a	Involved in the study	
☒	☐	ChIP-seq
☐	☒	Flow cytometry
☒	☐	MRI-based neuroimaging

Antibodies

Antibodies used

Antibody information is supplied in Supplementary Table 4.
Anti-mouse antibodies used for flow cytometry (target, clone, supplier, catalogue number, and dilution):

CD45.2, 104, BD Biosciences, 741516, 1:200.
MHC-II (I-A/I-E), M5/114.15.2, Thermo Fisher Scientific, 25-5321-82, 1:400.
CD11c, N418, Thermo Fisher Scientific, 368-0114-82, 1:200.
CD11b, M1/70, BD Biosciences, 565976, 1:200.
CD115, AFS98, Biolegend, 135517, 1:200.
CD117, 2B8, Biolegend, 105824, 1:200.
CD3e, 145-2C11, Thermo Fisher Scientific, 48-0031-82, 1:200.
CD4, GK1.5, Biolegend, 100451, 1:200.
CD8a, 53-6.7, Biolegend, 100753, 1:200.
CD25, PC61, Biolegend, 102006, 1:200.
CD44, IM7, Thermo Fisher Scientific, 47-0441-82, 1:200.
CD62L, MEL-14, Biolegend, 104412, 1:200.
CD69, H1.2F3, BD Biosciences, 553237, 1:200.
PD-1, 29F.1A12, Biolegend, 135225, 1:200.
TIM-3, RMT3-23, Biolegend, 119716, 1:200.
CD19, 1D3/CD19, Biolegend, 152431, 1:200.
B220, RA3-6B2, Biolegend, 103240, 1:200.
DNGR-1, 1F6, Biolegend, Custom, 1:200.
CD88, 20/70, Biolegend, 135808, 1:200.
Ly6C, HK1.4, Biolegend, 128005, 1:200.
CD172a, P84, Biolegend, 144028, 1:200.
CD301b, URA-1, Biolegend, 146809, 1:200.
XCR-1, ZET, Biolegend, 148220, 1:200.
F4/80, T45-2342, BD Biosciences, 749284, 1:200.
FcγRI/CD64, X54-5/7.1, Biolegend, 139323, 1:200.
FcγRIIB/CD32b, AT130-2, Thermo Fisher Scientific, 12-0321-80, 1:200.
FcγRIII/CD16, S17014E, Biolegend, 158009, 1:100.
FcγRIV/CD16.2, 9E9, Biolegend, 149505, 1:200.
CLEC10A, LOM-14, Biolegend, 145704, 1:200.
IgG, N/A, Thermo Fisher Scientific, A21236, 1:400.
IgG2a, N/A, Thermo Fisher Scientific, A21241, 1:400.

Anti-human antibodies used for flow cytometry:

HLA-DR, L243, Biolegend, 307636, 1:200.
CD14, HCD14, Biolegend, 325639, 1:200.
CD11c, Bu15, Biolegend, 337215, 1:200.
CD86, IT2.2, Biolegend, 305439, 1:200.
FcγRI/CD64, 10.1, BD Biosciences, 562872, 1:100.
FcγRIIA/CD32a, IV.3, STEMCELL Technologies, 60012FI.1, 1:50.
FcγRIIB/CD32b, S18005H, Biolegend, 398304, 1:100.
FcγRIIIA/B/CD16, 3G8, BD Biosciences, 556619, 1:100.

Anti-mouse antibodies used for confocal microscopy:

IgG2a, N/A, Thermo Fisher Scientific, A21241, 1:400.
MHC-II (I-A/I-E), M5/114.15.2, Thermo Fisher Scientific, 11-5321-82, 1:100.
CD11b, M1/70, Biolegend, 101205, 1:50.
CD11c, N418, Biolegend, 117314, 1:50.
CD31, MEC13.3, Biolegend, 102515, 1:50.
CD45, 30-F11, Biolegend, 103124, 1:50.
CD88, 20/70, Biolegend, 135808, 1:50.
CD103, AF1990, R&D Systems, AF1990, 1:50.
CD172a, P84, Biolegend, 144027, 1:50.
B220, RA3-6B2, BD Biosciences, 103205, 1:50.
XCR-1, ZET, Biolegend, 148203, 1:50.
FcγRI/CD64, 027, Sino, 50086-R027, 1:1000.
Galectin-3, M3/38, Biolegend, 125408, 1:400.

Other antibodies used for confocal microscopy:

Rabbit anti-RFP, N/A, Rockland Inc., 600-401-379, 1:500.
Donkey anti-goat IgG (H + L), N/A, Thermo Fisher Scientific, A32814, 1:400.
Donkey anti-rabbit IgG, N/A, Thermo Fisher Scientific, A48258, 1:400.
Donkey anti-rabbit IgG (H+L), N/A, Thermo Fisher Scientific, A21207, 1:400.
FLAG, M2, Sigma Aldrich, A9594, 1:100.

Validation | Antibodies for flow cytometry and confocal microscopy were validated by commercial suppliers and using appropriate primary and/or secondary isotype controls as highlighted in manuscript Figures.

Eukaryotic cell lines

Policy information about cell lines and Sex and Gender in Research

Cell line source(s)	MutuDC were a kind gift from Hans Acha-Orbea (University of Lausanne, Switzerland). Braf 5555-V600E were a kind gift from George Kassiotis (The Francis Crick Institute, UK). Hoxb8-CDP were generated in-house, expanded, and deposited with the Francis Crick Institute Cell Science Science Technology Platform (FCI-CS-STP). All remaining cell lines were acquired from and maintained by the FCI-CS-STP.
Authentication	FCI-CS-STP authenticates all cell lines through short tandem repeat (STR) profiling using polymerase chain reaction.
Mycoplasma contamination	All cell lines used in this study were routinely tested from Mycoplasma contamination by FCI-CS-STP.
Commonly misidentified lines (See ICLAC register)	We did not use any of these lines.

Animals and other research organisms

Policy information about studies involving animals; ARRIVE guidelines recommended for reporting animal research, and Sex and Gender in Research

Laboratory animals	Wild type, Batf3-/-, Clec9a-CreRosaLSLtdTomato, Xcr1-Venus (kind gift from Tsuneyasu Kaisho (Wakayama Medical University, Japan)), and OT-I/Rag1-/- mice on a C57BL/6 background and wild-type BALB/c mice were bred and maintained at the Francis Crick Institute under specific-pathogen-free conditions, a 12 hr light/dark cycle (7 a.m. – 7 p.m.), and controlled room temperature (20-24oC) and humidity (55 % ± 10 %). Sex-matched female and male mice were used at 6-10 weeks of age for in vivo tumour experiments. Mice did not exceed the maximal permitted tumour size of 15 mm average diameter (mean of length and width). All animal experiments were performed in accordance with national and institutional guidelines for animal care and were approved by the Francis Crick Institute Biological Resources Facility Strategic Oversight Committee (incorporating the Animal Welfare and Ethical Review Body) and by the Home Office, United Kingdom (PP8593771).
Wild animals	No wild animals were used in this study.
Reporting on sex	In vivo tumour experiments and experiments using primary murine cells ex vivo were performed on sex-matched female and male mice.
Field-collected samples	No field-collected samples were used.
Ethics oversight	All animal experiments were performed in accordance with national and institutional guidelines for animal care and were approved by the Francis Crick Institute Biological Resources Facility Strategic Oversight Committee (incorporating the Animal Welfare and Ethical Review Body) and by the Home Office, United Kingdom.

Note that full information on the approval of the study protocol must also be provided in the manuscript.

Flow Cytometry

Plots

Confirm that:

☒ The axis labels state the marker and fluorochrome used (e.g. CD4-FITC).

☒ The axis scales are clearly visible. Include numbers along axes only for bottom left plot of group (a 'group' is an analysis of identical markers).

☒ All plots are contour plots with outliers or pseudocolor plots.

☒ A numerical value for number of cells or percentage (with statistics) is provided.

Methodology

Sample preparation

Tumours and spleens were excised at the indicated days after cell transplantation and mass determined using a microscale. For flow cytometry analysis, tissues were cut into pieces and digested with collagenase IV (200 U/ml, Worthington) and DNase I (100 µg/ml, Sigma Aldrich) for 30 min at 37ºC. Tissue was passed through a 70 µm cell strainer (Falcon), washed with FACS buffer (PBS with 1 % FCS and 2 mM EDTA), and pelleted by centrifugation at 400 x g for 4 min. For tumours, single cell suspensions were resuspended in 40 % Percoll and centrifuged at 600 x g for 20 min with minimal acceleration/deceleration.

Single cell suspensions from tumours, spleens, bone marrow-derived cells, and Hoxb8-cDC were stained with LIVE/DEAD Fixable Blue Dead Cell dye (Thermo Fisher Scientific) according to manufacturer's instructions and subsequently stained with fluorescently conjugated anti-mouse antibodies listed above or in Supplementary Table 4 for 30 min on ice. Cells were acquired live or fixed (Nordic-MUbio). For live cells, DAPI (2 ng/ml) was added at the point of acquisition for live/dead discrimination in certain instances. Quantification of total cell numbers by flow cytometry was performed using 123count

eBeads (Fisher Scientific).

For human FcγR analysis, MCs were harvested by incubation on ice (MCGM-CSF/IL-4) or with 1X TrypLE (Thermo Fisher Scientific), washed 2X in DPBS, and surface stained with anti-human antibodies as listed above.

For Fc-DNGR-1 opsonisation analysis, BRAFV600E mutant 5555 melanoma cells were irradiated with 240 mJ/cm2 UVC in PBS and cultured overnight in RPMI 1640 medium (lacking FCS). Necrotic tumour cells or FM-beads were then incubated with serial dilutions of Fc-fusion proteins at RT for 1 h in DPBS. Samples were washed 2X and incubated with secondary AF488-conjugated anti-mouse IgG (Thermo Fisher Scientific, 1:400) or anti-mouse DNGR-1 (clone 1F6, Biolegend, 1:100) at 4ºC for 30 min. Samples were washed and resuspended in DPBS before acquisition. For Annexin V staining, samples were washed and incubated with Annexin binding buffer and Annexin V AF647 conjugate (Invitrogen) for 10 min at RT, followed by incubation with DAPI prior to acquisition.

For phagocytosis assays, prior to irradiation, tumour cells were labelled with Cell Tracker-Deep Red (CT-DR) dye (Thermo Fisher Scientific; 1:1000 dilution) in DPBS containing Ca/Mg for 30 min at 37ºC. CT-DR-labelled necrotic cells were added to phagocytes at indicated ratios and timepoints. AF647-conjugated OVA-IC was prepared as above immediately prior to incubation with APC for 1 h at 37ºC. Following target incubation, murine phagocytes were surface stained for flow cytometry with a combination of the antibodies listed above. DAPI or LIVE/DEAD Fixable Blue Dead Cell Dye (Thermo Fisher Scientific) was used to exclude non-internalised necrotic cell material. Samples were acquired on an LSRFortessa or FACSymphony (BD Biosciences). Data were analysed using FlowJo software version 10. Necrophagic index integrates frequency and absolute quantity of phagocytosed material and was calculated using the following formula: (% CT-DR+ X CT-DR GMFI of CT-DR +)/10,000.

For priming of naive CD8 T cells, naïve CD8+ T cells from spleen and lymph nodes of OT-I/Rag1-/- mice were negatively selected using the EasySep mouse CD8+ T cell kit (STEMCELL Technologies) according to the manufacturer's instructions. T cells were then stained after 24 h in culture with OVA-necrotic cells, APCs and Fc-DNGR-1 with anti-mouse CD69 (clone H1.2F3, Biolegend), CD25 (clone PC61, Biolegend), and CD8a (clone 53-6.7, Biolegend) and samples were acquired on an LSRFortessa or FACSymphony (BD Biosciences). Data were analysed using FlowJo software version 10.

For activation of human myeloid cells, necrotic cells were incubated with human MCs at a 4:1 ratio for 24 h in the presence of AFA variants. After 24 h, human MCs were washed 3X in DPBS and surface stained for flow cytometry for 30 min on ice with anti-human antibodies listed above. LIVE/DEAD Fixable Blue Dead Cell Dye (Thermo Fisher Scientific) was used to exclude dead cells and samples were acquired on an LSRFortessa or FACSymphony (BD Biosciences). Data were analysed using FlowJo software version 10.

For DNGR-1 cross-blocking, necrotic cells were incubated with serial dilutions of Fc fusion proteins at RT for 1 h before addition of 0.5 µg/ml DNGR-1-FLAG for 30 min. Binding of proteins was detected using secondary monoclonal anti-FLAG M2-Cy3 (1:100, Sigma Aldrich) and Alexa Fluor 488-conjugated anti-mouse IgG (1:400; Thermo Fisher Scientific) at 4ºC for 30 min.

Instrument	For routine flow cytometry, samples were acquired on an LSRFortessa or FACSymphony (BD Biosciences). For FACS, sorting was performed on a FACSAria Fusion (BD Biosciences).
Software	Acquisition: BD FACS Diva software version 8.0.1. Analysis: FlowJo version 10.
Cell population abundance	Post-sorting fractions were confirmed to have > 90% purity by flow cytometry.
Gating strategy	SSC-A (log scale) vs FSC-A was used to initially gate on viable or necrotic cell populations based on size and granularity. FSC-A vs FSC-H was used to excluded doublets, and LIVE/DEAD staining to further discriminate between live and dead cells. Gating strategies for identifying relevant cell populations was determined from a combination of literature and prior experience, with stains validated with single colour controls. Gating strategies are shown in the paper and Extended Data Figures.

☒ Tick this box to confirm that a figure exemplifying the gating strategy is provided in the Supplementary Information.
