# Supplementary material for: Coupling dead cell recognition to Fcγ receptors augments anticancer immunity
Source: Nat Cancer. 2026 May 20;7(9):1350–67. doi: 10.1038/s43018-026-01168-5 (PMC13600936; doi:10.1038/s43018-026-01168-5)
Supplement: Supplementary file 3 [file 43018_2026_1168_MOESM3_ESM.pdf]

nature portfolio

Peer Review File

Coupling dead cell recognition to Fcγ receptors augments anti-cancer immunity

Corresponding Author: Professor Caetano Reis e Sousa

This manuscript has been previously reviewed at another journal. This document only contains information relating to versions considered at Nature Cancer.

This file contains all reviewer reports in order by version, followed by all author rebuttals in order by version.

Version 0:

Reviewer comments:

Reviewer #1

(Remarks to the Author)
The study by Castro-Dopico et al aims to address an important immunological question with significant translational implications: whether coupling F-actin sensing to FcR signaling can reprogram non-cDC1 APCs to acquire cross-presentation and anti-tumor capacity. While the overall study objective is conceptually compelling, the experimental narrative remains weakened by inconsistencies in FcR expression analysis, methodological confounds in receptor quantification, and insufficient reconciliation between receptor expression and functional signaling. After two rounds of rebuttal/revisions, the authors' explanations of FcR biology have become increasingly inconsistent and several concerns raised by the reviewers during the previous review rounds remain unresolved.

1. FcR expression analysis. A major limitation is the inconsistent characterization of FcR expression across DC and monocyte subsets and it is unclear how the presented data can be interpreted and their biological relevance is limited, given the discrepancy between mouse vs. human datasets.

i. In their response, the authors invoke a technical artifact as biological explanation stating that "The high expression in the tumour APCs requires experimental acquisition settings (i.e., reduced laser power, staining time, and antibody concentrations) that result in apparently low signal at low expression levels." This statement invalidates their quantitative comparison between spleen and tumor (shown in Fig. 6i of the manuscript, p. 18). If acquisition parameters differ, the resulting "increase" in tumor FcγR expression cannot be interpreted biologically. Yet the manuscript still claims (p18): "FcRI expression was upregulated in tumor DCs and monocyte relative to spleen counterparts". Thus, the instrument-setting artifact acknowledged in the rebuttal directly contradicts the quantitative claim retained in the paper. This discrepancy not only undermines the quantitative comparison but also invalidates any subsequent statistical inference (ΔGMI plots, histograms, or comparisons across tissues), as voltage or gain adjustments violate the assumption of comparable scaling.

ii. In the revised manuscript, the authors revised the axis to report expression as ΔGMI. This change is purely cosmetic, as without reporting raw FMO distributions, voltage setting, or calibration beads, ΔGMI does not ensure comparability across runs with different detector gains. ΔGMI is useful for qualitative gating but not for inter-sample quantification; when fluorescence gains differ, ΔGMI scales with photomultiplier settings rather than receptor density. The authors therefore fail to restore quantitative validity after admitting to altered acquisitions conditions. Importantly, there's no agreement between plotted ΔGMI data and the presented histograms. For example, tumor cDC1 appear positive (also higher expression compared to spleen) for FcRIIb, yet the ΔGMI plot indicates no change in expression.

iii. Although in the rebuttal the authors state that "For example, FcγRI (a.k.a. CD64) is well-known to be poorly expressed by steady-state DCs and used to discriminate cDCs from macrophages in healthy tissues[1]. This is further corroborated by our staining and Immgen data here. However, many of those studies show significant levels of FcγRI on putative cDC1s and cDC2s, often at higher levels than macrophages. This calls into question the accuracy of cell type identification in these settings.". However, the manuscript defines tumor "cDC2s" as $CD11b^+$ $CD172\alpha^+$ $MHCII^+$ $CD11c^+$ (Extended Data Fig. 1c–d, p. 38), which is precisely the phenotype overlapping monocyte-derived DCs and macrophages that Guilliams et al highlighted as ambiguous. The authors dismiss prior studies for contamination, while relying on equally permissive gating, a

self-contradictory rationale.

iv. Several of the arguments in the rebuttal and the manuscript are not supported by the presented data. For example, the authors claim that “These analyses confirm that cDC2s predominantly express low levels of FcγRIIB and RIV in healthy spleens, while splenic monocytes primarily express RIII and RIIB and lower levels of RI and RIV” (rebuttal page 1). But this is not true – in the provided histograms, splenic cDC2s appear to be negative for FcRIIB or FcRIV (or as positive as monocytes for FcRI), while monocytes do not primarily express RIII and RIIB. The differences are trivial (also not tested for statistical significance)(EDF2b).

2. Even if expression data are accurate, the manuscript still lacks substantial functional and translation relevance.

i. The functional experiments do not convincingly link specific FcR usage to necrophage or cross-presentation. While residual cross-presentation in FcRI-/- DCs (fig 3f) suggests redundancy though other FcRs (likely FcRIV), no experiments with double or triple KOs (or with blocking mAbs) were performed. Also, the authors infer FcRI involvement solely from partial inhibition and IFN-a dependence; however, no receptor-specific SYK phosphorylation or blocking antibody validation are shown.

ii. Although the authors discuss the limitations of the mouse system, there is significant discordance between the observed FcR expression profile/dynamics of expression in mice vs humans. The central claim of the study is that “cDCs express low FcR levels in the steady-state but this is overcome within the TME, where local signals boost expression.” Yet, all the presented human data clearly indicate that this is a mouse-specific phenomenon; Extended Data Figure 2 illustrates the comparable levels of FcR expression (primarily FcR2A and FcR2B, not FcRI, which the authors showed to be the dominant receptor in mice) between healthy tissue and metastatic tumors.

iii. Related to the point above, the rebuttal reframes the paper’s central hypothesis “The central objective of the study is that non-cDC1s are more abundant that cDC1s within tumours, locally express FcγRs, and therefore can engage IgG-coated targets within the tumour microenvironment. These criteria are fulfilled in both species. Human non-cDC1s already express notable levels of FcγRs at the steady-state, such as FcγRIIA and RIIB in human cDC2s.” First, in contrast to the authors’ argument, these criteria are not fulfilled in both species (in humans, cDC2s do not locally express FcRs). Second, while the study originally emphasized differential expression between spleen and tumor and functional reprogramming via FcR engagement, the authors now retreat to a weaker claim; FcγR expression merely “exists” and suffices for activity, while attributing differences to “local upregulation.” This conceptual reframing substantially weakens the manuscript’s original mechanistic claim and instead leaves only a descriptive observation of FcR presence, without mechanistic linkage to cross-presentation.

3. Additional methodological and conceptual limitations

• Lack of direct evidence for phagosomal rupture and SYK activation in primary DCs; all mechanistic data derive from macrophage lines.
• Gating strategies in tumor APCs remain ambiguous, and temporal regulation of FcR expression is untested.
• Human data measure activation but not cross-presentation, leaving the translational bridge incomplete.
• Potential confounding by circulating IgG, off-target FcR engagement, and inconsistent statistics are not addressed.
• Experiments were conducted in tumor models that are highly immunogenic. Demonstrating efficacy in poorly immunogenic or immune-cold tumors (e.g., B16F10) would be important. Similarly, the absence of synergy data with checkpoint blockade (anti-PD-1/PD-L1) is a missed opportunity.
• Lack of clarity in statistical presentation; many plots represent technical rather than biological replicates. Statistical tests are sometimes applied to pooled data without clarity on sample size or variance structure.

Reviewer #3

(Remarks to the Author)
The authors have employed a clever strategy to direct the capture of necrotic cells to cDC2 and monocyte-derived DCs (MCs) utilizing antibodies to bridge F-actin exposed on necrotic cells to Fc receptors. The authors show that this uptake of dead cells enables necrotic cell derived antigen cross-presentation by cDC2 to CD8+ T cells. The authors have provided a substantial body of in-vitro and in-vivo evidence to link dead cell associated antigen uptake by FcγRs to generate anti-tumor immunity. The work represents an important advance to the field and should be published without delay. Only a few minor comments are below.

In addition to antibody dependent cell cytotoxicity (ADCC) FcγR engagement has been associated to CD8+ T cell cytotoxicity. A prior report from 2002 (PMID: 12093890) has demonstrated the rationale behind FcR targeting in immunotherapy by administration of antitumor antibodies. The author’s idea of developing novel F-actin monoclonal antibodies is an important contribution. Can the authors include this paper in their reference list?

The authors repeatedly point out lower necrotic debris uptake efficiency in cDC2 DCs. Their confocal data however from Fig. 3d confirms otherwise. At basal level the %CT-DR is undoubtedly higher in cDC1 however, it is noticeably higher in Hoxb8-cDC2 after WT Fc-DNGR-1. Can the authors point this out clearly and clarify whether there is a difference between Necrophagic index (Fig. 3b) and CT-DR% (Fig. 3d)?

The idea that cDC2 DCs are CD4+ T cell activators is widely published in the field (PMID: 12093890, PMID: 2528589, PMID: 9892619). Although this might be outside the scope of the current manuscript, can the authors comment on this aspect in their discussion?
Different DC populations exhibit varying degrees of maturity inside a tumor set up PMID: 9892619). Can the authors add their thoughts on whether the type of FcγR engagement changes as a result?

Reviewer #4

(Remarks to the Author)
By further mining their dataset the authors appropriately answered my question on the expression of CD80 and CD86 across cDC1s, cDC2s, and classical monocytes in human tumours. It turned out that all APCs analysed expressed similar levels of co-stimulatory molecules within tumours. Moreover, AFA-induced necrotic cell uptake was found capable of induce CD86 upregulation on human MCs in vitro. Importantly, the authors thoroughly answered all the questions raised by Reviewers 1 and 3 via the inclusion of a large amount of convincing new data. In my view this thorough and original study deserves publication as such in NatCancer.

Minor
Lanes 102-104: the term ‘monocyte waterfall’ was coined first by Tamoutounour et al. (DOI: 10.1002/eji.201242847) and not the provided Ref. 27-28.

Version 1:

Reviewer comments:

Reviewer #3

(Remarks to the Author)
The authors have revised all the concerns. While the authors state that they have improved the response stating “These results highlight how targeting FcγRs on tumour APCs may be leveraged therapeutically to boost CD8+ T cell responses to tumour antigens across species”, this reviewer was unable to locate this statement in the revised text. The authors may have chosen to leave this statement out again to avoid the impression that the human cross-presentation assays were performed following Fc-DNGR1 or anti-F-actin Affimer Fc fusion proteins. In other words, the proof of anti-tumor immunity in human requires future work which the authors make clear in their discussion. The authors have taken care to indicate "mouse" in various parts of the text when describing the results to separate what was done on mouse vs human cells. It is prudent to do so and avoid making strong statements about CD8 T cell responses across species.

Open Access This Peer Review File is licensed under a Creative Commons Attribution 4.0 International License, which permits use, sharing, adaptation, distribution and reproduction in any medium or format, as long as you give appropriate credit to the original author(s) and the source, provide a link to the Creative Commons license, and indicate if changes were

made.
In cases where reviewers are anonymous, credit should be given to 'Anonymous Referee' and the source.
The images or other third party material in this Peer Review File are included in the article's Creative Commons license, unless indicated otherwise in a credit line to the material. If material is not included in the article's Creative Commons license and your intended use is not permitted by statutory regulation or exceeds the permitted use, you will need to obtain permission directly from the copyright holder.
To view a copy of this license, visit https://creativecommons.org/licenses/by/4.0/

Reviewer #1
Remarks to the Author

The study by Castro-Dopico et al aims to address an important immunological question with significant translational implications: whether coupling F-actin sensing to FcR signaling can reprogram non-cDC1 APCs to acquire cross-presentation and anti-tumor capacity. While the overall study objective is conceptually compelling, the experimental narrative remains weakened by inconsistencies in FcR expression analysis, methodological confounds in receptor quantification, and insufficient reconciliation between receptor expression and functional signaling. After two rounds of rebuttal/revisions, the authors' explanations of FcR biology have become increasingly inconsistent and several concerns raised by the reviewers during the previous review rounds remain unresolved.

1. FcR expression analysis. A major limitation is the inconsistent characterization of FcR expression across DC and monocyte subsets and it is unclear how the presented data can be interpreted and their biological relevance is limited, given the discrepancy between mouse vs. human datasets.

i. In their response, the authors invoke a technical artifact as biological explanation stating that "The high expression in the tumour APCs requires experimental acquisition settings (i.e., reduced laser power, staining time, and antibody concentrations) that result in apparently low signal at low expression levels." This statement invalidates their quantitative comparison between spleen and tumor (shown in Fig. 6i of the manuscript, p. 18). If acquisition parameters differ, the resulting "increase" in tumor FcγR expression cannot be interpreted biologically. Yet the manuscript still claims (p18): "FcRI expression was upregulated in tumor DCs and monocyte relative to spleen counterparts". Thus, the instrument-setting artifact acknowledged in the rebuttal directly contradicts the quantitative claim retained in the paper. This discrepancy not only undermines the quantitative comparison but also invalidates any subsequent statistical inference (ΔGMI plots, histograms, or comparisons across tissues), as voltage or gain adjustments violate the assumption of comparable scaling.

This is a misunderstanding by the reviewer, not "a technical artefact". To be clear: in our experiments, <u>the same acquisition parameters were used on tumours and spleen</u>. We now explicitly state in the Results "when identical acquisition parameters were used across tissues" and reiterate this in the Methods. Our statement in the rebuttal was intended to explain that when acquisition parameters are set based on signal intensity in tumour samples, where FcR expression level is very high, the corresponding signal from splenic cDCs acquired <u>with the same settings</u> appears very low. As an analogous example, in our previous rebuttal, we showed FcγR expression levels from Immgen datasets on different scales with or without comparison to inflammatory myeloid cells. To avoid misunderstanding, we now state "This contrasted with cDC2s from lymphoid organs (spleen or tumour-draining lymph nodes), in which FcγR expression remained low even in tumour-bearing mice when using identical acquisition parameters across tissues (Extended Data Fig. 2b, c)".

ii. In the revised manuscript, the authors revised the axis to report expression as ΔGMI. This change is purely cosmetic, as without reporting raw FMO distributions, voltage setting, or calibration beads, ΔGMI does not ensure comparability across runs with different detector gains. ΔGMI is useful for qualitative gating but not for inter-sample quantification; when fluorescence gains differ, ΔGMI scales with photomultiplier settings rather than receptor density. The authors therefore fail to restore quantitative validity after admitting to altered acquisitions conditions. Importantly, there's no agreement between plotted ΔGMI data and the presented histograms. For example, tumor cDC1 appear positive (also higher expression compared to spleen) for FcRIIb, yet the ΔGMI plot indicates no change in expression.

In our previous rebuttal, we did not "admit to altered acquisition conditions" - see above comment about <u>acquisition settings being the same across all samples</u>. The reviewer states there is no agreement between histograms and bar graphs, yet these represent the same dataset. Indeed, this serves to illustrate the importance of the ΔGMFI calculations incorporating FMOs, which can be different between tissues.

We now provide the raw GMFI data for Extended Data Figure 2 in Supplementary Tables, as reproduced below.

		Spleen						Tumour						
		FMO	1	2	3	4	5	FMO	1	2	3	4	5	6
cDC1	FcγRI	502	635	662	698	784	791	192	455	395	381	374	419	409
	FcγRIII	624	689	733	711	704	747	32.1	121	161	112	135	88	118
	FcγRIV	104	345	279	424	353	375	133	342	422	257	278	171	225
	FcγRIIB	622	905	1118	908	1137	1159	16.6	587	573	456	462	413	483
cDC2	FcγRI	202	243	253	288	360	360	25.8	1400	1587	1298	1232	1751	1832
	FcγRIII	595	588	643	633	635	639	108	743	1116	611	552	447	606
	FcγRIV	15	101	83.9	123	102	99.9	52.6	876	910	783	867	963	567
	FcγRIIB	610	755	1009	761	923	858	114	2597	4557	1874	1348	1279	1600
Mono/MC	FcγRI	156	353	420	412	555	523	159	18057	15134	15768	13779	14104	18926
	FcγRIII	221	497	561	561	572	559	287	4051	4339	3552	3352	2395	3516
	FcγRIV	-41.5	37.9	29.4	35.1	47.5	19.9	67.8	7488	4967	6311	7870	9916	6105
	FcγRIIB	387	803	958	808	1006	950	321	26723	33364	20881	14066	13186	16614

iii. Although in the rebuttal the authors state that "For example, FcγRI (a.k.a. CD64) is well-known to be poorly expressed by steady-state DCs and used to discriminate cDCs from macrophages in healthy tissues[1]. This is further corroborated by our staining and Immgen data here. However, many of those studies show significant levels of FcγRI on putative cDC1s and cDC2s, often at higher levels than macrophages. This calls into question the accuracy of cell type identification in these settings.". However, the manuscript defines tumor "cDC2s" as $CD11b^+$ $CD172\alpha^+$ $MHCII^+$ $CD11c^+$ (Extended Data Fig. 1c–d, p. 38), which is precisely the phenotype overlapping monocyte-derived DCs and macrophages that Guilliams et al highlighted as ambiguous. The authors dismiss prior studies for contamination, while relying on equally permissive gating, a self-contradictory rationale.

The reviewer is incorrect: we identify cDC2s as $CD11b^+$ $CD172\alpha^+$ $MHCII^+$ $CD11c^+$ cells that are negative for CD88. CD88 is a sensitive marker for identifying monocyte-derived cells within the $CD11c^+$ $MHC\text{-}II^+$ gate [1-3], allowing us to precisely discriminate between $CD88^+$ monocyte-derived cells (MCs, a.k.a monocyte-derived DCs) from bone fide $CD88^-$ cDCs. Indeed, this is shown in our gating strategy in Extended Data Fig. 1c (reproduced below), where we go even further and carry out a finer analysis of $CD88^+$ MCs based on Ly6C expression to discriminate MCs at different stages of differentiation from monocytes.

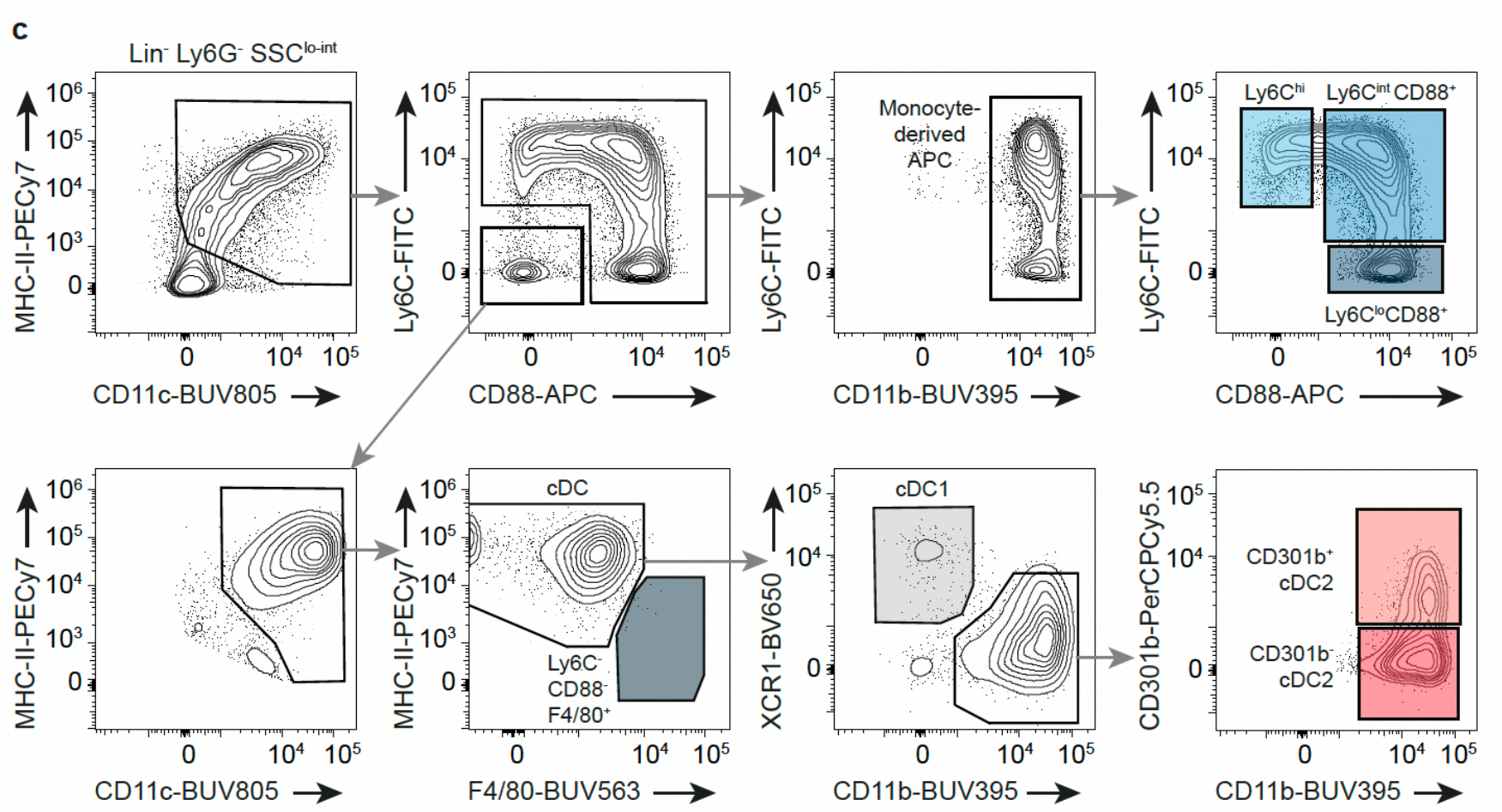

iv. Several of the arguments in the rebuttal and the manuscript are not supported by the presented data. For example, the authors claim that "These analyses confirm that cDC2s predominantly express low levels of FcγRIIB and RIV in healthy spleens, while splenic monocytes primarily express RIII and RIIB and lower levels of RI and RIV" (rebuttal page 1). But this is not true – in the provided histograms, splenic cDC2s appear to be negative for FcRIIB or FcRIV (or as positive as monocytes for FcRI), while monocytes do not primarily express RIII and RIIB. The differences are trivial (also not tested for statistical significance) (EDF2b).

Regarding cDC2s, our data from both flow cytometry and Immgen analysis show low FcγR expression levels in these cells in the spleen, especially in comparison to FcγR expression levels in tumour cDCs, as already discussed. However, it is not negative as suggested by the reviewer. Comparing across individual Fc receptors by flow cytometry is challenging as they are all stained with different antibodies. We have added additional lines across the y-axis that show that RIIB and RIV stain more intensely over background than RI and RIII (reproduced below from Extended Data Fig. 2b). The same applies to splenic monocytes. Our analysis shows that, when controlled for differences in background (FMO) between spleen and tumour, FcγRIII and RIIB show the strongest staining intensity in splenic monocytes. However, they do indeed express all Fc receptors and differences appear to be small, as might be expected. Once again, the apparent low expression is accentuated by comparison to tumour MCs. The reviewer suggests applying statistical tests between different Fc receptors, but this is erroneous due to the use of different antibodies and fluorophores for each receptor, making inter-receptor comparisons invalid.

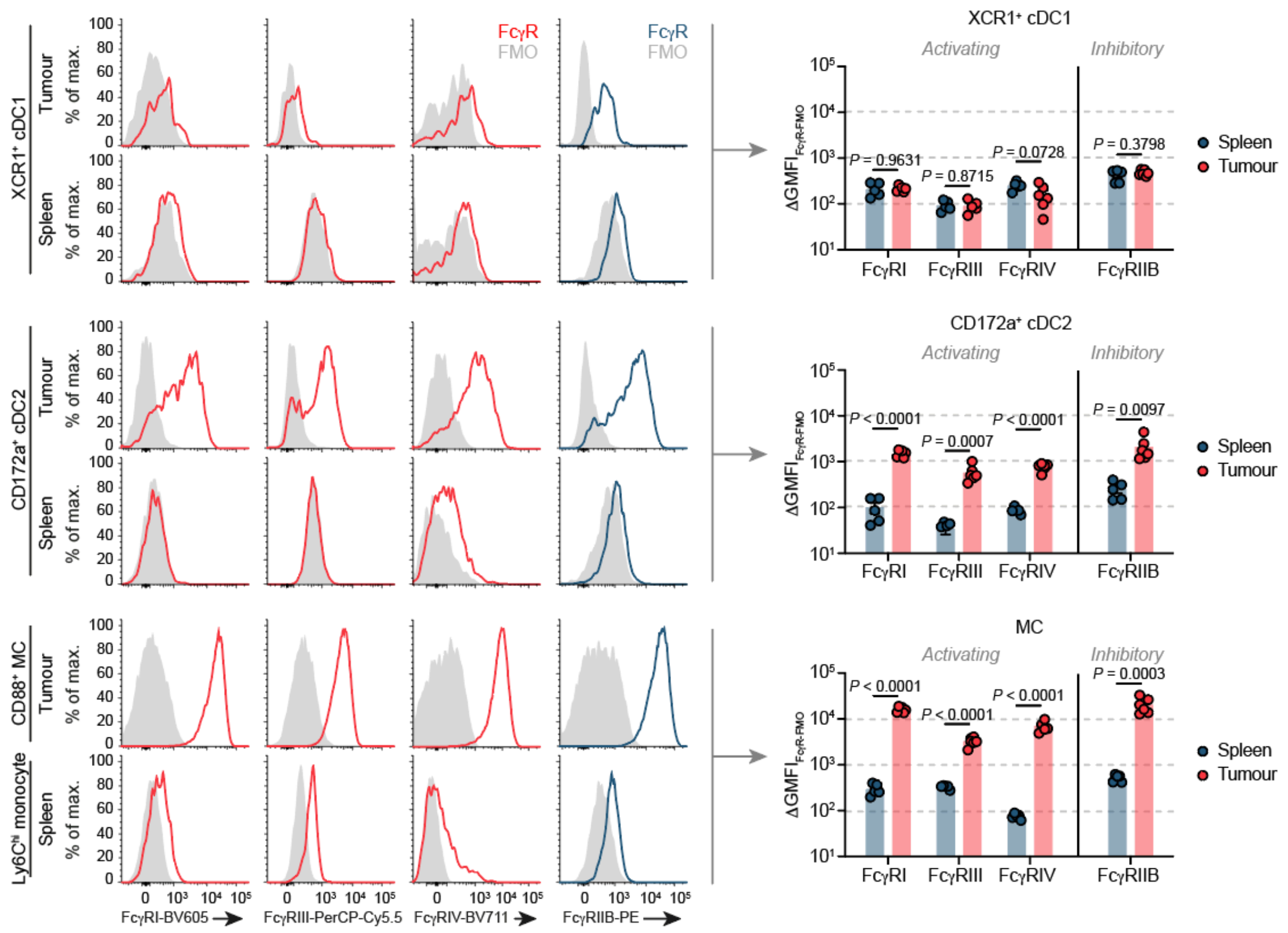

Importantly, all observations are strongly consistent in pattern and magnitude with Immgen data for analogous populations (reproduced below from Extended Data Fig. 2g), as discussed in previous rebuttals.

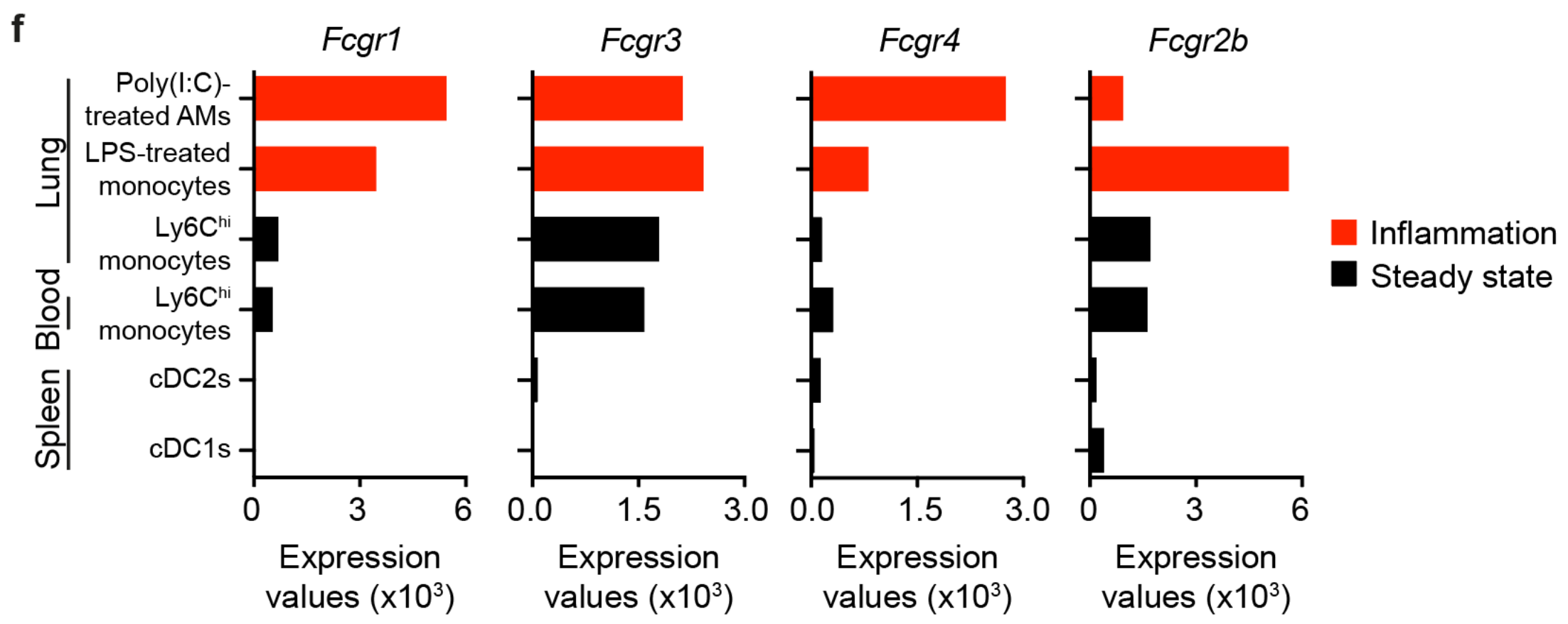

2. Even if expression data are accurate, the manuscript still lacks substantial functional and translation relevance.

i. The functional experiments do not convincingly link specific FcR usage to necrophage or cross-presentation. While residual cross-presentation in FcRI-/- DCs (fig 3f) suggests redundancy though other FcRs (likely FcRIV), no experiments with double or triple KOs (or with blocking mAbs) were performed. Also, the authors infer FcRI involvement solely from partial inhibition and IFN-a dependence; however, no receptor-specific SYK phosphorylation or blocking antibody validation are shown.

We disagree with this comment. We confirmed FcγRI KO in primary cDCs to be efficient and receptor-specific, leading to a complete loss of necrophagy and XP in cDC1s and a major defect in cDC2s. In contrast, FcγRIV KO cDCs, which is also effectively and specifically targeted, are not significantly impaired in necrophagy or cross-presentation. This is also the case with FcγRIIB KO cDCs. Given the residual activity in targeted cDC2s, we have revised our text to state "FcγRI was important for Fc-DNGR-1 enhancement of necrophagy in both cDC1s and cDC2s" rather than "essential". Of note, we performed double targeting of FcγRI and FcRIV in primary cDCs but have found that this results in reduced KO efficiency for each gene individually and substantial remaining expression of both FcγRI and RIV (see below for reference). However, combined with the results showing loss of necrophagy and XP when using N297A Fc-DNGR-1, our data support the conclusion that FcγRs are absolutely required for Fc-DNGR-1 function and that FcγRI plays an important role.

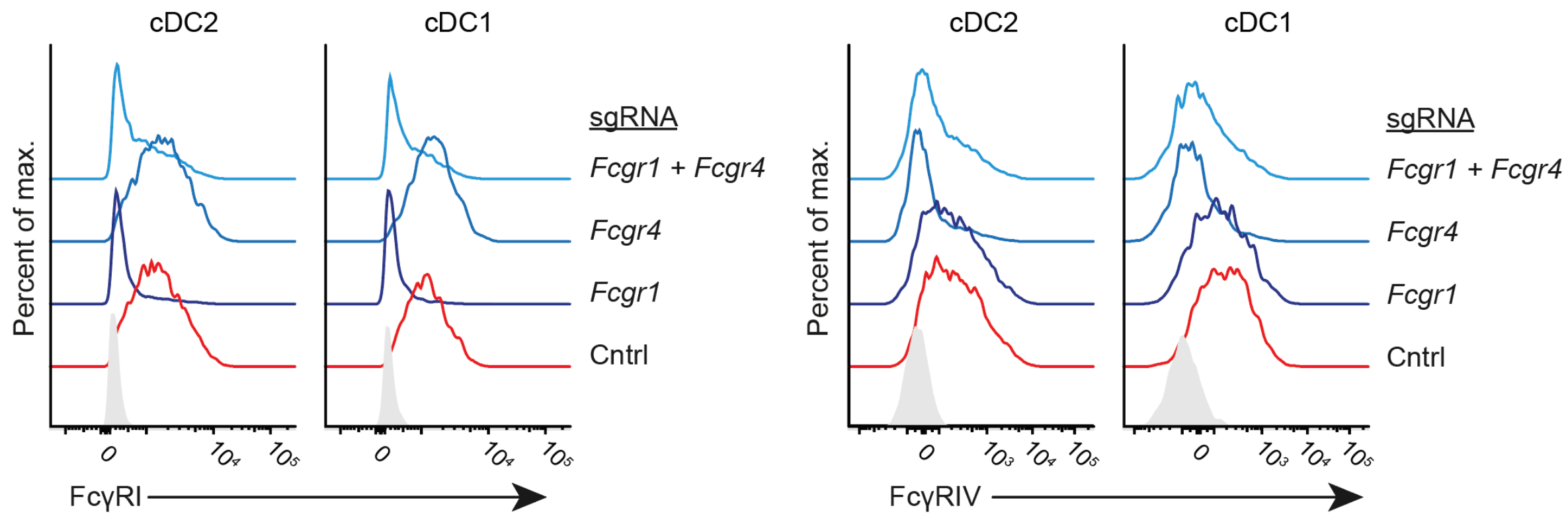

ii. Although the authors discuss the limitations of the mouse system, there is significant discordance between the observed FcR expression profile/dynamics of expression in mice vs humans. The central claim of the study is that "cDCs express low FcR levels in the steady-state but this is overcome within the TME, where local signals boost expression." Yet, all the presented human data

clearly indicate that this is a mouse-specific phenomenon; Extended Data Figure 2 illustrates the comparable levels of FcR expression (primarily FcR2A and FcR2B, not FcRI, which the authors showed to be the dominant receptor in mice) between healthy tissue and metastatic tumors.

As the reviewer points out, we have discussed differences between human and mouse FcγR expression and the limitations of mouse models, and have already significantly extended our original study to assess the functional role of AFAs with human cells (Figure 7). However, we disagree with the reviewer that the central claim of the study is that cDCs express low FcγR levels in steady state. In our original manuscript, we stated in our abstract: "Here we show that abundant tumour-associated APC, including cDC2 and monocyte-derived cells, express SYK-coupled Fcγ receptors (FcγR) and can cross-present IgG-bound proteins", which we show to be the case for both humans and mice. In the main text, we stated first that "FcγR transcript profiling revealed that both activating FcγRIIA and inhibitory FcγRIIB were expressed on human tumour-infiltrating CD1c$^+$ cDC2 at levels comparable to those in macrophages, with expression similar between tumour and adjacent healthy tissue". We then go on to state "In mice, in non-inflammatory conditions, cDCs typically express only low levels of FcγRs. This was indeed the case for splenic cDCs even in tumour-bearing mice. In contrast, within tumours, CD172a$^+$ cDC2s and CD88$^+$ MCs highly expressed all activating FcγR (FcγRI, RIII, and RIV), as well as inhibitory FcγRIIB, consistent with upregulation within the TME". Therefore, we have always noted the differences between humans and mice, summarising "FcγRs represent a tractable and species-conserved system that can couple IgG binding to inducible XP by abundant APC in tumours". These remarks remain largely unchanged in the current manuscript and across all versions, demonstrating that the framing of our manuscript has not changed as suggested. The premise has always been simply that Fcγ receptors are expressed by abundant APCs within tumours and can be targeted for therapeutic purposes. We agree that human cDC2s primarily express FcγRIIA and RIIB across all tissue sites but limited FcγRI, as discussed already in the manuscript (see above). Importantly, we go on to validate that FcγRIIA can perform similar functions in human APCs, when we prevent necrophagy in $MC_{GM\text{-}CSF/IL\text{-}4}$ using a blocking antibody to this receptor. This shows that Fc-DNGR-1 or AFA-mediated necrophagy and activation via FcγRs is conserved between species.

iii. Related to the point above, the rebuttal reframes the paper's central hypothesis "The central objective of the study is that non-cDC1s are more abundant that cDC1s within tumours, locally express FcγRs, and therefore can engage IgG-coated targets within the tumour microenvironment. These criteria are fulfilled in both species. Human non-cDC1s already express notable levels of FcγRs at the steady-state, such as FcγRIIA and RIIB in human cDC2s." First, in contrast to the authors' argument, these criteria are not fulfilled in both species (in humans, cDC2s do not locally express FcRs). Second, while the study originally emphasized differential expression between spleen and tumor and functional reprogramming via FcR engagement, the authors now retreat to a weaker claim; FcγR expression merely "exists" and suffices for activity, while attributing differences to "local upregulation." This conceptual reframing substantially weakens the manuscript's original mechanistic claim and instead leaves only a descriptive observation of FcR presence, without mechanistic linkage to cross-presentation.

We refute that a reframing of the paper's central hypothesis has taken place, as the statement quoted by the reviewer above from our rebuttal is present nearly verbatim in our original manuscript, as outlined above. To address the specific points: in our rebuttal, "local" simply referred to within the tumour microenvironment, irrespective of expression at other sites. To avoid misunderstanding about expression outside tumours, we have avoided use of this term in the manuscript. In reference to the last point, we draw the reviewer to experiments in Fig. 1 (reproduced below) where we mechanistically link Fc receptor expression by tumour APCs to uptake (Fig. 1g) and cross-presentation (Fig. 1h) of OVA-IC to CD8$^+$ T cells. These results highlight how targeting FcγRs on tumour APCs may be leveraged therapeutically to boost CD8$^+$ T cell responses to tumour antigens across species.

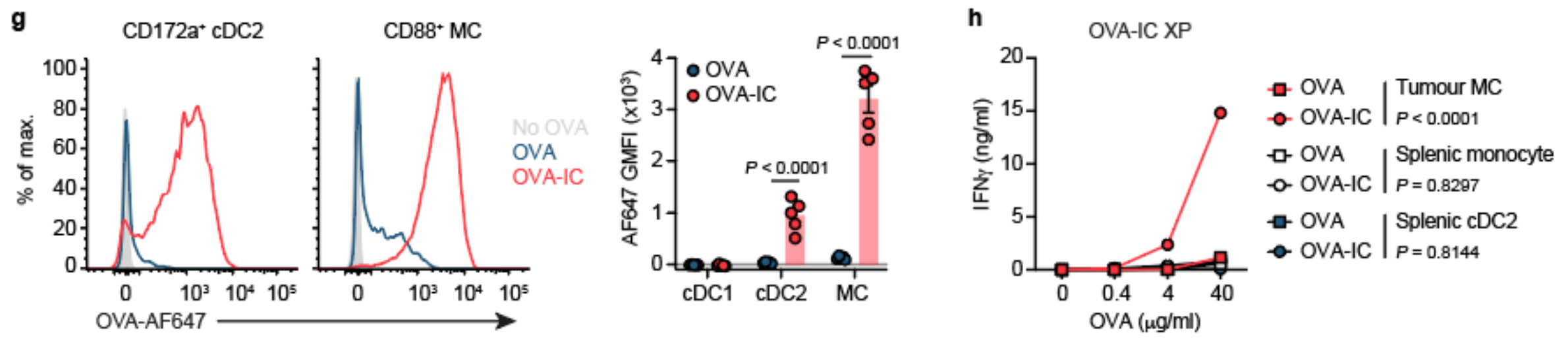

3. Additional methodological and conceptual limitations

• Lack of direct evidence for phagosomal rupture and SYK activation in primary DCs; all mechanistic data derive from macrophage lines.

This is a new comment by this reviewer that was addressed previously in response to another reviewer. Currently, assessment of phagosomal rupture is most tractable in RAW264.7 cell lines and we have explained why this has not been feasible in other cell types so far. We have now updated our manuscript to highlight these considerations more directly and to point readers to previous publications in which RAW264.7 cells have been used to investigate phagosomal rupture: "For this, we utilised RAW264.7 macrophages, a genetically tractable FcγR-expressing cell line that has been used previously to investigate the role of P2C in cross-presentation [4, 5].".

• Gating strategies in tumor APCs remain ambiguous, and temporal regulation of FcR expression is untested.

We have provided a thorough gating strategy for tumour APCs, as outlined in comment 1 (iii). We have assessed temporal regulation of FcγRs in tumour APCs, but this was not included in our original rebuttal due to space constraints. We now show that FcγR expression levels increase between incipient (day 5) and established (day 12) tumours, as exemplified for Ly6C^{lo} CD88^{+} MCs. This was most notable for FcγRIV and to a lesser extent for FcγRI and FcγRIIB, while FcγRIII remains largely unchanged across timepoints. This is reproduced below from a revised Extended Data Fig. 2d.

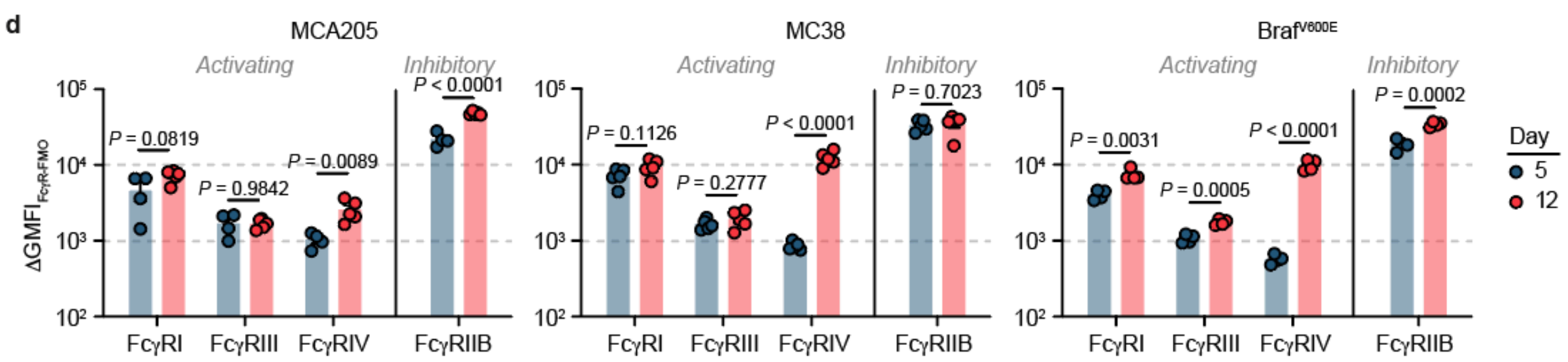

• Human data measure activation but not cross-presentation, leaving the translational bridge incomplete.

This is a new comment by this reviewer that was addressed previously in response to another reviewer. As stated in our discussion: "Importantly, how AFAs couple necrotic cell sensing to XP in human APCs is an area of ongoing investigation. It will require establishing an assay with sufficient sensitivity to detect XP of dead cell-associated antigen, which has eluded us thus far (data not shown).".

• Potential confounding by circulating IgG, off-target FcR engagement, and inconsistent statistics are not addressed.

This is a new comment by this reviewer that appears to allude to other reviewers' comments, which we have previously addressed. As outlined in replies to other reviewers, we provided updated statistical

analyses and provided mechanistic dissection of *in vivo* tumour efficacy using *Batf3* KO mice, anti-CD8 IgG-mediated T cell depletion, and LALA-PG Fc-DNGR-1.

• Experiments were conducted in tumor models that are highly immunogenic. Demonstrating efficacy in poorly immunogenic or immune-cold tumors (e.g., B16F10) would be important. Similarly, the absence of synergy data with checkpoint blockade (anti-PD-1/PD-L1) is a missed opportunity.

This is a new comment by this reviewer. We agree that functional assessment of Fc-DNGR-1 and AFAs in immune-cold tumours and synergy with immune checkpoint blockade is of great interest. We have now added a line to the Discussion to reflect this.

• Lack of clarity in statistical presentation; many plots represent technical rather than biological replicates. Statistical tests are sometimes applied to pooled data without clarity on sample size or variance structure.

We have stated in the figure legends all statistical tests performed and n numbers. One experiment in Extended Data Fig. 8a is derived from two pooled experiments, which is stated in the figure legend. Additionally, we have now included absolute *P* values in all figures and clarified the use of technical vs biological replicates in the Methods.

Reviewer #3
Remarks to the Author

The authors have employed a clever strategy to direct the capture of necrotic cells to cDC2 and monocyte-derived DCs (MCs) utilizing antibodies to bridge F-actin exposed on necrotic cells to Fcγ receptors. The authors show that this uptake of dead cells enables necrotic cell derived antigen cross-presentation by cDC2 to CD8+ T cells. The authors have provided a substantial body of in-vitro and in-vivo evidence to link dead cell associated antigen uptake by FcγRs to generate anti-tumor immunity. The work represents an important advance to the field and should be published without delay. Only a few minor comments are below.

We thank the reviewer for the positive appraisal of our study.

In addition to antibody dependent cell cytotoxicity (ADCC) FcγR engagement has been associated to CD8+ T cell cytotoxicity. A prior report from 2002 (PMID: 12093890) has demonstrated the rationale behind FcR targeting in immunotherapy by administration of antitumor antibodies. The author's idea of developing novel F-actin monoclonal antibodies is an important contribution. Can the authors include this paper in their reference list?

We agree with the reviewer that this is an important study and now reference this study in our Introduction: "Indeed, adoptive transfer of GM-CSF-elicited bone marrow APCs loaded with IgG-opsonised ovalbumin (OVA) *ex vivo* protects against challenge with OVA-expressing tumours in mice [6]."

The authors repeatedly point out lower necrotic debris uptake efficiency in cDC2 DCs. Their confocal data however from Fig. 3d confirms otherwise. At basal level the %CT-DR is undoubtedly higher in cDC1 however, it is noticeably higher in Hoxb8-cDC2 after WT Fc-DNGR-1. Can the authors point this out clearly and clarify whether there is a difference between Necrophagic index (Fig. 3b) and CT-DR% (Fig. 3d)?

We apologise for the confusion and believe this is due our experimental approaches, combined with qualitatively distinct forms of necrophagy between cDC1s and cDC2s. Necrophagic indices were calculated from flow cytometry data as outlined in methods: (% of CT-DR^+ x GMFI of CT-DR^+)/10,000. In contrast, confocal microscopy was calculated simply on % of CT-DR^+. This is because

by flow cytometry we can accurately quantify both *the frequency* of necrophagy and *the amount of internalised material* (GMFI). In contrast, by confocal microscopy we find that cDC1s "nibble" dead cell debris, which are internalised into small vesicles, making it difficult to quantify the total amount of fluorescence inside the cells. As such, we likely underestimate the necrophagic capacity of cDC1s in the microscopy assays. In contrast, cDC2s internalise whole necrotic cell corpses coated with Fc-DNGR-1, which is more easily quantifiable. We have added a line to this effect: "…as well as boosting the scavenging of small dead cell debris by cDC1s (Fig. 3c, d, Videos 1 and 2) although the latter was more difficult to detect by microscopy than by flow cytometry (cf. Fig. 3b and 3d).".

The idea that cDC2 DCs are CD4+ T cell activators is widely published in the field (PMID: 12093890, PMID: 2528589, PMID: 9892619). Although this might be outside the scope of the current manuscript, can the authors comment on this aspect in their discussion?

We agree that cDC2s play an important role in $CD4^+$ T cell priming and this may be an important aspect of how Fc-DNGR-1 supports anti-tumour immunity. We have altered the discussion to state "FcγR signalling also triggers myeloid cell activation and effective presentation on MHC-II to CD4+ T cells, which may further support the activity of cDC2s in an effective anti-tumour immune response."

Different DC populations exhibit varying degrees of maturity inside a tumor set up (PMID: 9892619). Can the authors add their thoughts on whether the type of FcγR engagement changes as a result?

We thank the reviewer for this interesting suggestion and now state in the Discussion: "Furthermore, how the activation state of distinct tumour APC populations impacts these FcγR functions warrants further investigation."

Reviewer #4
Remarks to the Author

By further mining their dataset the authors appropriately answered my question on the expression of CD80 and CD86 across cDC1s, cDC2s, and classical monocytes in human tumours. It turned out that all APCs analysed expressed similar levels of co-stimulatory molecules within tumours. Moreover, AFA-induced necrotic cell uptake was found capable of induce CD86 upregulation on human MCs in vitro. Importantly, the authors thoroughly answered all the questions raised by Reviewers 1 and 3 via the inclusion of a large amount of convincing new data. In my view this thorough and original study deserves publication as such in NatCancer.

We thank the reviewer for the positive appraisal of our study.

Minor
Lines 102-104: the term 'monocyte waterfall' was coined first by Tamoutounour et al. (DOI: 10.1002/eji.201242847) and not the provided Ref. 27-28.

We apologise for this error and now include the appropriate reference.

References

1. Rawat, K., et al., *CCL5-producing migratory dendritic cells guide CCR5+ monocytes into the draining lymph nodes.* Journal of Experimental Medicine, 2023. **220**(6).
2. Bosteels, C., et al., *Inflammatory Type 2 cDCs Acquire Features of cDC1s and Macrophages to Orchestrate Immunity to Respiratory Virus Infection.* Immunity, 2020. **52**.
3. Leach, S.M., et al., *Human and Mouse Transcriptome Profiling Identifies Cross-Species Homology in Pulmonary and Lymph Node Mononuclear Phagocytes.* Cell Reports, 2020. **33**(5): p. 108337-108337.

4. Canton, J., et al., *The receptor DNGR-1 signals for phagosomal rupture to promote cross-presentation of dead-cell-associated antigens.* Nature Immunology, 2021. **22**(2): p. 140-153.
5. Gonzales, G.A., et al., *The pore-forming apolipoprotein APOL7C drives phagosomal rupture and antigen cross-presentation by dendritic cells.* Science Immunology, 2024. **9**(101): p. 1-20.
6. Rafiq, K., A. Bergtold, and R. Clynes, *Immune complex–mediated antigen presentation induces tumor immunity.* Journal of Clinical Investigation, 2002. **110**(1): p. 71-79.

Reviewer #3 (Remarks to the Author):

The authors have revised all the concerns. While the authors state that they have improved the response stating “These results highlight how targeting FcγRs on tumour APCs may be leveraged therapeutically to boost CD8+ T cell responses to tumour antigens across species”, this reviewer was unable to locate this statement in the revised text. The authors may have chosen to leave this statement out again to avoid the impression that the human cross-presentation assays were performed following Fc-DNGR1 or anti-F-actin Affimer Fc fusion proteins. In other words, the proof of anti-tumor immunity in human requires future work which the authors make clear in their discussion. The authors have taken care to indicate "mouse" in various parts of the text when describing the results to separate what was done on mouse vs human cells. It is prudent to do so and avoid making strong statements about CD8 T cell responses across species.

We thank the reviewer for the positive assessment of our manuscript. We apologise for the confusion and confirm that we chose to revise this statement in light of our data, as noted by the reviewer. Rather, we state after section 1 that “FcγRs are highly expressed by non-cDC1 APCs in tumours and in inflamed tissues across species, allowing for uptake of IgG immune complexes and inducible XP to $CD8^+$ T cells in mice.”
