# Supplementary material for: Coupling dead cell recognition to Fcγ receptors augments anticancer immunity
Source: Nat Cancer. 2026 May 20;7(9):1350–67. doi: 10.1038/s43018-026-01168-5 (PMC13600936; doi:10.1038/s43018-026-01168-5)
Supplement: Supplementary file 4 [file 43018_2026_1168_MOESM7_ESM.pdf]

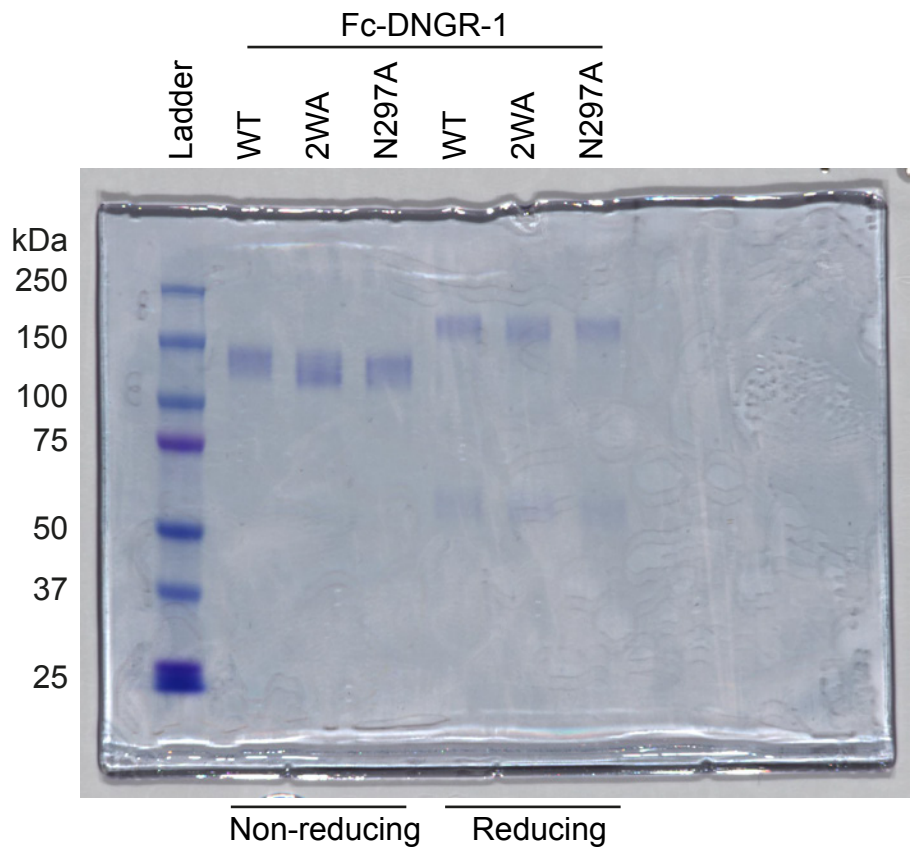

Related to Extended Data Figure 5b
